# Supplementary material for: Multimodal personalised executive function intervention (E-Fit) for school-aged children with complex congenital heart disease: protocol for a randomised controlled feasibility study
Source: BMJ Open. 2023 Nov 9;13(11):e073345. doi: 10.1136/bmjopen-2023-073345 (PMC10649522; doi:10.1136/bmjopen-2023-073345)
Supplement: Supplementary data [file bmjopen-2023-073345supp001.pdf]

UNIVERSITY
CHILDREN'S HOSPITAL
ZURICH

The hospital of the Eleonore Foundation

Focus group November

Discussion			
**Target group**	☐ Parents ☒ Children		
**Date / time / place**	23.11.2020	18.00-19.00	Zoom
**Lead**	Alenka Schmid, Interviewer 2, Interviewer 3		
**Topic**	☒ Intervention		
**Materials / Tools**	• Dodelido (Spiel)<br>• Matches	•	•
**Preparations**	Before:<br>• PowerPoint slides<br>• Create evaluation on Google Docs<br>• Create Collaboard Account (Interviewers: https://web.collaboard.app/projects)<br>• Ensure good lighting	During:<br>• Create Breakoutrooms	

Time	Description	Moderator
18.00-18.10	**Introduction**<br><br>Short greeting, introduction (first names), arriving and rename in Zoom<br>Explain why they are here today<br>Gallery view, mute when not speaking<br>Raise hand<br>Introductions (which animal would I like to be?)	Alenka
18.10-18.20<br><br>18:20-18:30<br><br>18:30-18:40	**Main part**<br><br>Introduction and info about brain training for EF and discussion framework via PowerPoint:<br><br>Different scenarios are presented (shopping and then forgetting, etc.).<br>Children can stretch out if this has happened to them before.<br><br>In a second step, they are asked to stretch out where they feel it happens to them more often than others.<br><br>Possible scenarios<br>- Anger/emotion control<br>- Planning/remembering (https://www.youtube.com/watch?v=o4zbeBcIAtU)<br>- Distractedness (https://www.youtube.com/watch?v=xhHkk1q04GA)<br>- Forgetfulness (https://www.youtube.com/watch?v=9TfJYgnXSus)<br><br>Presenting different possibilities<br>There is something different in each break-out room. Afterwards, the participants are mixed together so that someone from each group joins for the Disney method.<br>These are the following items:<br><br>1. Rubiks Cage (watch video, game with matches)	Alenka<br><br>Alenka<br><br>Interviewer 2

UNIVERSITY
CHILDREN'S HOSPITAL
ZURICH

The hospital of the Eleonore Foundation

<table>
<tr>
<td>18:40-
19:00

19:00</td>
<td>2. Cogmed (https://www.pearsonclinical.co.uk/Education/BestsellingInterventions/Cogmed/CogmedWorkingMemoryTraining.aspx; jeder für sich)
3. Dodelido

Disney Method (Breakout Rooms)
<table>
<tr><td>Dreamer</td><td>Participants are allowed to share and discuss their craziest ideas</td><td>5min</td></tr>
<tr><td>Critic</td><td>The participants reflect on what is feasible from their crazy ideas.</td><td>5min</td></tr>
<tr><td>Realist</td><td>The participants try to adapt their ideas so that they still have something to do with their dreams, but are more feasible from their point of view. Here they are also welcome to bring in their experiences from the previous breakout rooms.</td><td>10min</td></tr>
</table>
Return from Breakout rooms</td>
<td>Alenka

Interviewer 3

Interviewer 3, Interviewer 2</td>
</tr>
<tr>
<td>19:00-
19:05</td>
<td>Closing

Feedback (fill in online) and acknowledgement

Questions?</td>
<td>Alenka</td>
</tr>
</table>

**Goal of discussion?**	
	• Needs assessment
**Safety considerations**	• The event will be conducted online via Zoom

Details:

https://www.youtube.com/watch?v=xlqUjpPxcTk
Keep matches ready
Google form for evaluation:

- *In what time frame should the training take place (2 weeks/3 months)?*
- *How much time can the child (and parents) invest each day?*
- *Where should the training take place (at home, at school, at KiSpi, alone, in a group, with parents)?*
- *By what media (paper tasks, computer games, board games)?*
- *How much can or do you want parents to be involved in the training?*

2/2

UNIVERSITY
CHILDREN'S HOSPITAL
ZURICH

The hospital of the Eleonore Foundation

Focus group November

Discussion			
**Target group**	☒ Parents ☐ Children		
**Date / time / place**	23.11.2020	20.00-21.00	Zoom
**Lead**	Bea Latal, Alenka Schmid		
**Topic**	☒ Intervention		
**Materials / Tools**	• Dodelido<br>• Matches	•	•
**Preparations**	Before:<br>• PowerPoint slides	During:	

Time	Description	Moderator
	**Introduction**	
20.00-20.05	Short greeting, arriving (and rename on Zoom).	Bea
	Introductions (name and name of child, favorite vacation spot), write name and corona front to back.	Alenka
20:05-20:15	Participants are given the link to a Google Survey (or Wordcloud) via Zoom Chat. In it, they can openly write down their thoughts on various topics.<br>Topics:<br>Expectations for tonight<br>Particularly positive characteristics of your child?<br>Everyday problems of the child and with the child?<br>Wishes for further development of the child?	Bea
	**Main part**	
20.15-20.25	Introduction and information about EF at CHD and discussing framework via PowerPoint.	Alenka
20:25-20:35	Introductory question:<br>- Do you feel like a training of the executive functions could help your child?	
	Afterwards, the discussion is let run its course and moderated, if necessary, further questions are introduced.<br>Possible questions:<br><br>- What problems does your child have in everyday life?<br>- What do you expect from the training?<br>- What do you expect from the people who offer and conduct training?<br>- In what time frame should a training take place. How much time can the child and parents invest?<br>- At what age should it start?<br>- Where should the training take place, by what media? Paper tasks, computer games, alone, in group, at home, at the children's hospital?<br>- How much can or do you as parents want to participate in the training?<br>- Do you think your child would be motivated to do such a training?<br>- Are there other things for which training/intervention would be useful? (E.g., stress management, social graces, understanding the illness, exercise and physical activity?)	Bea

UNIVERSITY
CHILDREN'S HOSPITAL
ZURICH

The hospital of the Eleonore Foundation

20:35-20:55	Presentation of the team's ideas or direct demonstration (of the same things as to the children) and discussion of them	Alenka
20:55-21:00	**Closing**<br><br>Google Doc for evaluation<br><br>Acknowledgement and closing	 <br>Alenka<br><br>Bea

Goal of discussion?

	• Needs assessment
**Safety considerations**	• The event will be conducted online via Zoom
