# Supplementary material for: Multimodal personalised executive function intervention (E-Fit) for school-aged children with complex congenital heart disease: protocol for a randomised controlled feasibility study
Source: BMJ Open. 2023 Nov 9;13(11):e073345. doi: 10.1136/bmjopen-2023-073345 (PMC10649522; doi:10.1136/bmjopen-2023-073345)
Supplement: Supplementary data [file bmjopen-2023-073345supp007.pdf]

Table S1 Examples of item construction

Categories	Examples	Summary	Item
1 Identity/EF	*“But what I would certainly like for [my son] is … that he is trained a bit. … What should [he] do first, or how can [he] plan the day.”*	Daily planning as an everyday problem.	Children with congenital heart defects structure their everyday life
2 Identity/Relation to peers	*“he needs more time and he’s a bit slower and then he sees [his] siblings [and] that they’re always faster, and then he always makes himself small and that’s a shame.”*	Is slower than Peers which affects him negatively.	Children with congenital heart defects feel equal compared to their siblings/peers
3 Scope of intervention/game suggestions	*“analog games would also be OK, [I]can imagine that.”*	Analog games.	Dice games should be part of such an intervention.
4 Scope of intervention/structure	*“we would like it if it were possible online, because travelling … is always- … a bit far, yes. Especially during school time”*	Online is better for families who live further away from the hospital.	The intervention should be conducted online
5 Scope of intervention/possible distracting factors	*“they just have … a lot of homework …, then he plays the cello, then he goes to the youth club, everything interests him.”*	Other hobbies also need a lot of time and should still have room.	Hobbies could get in the way of the intervention
6 Scope of intervention/possible supporting factors	*“That it [should not be like] ‘oh that in addition,’ but ‘great, … that would be great right now and is manageable in terms of time’.”*	Time-manageable and fun.	This is the maximum amount of time per day the participants should work on the intervention (in free time):
7 Dealing with the disease/challenges	*“[that] is … something that … she finds quite difficult and where she probably swallows a lot.”*	She finds it difficult to talk about her illness and swallows a lot.	Children with congenital heart defects talk about their heart defect
8 Dealing with the disease/supporting factors	*“contact with other heart children, is certainly motivating.”*	Contact with other heart children can be motivating.	During the intervention, the children and adolescents should have contact with other children and adolescents
9 Further ideas	*“I think [contact with other children] will motivate them more than if they do their exercise in their corner [or] maybe talk to someone from your team from time to time”*	Contact with other children with CHD or with the study team	In such an intervention, there should be face-to-face meetings with the study team

Table S2 Timeline of the study protocol

	**Study timeline**				
	**Enrolment**	**Allocation**	**Post-Allocation**		
**TIMEPOINT**			***Baseline***	***+ 2 months***	***+ 6 months***
**ENROLMENT:**					
**Informed consent**	x				
**Eligibility screen**	x				
**Allocation**		x			
**INTERVENTIONS:**					
E-Fit Intervention			↔	↔	
Daily Diary			↔	↔	
**ASSESSMENTS:**					
IQ			x		
EF assessment			x	x	x
Questionnaires			x	x	x
Feasibility	x	x	↔	↔	
SES			x		
Medical information			x		

Note : EF = Executive function; SES = socioeconomic status

Table S3 Example week for a participating family

	Exemplar week					
	MO	***TUE***	***WED***	***THU***	***FRI***	***WEEKEND***
**E-FIT INTERVENTION**						
Parents	30min Call					
Child	60min Coaching Session[a]	20min CogniFit[b]		20min CogniFit[b]	20min CogniFit[b]	Card games
**CONTROL GROUP**						
Parents	10min Diary		10min Diary		10min Diary	10min Diary
Child	10min Diary		10min Diary		10min Diary	10min Diary

Note: a = Filling in Session Rating Scale; b = Filling in Children's Feeling scale and Fun and Demand

Table S4. Computerized training (CogniFit Inc © 2022)

Game	Trained skills			
	flexibility	working memory	planning	inhibition
Traffic Manager			x	
Water Lilies		x	x	
Reaction Field	x			x
Happy Hopper				x
Neuron Madness				x
Minus Malus	x	x		
Tennis Bowling	x			
Candy Line Up		x	x	
Bee Balloon	x			
Slice and Drop			x	
Cube Foundry			x	

Note: x *= primarily trained functions,* x *= additionally trained functions.*

Schmid AS, *et al. BMJ Open* 2023; 13:e073345. doi: 10.1136/bmjopen-2023-073345

Table S5. Analog training

Game	Trained skills				Players
	flexibility	working memory	planning	inhibition	
Gold (Game Factory ®)	x	x	x		2-5
Dodelido (“Drei Magier ®”)	x	x		x	2-6
Särge schubsen (“Drei Magier ®”)	x	x	x	x	2-6
Geistesblitz 2.0 (“Zoch”)	x	x		x	2-8
Beaver Gang (“Amigo Spiele”)	x	x	x		2-6
Beaver Clan (“Amigo Spiele”)	x	x	x		2-6
Memo Dice (“Amigo Spiele”)	x	x	x		2-4
5er finden (HABA ®)	x		x		1-4
The nasty 7 (“Drei Hasen in der Abendsonne”)	x	x		x	2-6
Yokai (Game Factory ®)	x	x	x		2-4
Okiya (“Pegasus Spiele”)	x		x		2
Perplexus Crazy run (2 PE Original, 5 FT 208, 6 PE Epic)				x	1
Thunder Pops				x	1

Games selected based on https://www.spielendfoerdern.ch/_files/ugd/8fa6b3_f84a167cfa3c405f8d3244a83242e7ac.pdf

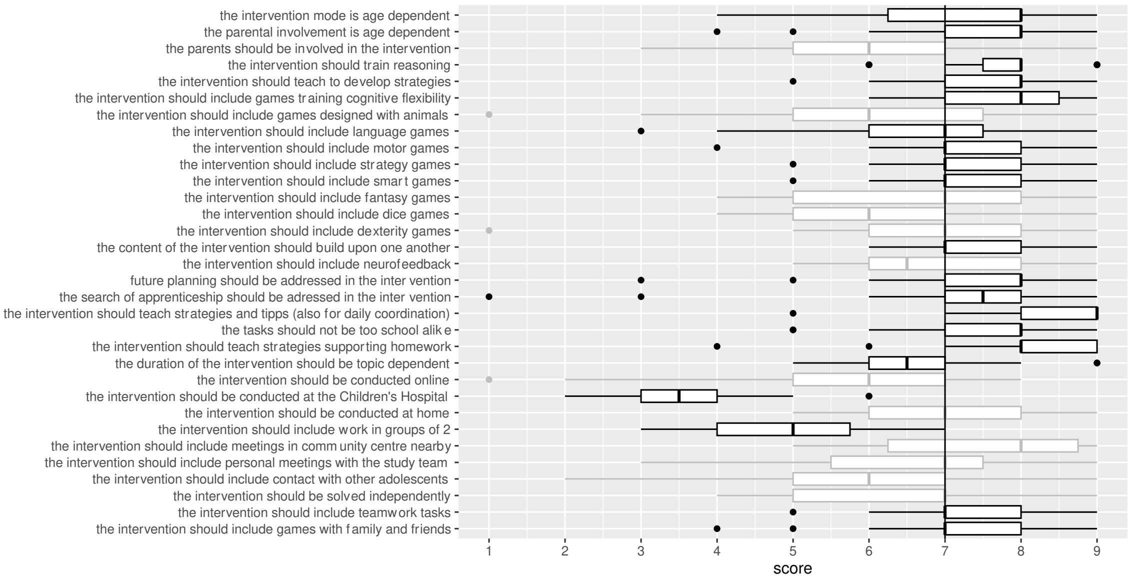

Figure S1. Ratings on Delphi items. 1 = fully disagree, 9 = fully agree. With a median of 7 and an IQR < 2 the item was considered accepted by the participants. Items in gray are the items for which no consensus was found.

6

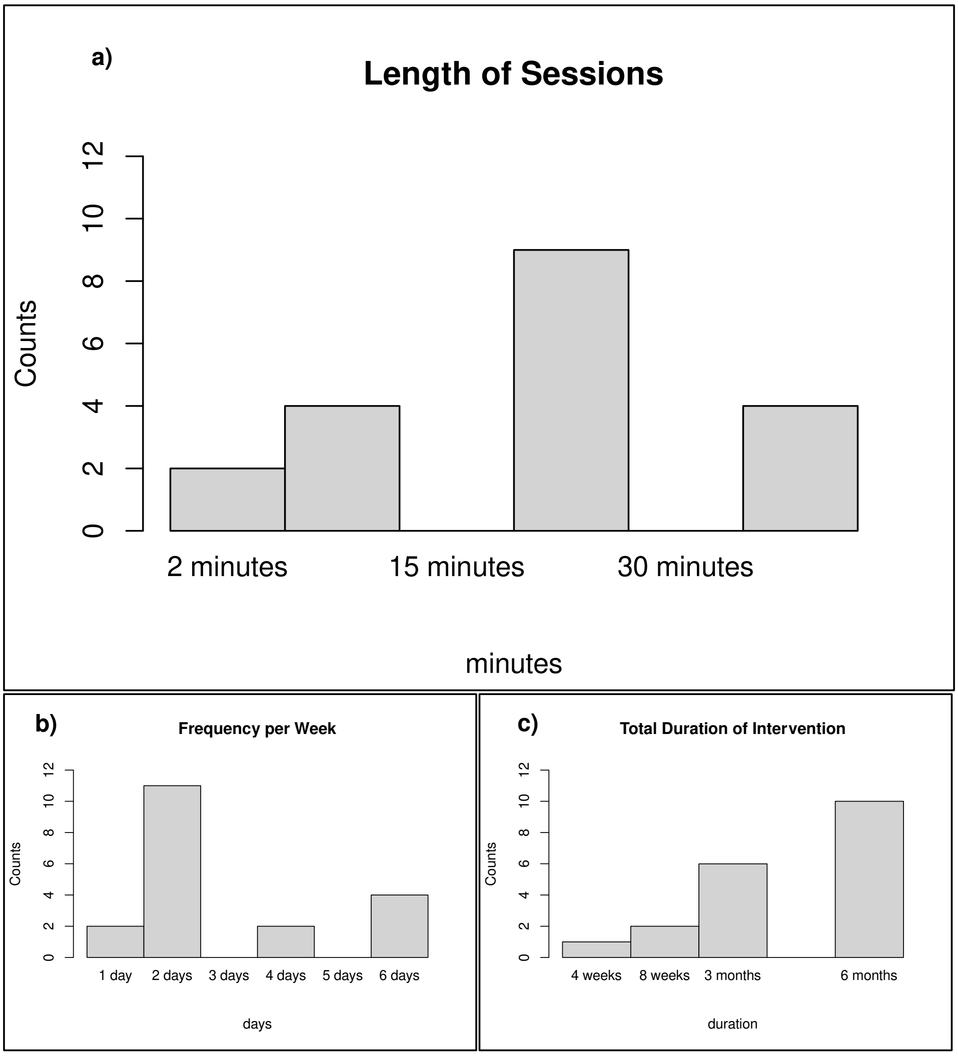

Figure S2. Counts of votes for a) length of sessions, b) frequency per week, and c) duration of the intervention.

7
