# Supplementary material for: Multimodal personalised executive function intervention (E-Fit) for school-aged children with complex congenital heart disease: protocol for a randomised controlled feasibility study
Source: BMJ Open. 2023 Nov 9;13(11):e073345. doi: 10.1136/bmjopen-2023-073345 (PMC10649522; doi:10.1136/bmjopen-2023-073345)
Supplement: Supplementary data [file bmjopen-2023-073345supp002.pdf]

A) Development of the Delphi survey

Sentences derived by qualitative content analysis from the focus groups were sorted into nine categories. Then, the statements were summarized and finally used to define the items in the Delphi survey. An example of statements and the consequent items is given in online supplemental table S1. The items are deliberately positively formulated. Some items were highly specific to children with CHD. Hence, the items for the “identity” and “dealing with the disease” categories were dropped for the Delphi survey. The final set of items included in the Delphi survey is provided in online supplemental figure S1.

B) Rationale behind the final intervention design

B.1) Virtual and analog games

Another element key to the success of any intervention is motivation [1]. Johann and Karbach compared six computer-based cognitive training programs [2]. They found evidence that playful interventions promote motivation and adherence in typically developing elementary school students. This reinforced the effects achieved by the EF training [2]. In addition, they emphasize the difference between computer-based training with added game elements and regular video games [3]: Videogames such as Super Mario and Fruit Ninja have no effect on EFs. In contrast, computer-based training with added game elements, such as in Lumosity® or CogniFit Inc © 2022 [4,5] shows clear benefits for EF improvement. In computer-based approaches, the training effect is maintained, and motivation is increased [3]. Because an intervention should be enjoyable to keep participants motivated, analog games such as board and card games have also been tested as cognitive training [6,7]. One obstacle associated with interventions using analog games is the mandatory involvement of other family or study members, which might overstrain time resources. However, participants of both the focus group and the Delphi survey indicated that it would be important to include analog games in the intervention. Hence, different analog games were added to the intervention, including games that can be played alone, and this part was decided to be open in terms of frequency and time spent on it. Moreover, whether the intervention should be online or analog was discussed without reaching a consensus by the Delphi survey participants. A systematic review and meta-analysis by Cao et al. found slight-to-moderate evidence for the effectiveness of computer-based interventions in promoting EF [3]. Computer-based interventions have been used in the population of CHD patients previously, and in the Delphi survey, only 4 of 12 participants disagreed with the online format. Hence, we decided that the intervention should include computer-based games. In addition, participating children could also use analog games. We decided to use existing computer-based and analog games instead of creating new games.

The intervention also includes analog and computerized games which, in addition to EF, promote eye-hand coordination, thus meeting a suggestion that motor games be included.

Schmid AS, *et al. BMJ Open* 2023; 13:e073345. doi: 10.1136/bmjopen-2023-073345

B.2) Strategy Coaching

The most highly rated items were related to learning strategies addressing everyday lives and homework. This is in line with the literature: another substantial shortcoming of many interventional studies is the lack of transfer [8–11]. Transfer refers to the degree of improvement in specific tasks requiring the same cognitive functions, termed near transfer, and in general cognitive abilities, termed far transfer [12]. A way to overcome a lack of transfer is by providing personal coaching sessions that support EF strategies in daily life and take into account children's emotional and social development [8]. Hence, we decided on adding strategy coaching. Career planning was a topic discussed in the Delphi survey. It was decided not to include this topic, as this is not appropriate for an age group of 10–12-year-old children.

B.3) Length, frequency per week and overall duration of the intervention

The most difficult aspect of the development of the intervention related to the minutes per day, frequency per week, and overall duration of the sessions. A wide range of possibilities was suggested (see online supplemental figure S2). These could not all be met. The length of the computerized training sessions was defined as 20 minutes, which was in agreement with the participants' suggestions. Other studies using session lengths of 20 minutes trained three to four times a week [13,14], which was a higher frequency than suggested by the families. Consequently, we decided on a frequency of three times a week and an overall duration of 8 weeks instead of the 6 months suggested (online supplemental figure S2).

Schmid AS, *et al. BMJ Open* 2023; 13:e073345. doi: 10.1136/bmjopen-2023-073345

1 Mohammed S, Flores L, Deveau J, *et al.* The Benefits and Challenges of Implementing Motivational Features to Boost Cognitive Training Outcome. *J Cogn Enhanc* 2017;**1**:491–507. doi:10.1007/s41465-017-0047-y
2 Johann VE, Karbach J. Effects of game-based and standard executive control training on cognitive and academic abilities in elementary school children. *Developmental Science* 2020;**23**:e12866. doi:10.1111/desc.12866
3 Cao Y, Huang T, Huang J, *et al.* Effects and Moderators of Computer-Based Training on Children's Executive Functions: A Systematic Review and Meta-Analysis. *Front Psychol* 2020;**11**:580329. doi:10.3389/fpsyg.2020.580329
4 Lumos Labs, Inc. Lumosity ®. 2022.
5 Cognifit. Cognifit Inc © 2022. 2022.
6 Estrada-Plana V, Esquerda M, Mangues R, *et al.* A Pilot Study of the Efficacy of a Cognitive Training Based on Board Games in Children with Attention-Deficit/Hyperactivity Disorder: A Randomized Controlled Trial. *Games for Health Journal* 2019;**8**:265–74. doi:10.1089/g4h.2018.0051
7 Gonçalves PD, Ometto M, Sendoya G, *et al.* Neuropsychological Rehabilitation of Executive Functions: Challenges and Perspectives. *Journal of Behavioral and Brain Science* 2014;**2014**. doi:10.4236/jbbs.2014.41004
8 Diamond A, Lee K. Interventions Shown to Aid Executive Function Development in Children 4 to 12 Years Old. *Science* Published Online First: 19 August 2011. doi:10.1126/science.1204529
9 Thorell LB, Lindqvist S, Bergman Nutley S, *et al.* Training and transfer effects of executive functions in preschool children. *Developmental Science* 2009;**12**:106–13. doi:10.1111/j.1467-7687.2008.00745.x
10 Diamond A, Ling DS. Conclusions about interventions, programs, and approaches for improving executive functions that appear justified and those that, despite much hype, do not. *Developmental Cognitive Neuroscience* 2016;**18**:34–48. doi:10.1016/j.dcn.2015.11.005
11 Gunzenhauser C, Nückles M. Training Executive Functions to Improve Academic Achievement: Tackling Avenues to Far Transfer. *Frontiers in Psychology* 2021;**12**.https://www.frontiersin.org/article/10.3389/fpsyg.2021.624008 (accessed 11 Mar 2022).
12 Barnett SM, Ceci SJ. When and where do we apply what we learn?: A taxonomy for far transfer. *Psychological Bulletin* 2002;**128**:612–37. doi:10.1037/0033-2909.128.4.612
13 Greco G. MULTILATERAL TRAINING USING PHYSICAL ACTIVITY AND SOCIAL GAMES IMPROVES MOTOR SKILLS AND EXECUTIVE FUNCTION IN CHILDREN WITH AUTISM SPECTRUM DISORDER. *European Journal of Special Education Research* Published Online First: 16 March 2020. doi:10.46827/ejse.v0i0.2928
14 Ramezan Saatchi L, Khodabakhsh Pirkalani R, Moradisabzevar M, *et al.* The effect of computerized Cognitive Training package on executive functions of people with major depressive disorder. *Iranian Journal of Psychiatric Nursing* 2022;**10**:39–52. doi:10.22034/IJPN.10.3.39
