# Supplementary material for: Multimodal personalised executive function intervention (E-Fit) for school-aged children with complex congenital heart disease: protocol for a randomised controlled feasibility study
Source: BMJ Open. 2023 Nov 9;13(11):e073345. doi: 10.1136/bmjopen-2023-073345 (PMC10649522; doi:10.1136/bmjopen-2023-073345)
Supplement: Supplementary data [file bmjopen-2023-073345supp003.pdf]

TEEN HEART STUDY E-FIT

UNIVERSITÄTS-KINDERSPITAL ZÜRICH
Forschungszentrum für das Kind FZK

Einwilligungserklärung

Schriftliche Einwilligungserklärung zur Teilnahme an einer klinischen Studie
Bitte lesen Sie dieses Formular sorgfältig durch. Bitte fragen Sie, wenn Sie etwas nicht verstehen oder wissen möchten. Für die Teilnahme ist Ihre schriftliche Einwilligung notwendig.

**BASEC-Nummer (nach Einreichung):**	
**Titel der Studie (wissenschaftlich und Laiensprache)**:	Executive functions intervention in children and adolescents with severe congenital heart disease: a feasibility study (E-Fit)<br><br>Intervention bei Kindern und Jugendlichen mit schweren angeborenen Herzfehlern: eine Machbarkeitsstudie (E-Fit)
**Verantwortliche Institution (Sponsor mit Adresse)**:	Prof. Dr. med. Bea Latal<br>Abteilung für Entwicklungspädiatrie<br>Universitäts-Kinderspital Zürich<br>Steinwiesstrasse 75, 8032 Zürich
**Ort der Durchführung**:	Universitäts-Kinderspital Zürich
**Prüfärztin/Prüfarzt am Studienort:**<br>Name und Vorname in Druckbuchstaben:	Prof. Dr. med. Bea Latal
**Teilnehmerin/Teilnehmer:**<br>Name und Vorname in Druckbuchstaben:<br>Geburtsdatum:	________________________<br>________________________<br>☐ weiblich ☐ männlich

- Ich und mein Kind wurden von der unterzeichnenden Studienkoordination mündlich und schriftlich über den Zweck, den Ablauf der Studie mit E-Fit über mögliche Vor- und Nachteile sowie über eventuelle Risiken informiert.
- Ich und mein Kind nehmen an dieser Studie freiwillig teil und akzeptiere den Inhalt der mir ausgehändigten schriftlichen Information. Ich und mein Kind hatten genügend Zeit, unsere Entscheidung zu treffen.
- Meine Fragen und die meines Kindes im Zusammenhang mit der Teilnahme an dieser Studie sind uns beantwortet worden. Ich und mein Kind behalten die schriftliche Information und erhalten eine Kopie unserer schriftlichen Einwilligungserklärung.
- Ich und mein Kind sind einverstanden, dass die Kinderärztin/der Kinderarzt meines Kindes über die Teilnahme an der Studie informiert wird.
- Ich und mein Kind sind einverstanden, dass die zuständigen Fachleute des Sponsors, der zuständigen Ethikkommission zu Prüf- und Kontrollzwecken in unsere unverschlüsselten Daten Einsicht nehmen dürfen, jedoch unter strikter Einhaltung der Vertraulichkeit.
- Bei Ergebnissen (und/oder Zufallsbefunden), die direkt die Gesundheit meines Kindes betreffen, werden ich und mein Kind informiert. Wenn ich und mein Kind das nicht wünschen, informieren wir die Studienkoordination.
- Ich und mein Kind wissen, dass die gesundheitsbezogenen und persönlichen Daten meines Kindes nur in verschlüsselter Form zu Forschungszwecken für diese Studie (auch im Ausland)

Schmid AS, *et al. BMJ Open* 2023; 13:e073345. doi: 10.1136/bmjopen-2023-073345

weitergegeben werden können. Der Sponsor gewährleistet, dass der Datenschutz nach Schweizer Standard eingehalten wird.

- Ich und mein Kind können jederzeit und ohne Angabe von Gründen von der Studienteilnahme zurücktreten. Die weitere medizinische Behandlung meines Kindes ist unabhängig von der Studienteilnahme gewährleistet. Die bis zum Rücktritt erhobenen Daten werden noch im Rahmen der Studie ausgewertet.
- Die Haftpflichtversicherung Universitäts-Kinderspital Zürich kommt für allfällige Schäden auf.
- Bei Zufallsbefunden möchten wir

  a) ☐ in jedem Fall informiert werden
  b) ☐ nicht informiert werden
  c) ☐ die Entscheidung folgender Person überlassen: ....

- Ich und mein Kind sind damit einverstanden, dass die Studienleitenden den aktuellsten kardiologischen Befund beim/bei der zuständigen Kardiolog:in einholen, um den Gesundheitszustand meines Kindes einzuschätzen.

  Name Kardiologe:___________________________

  Name Klinik: _______________________________

- Ich bin damit einverstanden, dass persönliche Daten von mir im Rahmen dieser Studie ausgewertet und verschlüsselt and Drittpersonen innerhalb des Universitäts-Kinderspitals Zürich weitergegeben werden können.
  ☐ Ja ☐ Nein

- Ich und mein Kind sind damit einverstanden, dass persönliche Daten verschlüsselt an Drittpersonen innerhalb des Universitäts-Kinderspitals Zürich weitergegeben werden können.
  ☐ Ja ☐ Nein

- Ich und mein Kind sind damit einverstanden, dass die Daten aus der neuropsychologischen Abklärung verschlüsselt zu Forschungszwecken an CogniFit© weitergegeben werden können.
  ☐ Ja ☐ Nein

- Ich und mein Kind sind damit einverstanden, dass während der neuropsychologischen Abklärung und der Intervention Videoaufnahmen gemacht und verschlüsselt abgespeichert werden.
  ☐ Ja ☐ Nein

- Ich und mein Kind sind damit einverstanden, dass die Hauptlehrperson über die Studienteilnahme informiert und miteinbezogen wird.
  ☐ Ja ☐ Nein

Schmid AS, *et al. BMJ Open* 2023; 13:e073345. doi: 10.1136/bmjopen-2023-073345

Die unterzeichnenden Personen bezeugen, dass das Aufklärungsgespräch stattgefunden hat und dass das urteilsfähige Kind in die Studienteilnahme eingewilligt hat und/oder sich keine Anzeichen von Widerstand gegen die Teilnahme erkennen lassen.

Ort, Datum	Unterschrift gesetzliche Vertretung des Kindes

Bestätigung der Studienkoordination: Hiermit bestätige ich, dass ich dieser Teilnehmerin/diesem Teilnehmer Wesen, Bedeutung und Tragweite der Studie erläutert habe. Ich versichere, alle im Zusammenhang mit dieser Studie stehenden Verpflichtungen gemäss in der Schweiz geltenden Rechts zu erfüllen. Sollte ich im Verlauf der Studie von Aspekten erfahren, welche die Bereitschaft der Teilnehmerin/des Teilnehmers zur Studienteilnahme beeinflussen könnten, werde ich sie/ihn umgehend darüber informieren.

Ort, Datum	Name und Vorname der Studienkoordination in Druckbuchstaben<br><br>Unterschrift der Studienkoordination
