# Supplementary material for: Multimodal personalised executive function intervention (E-Fit) for school-aged children with complex congenital heart disease: protocol for a randomised controlled feasibility study
Source: BMJ Open. 2023 Nov 9;13(11):e073345. doi: 10.1136/bmjopen-2023-073345 (PMC10649522; doi:10.1136/bmjopen-2023-073345)
Supplement: Supplementary data [file bmjopen-2023-073345supp004.pdf]

A) Computerized training (CogniFit Inc © 2022)

It is important to note that a contract was concluded with CogniFit Inc © 2022, because they provide discounts on their services in exchange for the pseudonymized baseline assessment data. Families had the choice whether to provide their data or not.

B) Strategy coaching

At the beginning of the intervention, parents complete a short questionnaire [1], assessing the behavior of their child in everyday life. The questionnaire was designed for parents to identify EF domains in which their children face difficulties. Lower scores indicate greater difficulties. Out of eleven EF domains rated, the six lowest scored are selected for the coaching sessions. Possible topics are working memory, attention control, emotion regulation, flexibility, initiating action, metacognition, organization, planning, response inhibition, time management and goal-directed persistence. In the first coaching session, the child's performance in the EF baseline assessment are discussed, and strengths and difficulties are identified [2]. Each of the next six sessions focuses on one of the six EF domains identified as most affected [1]. At the beginning of each session, the previous week is reviewed, focusing on the EF intervention and coaching instructions given at the previous session. Also, difficulties in regard to adherence are discussed and solutions suggested by the coach. In the second part of the session, a short story is read, featuring protagonists dealing with similar challenge as is the topic of that coaching session [3–6]. In the third part of the session, the focus is transferred to the child's situation by asking about how they could handle similar situations. Further, the child completes a task for the coaching session. Templates for these are available in Dawson et al.'s book Smart but scattered [1] and include checklist, plans, and tables. At the end of the session, the child is encouraged to use these strategies the following week, and the parents are informed by email about these strategies after the session. The parents receive fact sheets regarding the discussed EF domain of that particular session with tips for everyday life and a short summary on what was done in the session. All parental tips are also taken from the German version of Smart but scattered [1].

1 Dawson P, Guare R. *Schlau aber...* 1st ed. Bern: : Verlag Hans Huber, Hogrefe AG 2012.
2 Leiss U. Die neuropsychologische Ergebnisbesprechung als Intervention – Eine Brücke zwischen Befund und Alltag. In: Pletschko T, Leiss U, Pal-Handl K, *et al.*, eds. *Neuropsychologische Therapie mit Kindern und Jugendlichen: Praktische Behandlungskonzepte bei neurokognitiven Funktionsstörungen*. Berlin, Heidelberg: : Springer 2020. 35–43. doi:10.1007/978-3-662-59288-5_4
3 Liebers A, Kubesch S. *Ob Gespenter Fußball spielen?* 1st ed. Verlag Bildung Plus 2014.
4 Liebers A, Kubesch S. *Mathetest und Drachenhörnchen*. 1st ed. Verlag Bildung Plus 2013.
5 Liebers A, Kubesch S. *Ein Ritter in der Klasse*. 1st ed. Verlag Bildung Plus 2013.
6 Liebers A, Kubesch S. *Stopp oder es kracht!* Verlag Bildung Plus 2014.
