# Supplementary material for: Multimodal personalised executive function intervention (E-Fit) for school-aged children with complex congenital heart disease: protocol for a randomised controlled feasibility study
Source: BMJ Open. 2023 Nov 9;13(11):e073345. doi: 10.1136/bmjopen-2023-073345 (PMC10649522; doi:10.1136/bmjopen-2023-073345)
Supplement: Supplementary data [file bmjopen-2023-073345supp005.pdf]

TEEN HEART STUDY E-FIT

Acceptance and Feasibility Scale Parents

	Fully disagree			Fully agree
The program was useful for my child.	☐	☐	☐	☐
My child would do the program again.	☐	☐	☐	☐
I would recommend this program to other children.	☐	☐	☐	☐
My child has applied the skills learned.	☐	☐	☐	☐
My child has a plan for dealing with future problems.	☐	☐	☐	☐
My child has made positive changes.	☐	☐	☐	☐
My child has used the skills to become more organized.	☐	☐	☐	☐
My child has used reminder strategies to remember things more often.	☐	☐	☐	☐
My child uses techniques to plan.	☐	☐	☐	☐
My child has met his or her program goals.	☐	☐	☐	☐
My child has a more positive attitude than before.	☐	☐	☐	☐
My child is better at solving problems.	☐	☐	☐	☐
My child remembers things better.	☐	☐	☐	☐
My child is more organized.	☐	☐	☐	☐
My child is better at paying attention and remembering things.	☐	☐	☐	☐
My child is better at getting things done, such as homework or chores.	☐	☐	☐	☐
My child is better at controlling his or her emotions.	☐	☐	☐	☐
My child is better at controlling his or her actions.	☐	☐	☐	☐
My child is better at stopping and thinking before acting.	☐	☐	☐	☐
My child is better able to handle unforeseen changes in plans.	☐	☐	☐	☐
My child feels less stressed.	☐	☐	☐	☐
My child does better in school.	☐	☐	☐	☐

Acceptance and Feasibility Scale

adapted after Modi et al., 2019

TEEN HEART STUDY E-FIT

Acceptance and Feasibility Scale Children

	Fully disagree			Fully agree
Everything worked as it should.	☐	☐	☐	☐
It was easy to use the online website for the games.	☐	☐	☐	☐
I liked the online games.	☐	☐	☐	☐
It was easy to get in touch with the coach through Microsoft Teams/Zoom.	☐	☐	☐	☐
Meeting with a coach in person would be better.	☐	☐	☐	☐
I felt connected to the coach.	☐	☐	☐	☐
I felt that the coach contributed to my learning beyond the sessions.	☐	☐	☐	☐
The information from the coaching sessions was helpful to me.	☐	☐	☐	☐
I enjoyed the coaching sessions.	☐	☐	☐	☐
I enjoyed the analogue games.	☐	☐	☐	☐
It was easy to understand the game instructions (videos and or brain cards).	☐	☐	☐	☐
The program (as a whole) was too long.	☐	☐	☐	☐
The program (as a whole) was too short.	☐	☐	☐	☐
The program (as a whole) was as I expected it to be.	☐	☐	☐	☐
I liked the program (as a whole).	☐	☐	☐	☐
The content was not relevant to me.	☐	☐	☐	☐
The program (as a whole) was useful to me.	☐	☐	☐	☐
I would do the program (as a whole) again.	☐	☐	☐	☐
I would recommend the program (as a whole) to other children.	☐	☐	☐	☐
What part of the program did you enjoy the most?	☐	☐	☐	☐
What part of the program helped you the most?	☐	☐	☐	☐

	no	yes		
Could any part be dropped?	☐	☐	If yes which one?	

TEEN HEART STUDY
E-FIT

	Fully disagree			Fully agree
I applied the skills I learned during the program.	☐	☐	☐	☐
I have a plan for dealing with future problems.	☐	☐	☐	☐
I made positive changes.	☐	☐	☐	☐
I have used skills to get more organized.	☐	☐	☐	☐
I have used remembering strategies to remember things better.	☐	☐	☐	☐
I have used techniques to plan.	☐	☐	☐	☐
I have achieved the goals I had at the beginning of the program.	☐	☐	☐	☐
I have a more positive attitude than before.	☐	☐	☐	☐
I am better at solving problems.	☐	☐	☐	☐
I am better at remembering things.	☐	☐	☐	☐
I am more organized.	☐	☐	☐	☐
I know strategies that help me pay attention and remember.	☐	☐	☐	☐
I start things, like homework or chores, better than I used to.	☐	☐	☐	☐
I can control my emotions better than I used to.	☐	☐	☐	☐
I can control my actions better than I used to.	☐	☐	☐	☐
I am better able to stop and think before I act.	☐	☐	☐	☐
I can better cope with unforeseen changes in plans.	☐	☐	☐	☐
I feel less stressed.	☐	☐	☐	☐
I do better in school.	☐	☐	☐	☐

Acceptance and Feasibility Scale *adapted after Modi et al., 2019*

Everyday Activities Diary

List of activities: School *1 box = 1 quarter of an hour*

What did your child do today?

Please indicate your child’s activities today (to the nearest quarter of an hour)

Activity		06:00	07:00	08:00	09:00	10:00	11:00	12:00
**General**								
Sleeping								
Eating								
**School**								
Lessons								
Physical education								
Recess								
**Leisure time**								
Homework								
Reading or browsing through books on his/her own								
Playing a musical instrument or singing								
Watching TV, videos or DVDs								
Computer or video games, Playstation, Nintendo or the Internet								
Other quite activities or games (listening to music or stories, drawing, painting, handiwork or board games)								
Relatively vigorous games with friends or siblings (playground, role-playing, hide-and-seek, etc.)	Indoor							
	Outdoor							
Vigorous games, running around or ball games (not in a sports club)	Indoor							
	Outdoor							
Training, riding or ballet (in a club)	Indoor							
	Outdoor							
**Travel**								
By foot								
By bicycle, scooter, inline skates or skateboard								
By train, tram or bus								
By car								
**Special**								
Activity difficult to classify								

		12:00	13:00	14:00	15:00	16:00	17:00	18:00	19:00	20:00	21:00	22:00	23:00	24:00	01:00	02:00	03:00	04:00	05:00	06:00
**General**																				
Sleeping																				
Eating																				
**School**																				
Lessons																				
Physical education																				
Recess																				
**Leisure time**																				
Homework																				
Reading or browsing through books on his/her own																				
Playing a musical instrument or singing																				
Watching TV, videos or DVDs																				
Computer or video games, Playstation, Nintendo or the Internet																				
Other quite activities or games (listening to music or stories, drawing, painting, handiwork or board games)																				
Relatively vigorous games with friends or siblings (playground, role-playing, hide-and-seek, etc.)	Indoor																			
	Outdoor																			
Vigorous games, running around or ball games (not in a sports club)	Indoor																			
	Outdoor																			
Training, riding or ballet (in a club)	Indoor																			
	Outdoor																			
**Travel**																				
By foot																				
By bicycle, scooter, inline skates or skateboard																				
By train, tram or bus																				
By car																				
**Special**																				
Activity difficult to classify																				

UNIVERSITÄTS-
KINDERSPITAL
ZÜRICH
Forschungszentrum
für das Kind FZK

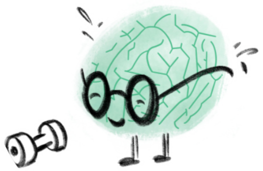

Coaching Content Preparatory Session

**Timepoint**	**Content**		**Item number**
	Explained why we did the assessment	☐ yes ☐ no	1
	Neuroinfo for Kids (NIK) page 3 read aloud	☐ yes ☐ no	2
	Strengths and weaknesses sheet filled in	☐ yes ☐ no	3
	NIK page 5 read aloud	☐ yes ☐ no	4
	NIK page 6 read aloud	☐ yes ☐ no	5
	Results discussed on normal distribution	☐ yes ☐ no	6
	NIK results (2) page read aloud	☐ yes ☐ no	7
	NIK page 10 read aloud	☐ yes ☐ no	8
	Letter written	☐ yes ☐ no	9
	Duration of session 60min	☐ yes ☐ no<br>If no, duration in min: ______	10

E-Fit Fidelity Measurement System

Coaching Content EF Sessions 1-6

**Timepoint**	**Content**		Item number
	Question asked:<br>Were you able to try out what we talked about last week?	☐ yes ☐ no	10
	Question asked:<br>Did you play the card games? (Which ones?)	☐ yes ☐ no	11
	Question asked:<br>Did everything work out with the online games? I saw you played 3x last week and always started 2x, great! (if necessary point out if something was forgotten)	☐ yes ☐ no	12
	Question asked:<br>Did you add the stickers to your poster?	☐ yes ☐ no	13
	Session topic explained	☐ yes ☐ no	14
	Session goal explained	☐ yes ☐ no	15
	All listed pages of the story read aloud/viewed	☐ yes ☐ no	16
	Assignment completed as described in the session guideline	☐ yes ☐ no	17
	Homework assigned	☐ yes ☐ no	18
	Session duration 60min	☐ yes ☐ no<br>If no, duration in min:<br>____________	19

E-Fit Fidelity Measurement System

Coaching Content Last Session

**Timepoint**	**Content**		**Item number**
	Question asked:<br>Were you able to try out what we talked about last week?	☐ yes ☐ no	10.7
	Question asked:<br>Did you play the card games? (Which ones?)	☐ yes ☐ no	11.7
	Question asked:<br>Did everything work out with the online games? I saw you played 3x last week and always started 2x, great! (if necessary point out if something was forgotten)	☐ yes ☐ no	12.7
	Question asked:<br>Did you add the stickers to your poster?	☐ yes ☐ no	13.7
	Overview of this session provided	☐ yes ☐ no	20
	Summary of the last 8 weeks	☐ yes ☐ no	21
	Evaluation questions asked according to guideline	☐ yes ☐ no	22
	How to continue explained according to guideline	☐ yes ☐ no	23
	Questions and comments of the family clarified	☐ yes ☐ no	24
	Session duration 60min	☐ yes ☐ no<br>If no, duration in min:<br>____________	19.7

E-Fit Fidelity Measurement System

Computerized Games

Date	Content		Item number
	Sessions 1&2 completed	☐ yes ☐ no	25
	Sessions 3&4 completed	☐ yes ☐ no	26
	Sessions 5&6 completed	☐ yes ☐ no	27
	Sessions 7&8 completed	☐ yes ☐ no	28
	Sessions 9&10 completed	☐ yes ☐ no	29
	Sessions 11&12 completed	☐ yes ☐ no	30
	Sessions 13&14 completed	☐ yes ☐ no	31
	Sessions 15&16 completed	☐ yes ☐ no	32
	Sessions 17&18 completed	☐ yes ☐ no	33
	Sessions 19&20 completed	☐ yes ☐ no	34
	Sessions 21&22 completed	☐ yes ☐ no	35
	Sessions 23&24 completed	☐ yes ☐ no	36
	Sessions 25&26 completed	☐ yes ☐ no	37

Schmid AS, *et al. BMJ Open* 2023; 13:e073345. doi: 10.1136/bmjopen-2023-073345

Analogue Games

**Duration**	**Content**		**Item number**
	Gold	☐ yes ☐ no	38
	Dodelido	☐ yes ☐ no	39
	Särge schubsen	☐ yes ☐ no	40
	Geistesblitz 2.0	☐ yes ☐ no	41
	Beaver Gang	☐ yes ☐ no	42
	Beaver Clan	☐ yes ☐ no	43
	Memo Dice	☐ yes ☐ no	44
	5-er finden	☐ yes ☐ no	45
	The nasty 7	☐ yes ☐ no	46
	Yokai	☐ yes ☐ no	47
	Okiya	☐ yes ☐ no	48
	Perplexus Crazy run 2 PE	☐ yes ☐ no	49
	Perplexus Crazy run 5 FT 208	☐ yes ☐ no	50
	Perplexus Crazy run 6 PE Epic	☐ yes ☐ no	51
	Thunder Pops	☐ yes ☐ no	52
