# Supplementary material for: Multimodal personalised executive function intervention (E-Fit) for school-aged children with complex congenital heart disease: protocol for a randomised controlled feasibility study
Source: BMJ Open. 2023 Nov 9;13(11):e073345. doi: 10.1136/bmjopen-2023-073345 (PMC10649522; doi:10.1136/bmjopen-2023-073345)
Supplement: Supplementary data [file bmjopen-2023-073345supp006.pdf]

Supplementary information for the SPIRIT Guidelines

A) Dataset information

Data category	Information
Primary registry and trial identifying number	ClinicalTrials.gov<br>NCT05198583
Date of registration in primary registry	19 January, 2022
Source(s) of monetary or material support	Swiss National Science Foundation (SNF 32003B_172914)
Contact for public & scientific queries	Prof. Dr. Beatrice Latal; bea.latal@kispi.uzh.ch
Scientific title	Multimodal personalized executive function intervention (E-Fit) for school-age children with complex congenital heart disease: protocol for a randomized controlled feasibility study
Country of recruitment	Switzerland
Health condition(s) or problem(s) studied	Executive dysfunction, severe congenital heart disease
Intervention(s)	Intervention group: executive function intervention (E-Fit)<br>Control group: electronic diary
Key inclusion and exclusion criteria	Ages eligible for study: 10-12 years<br>Sexes eligible for study: both<br>Accepts healthy volunteers: no<br>Inclusion criteria: child born with congenital heart disease (10-12 years), cardiopulmonary bypass surgery prior to one year of age, executive function (EF) deficits<br>Exclusion criteria: heart surgery not performed in Switzerland, known genetic or dysmorphic syndrome
Study type	Interventional allocation: randomized<br>Intervention model: parallel assignment<br>Masking: single blind, outcome assessors<br>Primary purpose: feasibility<br>Phase I
Date of first enrolment	April 2022
Target sample size	40
Recruitment status	Recruiting
Primary Outcome(s)	Feasibility
Key secondary outcomes	Executive function performance

Schmid AS, *et al. BMJ Open* 2023; 13:e073345. doi: 10.1136/bmjopen-2023-073345

B) Organizational structure and responsibilities

Principal Investigator: Beatrice Latal, Child Development Center, University Children's Hospital Zurich Switzerland

Preparation of protocol and revisions
Publication of study reports

Lead Investigator: Alenka Schmid, Child Development Center, University Children's Hospital Zurich Switzerland

Responsible for identification, recruitment, data collection, and completion of case report forms (CRFs), along with patients adherence to study protocol. Responsible for maintenance of trial IT system and data entry, data verification, and randomization
Responsible for trial master file
Recruitment of patients and liaising with principal investigator
Preparation of CRFs
Design and conduct of E-Fit Feasibility Study

Authorship eligibility guideline

Authors on this paper made a significant contribution to the conception or design of the project or the acquisition, analysis, or interpretation of data for the work, and/or drafted the work or reviewed and revised it critically for important intellectual content.

C) Data management and safety monitoring

Study and questionnaire data are collected by physicians, psychologists, or psychologists in training and recorded in Redcap® [1]. Data from routine visits are derived by a physician from the electronic medical charts of the hospital's data management system, Phoenix, with access limited to the staff of the Children`s Hospital and with strict regulation of data protection. Any prints are locked in a drawer, with access only by the investigator.
Members of the Children's Research Centre of the University Children's Hospital Zurich fulfil the monitoring duties. This includes double checking the data, prestudy monitoring, after enrolment of the 2nd patient, after the 20th patient and a close-out visit. Monitoring covers all study documents, source data, and the trial master file. No interim analyses are planned. This trial is considered as a nonsignificant risk device study and was reviewed accordingly by the Cantonal Ethics Committee of Zurich.
Parents and participants may withdraw from participation at any time. Families are contacted by phone to ascertain the reason for discontinuation as this is important information for the primary outcome of the present study, feasibility. All data collected until drop out will be used for analysis. Serious events and serious adverse events are reported in accordance with the guidelines of the Zurich Cantonal

Ethics Committee, based on the Swiss Federal Act on Research Involving Human Beings. Protocol modifications and amendments will be submitted to the ethical committees for approval. Amendments to the study protocol will be added to publications reporting the study outcomes.

1 Wright A. REDCap: A Tool for the Electronic Capture of Research Data. *J Electron Resour Med Libr*. 2016;13:197–201.
