# Supplementary material for: Multimodal personalised executive function intervention (E-Fit) for school-aged children with complex congenital heart disease: protocol for a randomised controlled feasibility study
Source: BMJ Open. 2023 Nov 9;13(11):e073345. doi: 10.1136/bmjopen-2023-073345 (PMC10649522; doi:10.1136/bmjopen-2023-073345)
Supplement: Author's manuscript [file bmjopen-2023-073345.draft_revisions.pdf]

BMJ Open is committed to open peer review. As part of this commitment we make the peer review history of every article we publish publicly available.

When an article is published we post the peer reviewers' comments and the authors' responses online. We also post the versions of the paper that were used during peer review. These are the versions that the peer review comments apply to.

The versions of the paper that follow are the versions that were submitted during the peer review process. They are not the versions of record or the final published versions. They should not be cited or distributed as the published version of this manuscript.

BMJ Open is an open access journal and the full, final, typeset and author-corrected version of record of the manuscript is available on our site with no access controls, subscription charges or pay-per-view fees (http://bmjopen.bmj.com).

If you have any questions on BMJ Open's open peer review process please email info.bmjopen@bmj.com

Multimodal personalized executive function intervention (E-Fit) for school-age children with complex congenital heart disease: protocol for a randomized-controlled feasibility study.

Journal:	*BMJ Open*
Manuscript ID	bmjopen-2023-073345
Article Type:	Protocol
Date Submitted by the Author:	08-Mar-2023

Complete List of Authors:	Schmid, Alenka; Universitäts-Kinderspital Zürich,<br>Ehrler, Melanie; University Children Hospital Zurich, Child Development Center; University Children's Hospital Zurich, Children's Research Center<br>Wehrle, Flavia; University Children’s Hospital Zurich, Child Development Center; University Children’s Hospital Zurich, Children’s Research Center<br>Tuura, Ruth; University Children's Hospital of Zurich, Child Development Center; University Children's Hospital Zurich, Children's Research Center<br>Kretschmar, Oliver; Universitäts-Kinderspital Zürich, Department of Pediatric Cardiology; University of Zurich Faculty of Medicine,<br>Landolt, Markus; University Children's Hospital Zurich, Department of Psychosomatics and Psychiatry; University of Zurich, Division of Child and Adolescent Health Psychology<br>Latal, Beatrice; University Children’s Hospital Zurich, Child Development Center; University of Zurich

Keywords:	Feasibility Studies, Developmental neurology & neurodisability < PAEDIATRICS, Congenital heart disease < CARDIOLOGY, Adolescent

SCHOLARONE™
Manuscripts

Multimodal personalized executive function intervention (E-Fit) for school-age children with complex congenital heart disease: protocol for a randomized-controlled feasibility study.

Alenka Schmid[1], Melanie Ehrler[1,2,3], Flavia M. Wehrle[1,2,5], Ruth O'Gorman Tuura[1,2,4,6], Oliver Kretschmar[2,4,7], Markus A. Landolt[2,8,9], Beatrice Latal[1,2,3,4]

[1]Child Development Center, University Children's Hospital Zurich, Zurich, Switzerland

[2]Children's Research Center, University Children's Hospital Zurich, Zurich, Switzerland

[3]URPP Adaptive Brain Circuits in Development and Learning, University of Zurich, Zurich, Switzerland

[4]University of Zurich, Zurich, Switzerland

[5]Department of Neonatology and Intensive Care, University Children's Hospital Zurich, Zurich, Switzerland

[6]MR Research Centre, University Children's Hospital Zurich, Zurich, Switzerland

[7]Department of Cardiology, University Children's Hospital Zurich, Zurich, Switzerland

[8]Department of Psychosomatics and Psychiatry, University Children's Hospital Zurich, Zurich, Switzerland

[9]Division of Child and Adolescent Health Psychology, Department of Psychology, University of Zurich, Zurich, Switzerland

ORCID iDs

Alenka Schmid https://orcid.org/0000-0001-5013-0530

Markus A. Landolt https://orcid.org/0000-0003-0760-5558

Correspondence to

Prof. Dr. Beatrice Latal; bea.latal@kispi.uzh.ch

Child Development Center

University Children's Hospital Zurich

Steinwiesstrasse 75, 8032 Zurich, Switzerland

Protocol version

02.03.23, Version 1.0

Competing interests

The authors have no financial or other competing interests for the overall study.

Keywords: Feasibility Studies; Developmental neurology & neurodisability; Congenital heart disease; Adolescent

For peer review only

Abstract

Introduction

Children with congenital heart disease (CHD) are at risk of neurodevelopmental problems, and executive functions (EFs) are the functions most frequently affected. To date, interventions have limited effects on EFs in children and adolescents with complex CHD. Therefore, we developed a new multimodal and personalized EF intervention which is currently being tested in a randomized-controlled feasibility study.

Methods and analysis

This study includes children with CHD who underwent cardiopulmonary bypass surgery prior to one year of age. Children are 10 to 12 years at enrollment and show clinically relevant EF impairments (age and sex referenced T-score ≥ 60 on any of the three summary scales of the Behavior Rating Inventory for Executive Function questionnaire). The multimodal intervention was developed with focus groups and a Delphi survey involving patients, their parents and teachers, and health professionals. The intervention is composed of three elements: computer-based EF training using CogniFit Inc © 2022, performed three times a week at home; weekly EF remote strategy coaching; and analog games. The content of the computer and strategy training is personalized to patients' individual EF difficulties. The control group follows their daily routines as before and completes a diary about their everyday activities four times a week. The primary outcome of this study is the intervention's feasibility measured by the participants' and providers' ratings of the participants' adherence and exposure to the intervention, recruitment rates, and the evaluation of the intended effects of the program.

Ethics and Dissemination

The project plan, participant information and consent form, and other project-specific documents were approved by the local ethics committee (BASEC-Nr: 2021-02413).

Registration details

ClinicalTrials.gov: NCT05198583

Article Summary

Strengths and Limitations

- This is the first study to assess the feasibility of a novel multimodal personalized EF intervention for children with complex CHD. This study will show whether the E-Fit is feasible for patients with CHD and whether the study design can be used for a regular randomized controlled trial.
- The intervention is tailored towards patients' and families' needs which were identified with focus groups and a Delphi survey incorporating the inputs of patients, families, teachers, and health professionals.

For peer review only

- Due to the information procedure, the families of the control group are contaminated: they know what the intervention consists of and might engage in the proposed activities even when in the control group.
- The primary outcome of the present study is feasibility. The effectiveness of the training will be estimated in an exploratory analysis because of the limited sample size.

For peer review only

1. Introduction

Eight out of 1000 children are born with congenital heart disease (CHD; [1]); of these, one third require open heart surgery during infancy [2]. In these patients, neurodevelopmental problems are known to be a main comorbidity [3]. Studies have shown that the mean IQ of children with CHD is within the normal range but lower than that of their healthy peers [4]. Despite this normal intellectual functioning, children with complex CHD, particularly, are at risk for higher-order cognitive deficits: executive function (EF) impairments [4,5]. The main EFs are inhibition; cognitive flexibility, including fluency; planning; and working memory [6–10]. Children with CHD show mild to moderate impairments in all these EF domains [4,5] without a clear predominance of any specific EF domain. In typically developing children, EFs are more closely related to school readiness even than IQ, and EFs predict academic achievement [11]. Accordingly, EF difficulties in childhood have been related to higher unemployment rates in adulthood [12]. Further, EF difficulties in adulthood can increase the risk of addictive behavior, and psychiatric diseases such as anxiety and depression [13,14].

Many sequelae of EF difficulties have also been demonstrated in patients with CHD. They face lower school achievement and lower psychosocial quality of life (QoL) during school age [15,16]. Further, lower EFs have been associated with a higher rate of internalizing symptoms in adolescents and adults with CHD [17]. Given the lifelong academic, psychological, and social burden associated with EF difficulties, evidence-based interventions improving EF functions are urgently needed, particularly for children and adolescents with CHD [18,19].

1.1 EF Intervention

To date, only two studies have investigated the efficacy of EF interventions in patients with CHD. Both studies included school-aged children and adolescents with CHD and tested Cogmed Working Memory Training (Cogmed; [20,21]). Cogmed is a computerized program designed to improve attention and working memory, a core dimension of EF [22]. Participants completed regular Cogmed sessions at home over for at least 5 weeks. These studies demonstrated inconsistent findings: Calderon et al. found no improvement of working memory either at postintervention or at 3-month follow up, but patients who did the Cogmed sessions showed significant improvements in inhibitory control and self-regulation at the 3-month follow up. Parents reported improved cognitive regulatory skills at postintervention and at 3-month follow up [20].

In contrast, Jordan et al. found improved working memory postintervention in patients who were assigned to Cogmed compared to the control group. However, this improvement did not remain further than 6-month follow up [21]. These findings are in line with the inconsistent results of a recent meta-analysis of EF interventions in

For peer review only

other children at risk for EF impairments [23]. That study reported only small to moderate effects of computerized interventions of single functions and no long-term effects.

Intervention approaches other than computerized training should be tested for potential beneficial effects on executive functions [20]. Mindfulness interventions and strategy coaching may address transfer to everyday lives by targeting sensory awareness and attention regulation, thereby promoting emotional and social development [18,24]. Mindfulness education has improved EF behavior, optimism, and classroom social competence behaviors in healthy children and adolescents [25,26]. Furthermore, problem-solving solutions to EF-related challenges in everyday life improved parent- and self-rated global EF behaviors in children with epilepsy [27].

An intervention should be enjoyable to keep participants motivated. Hence, analog games such as board and card games have also been tested as cognitive interventions. They have proven effective in enhancing the functions trained, such as problem-solving. Moreover, by requiring children to play with others, such games may enhance self-monitoring [28]. Combinations of computer and analog games have shown improvements in trained reasoning and speed tasks in healthy children with low socioeconomic status aged 7 to 10 years [29]. Because children with CHD experience difficulties in various EF domains with significant academic, psychological, and social consequences, an intervention integrating several of these approaches may be particularly suited to improving these children's EFs and, eventually, their long-term development and social integration.

1.2 Aims

This study aims to test the feasibility of a multimodal personalized EF intervention called E-Fit for children with complex CHD and EF impairments. To evaluate E-Fit's feasibility, the following hypotheses will be explored:

- Children with CHD and EF impairments adhere to all components of the E-Fit intervention
- Children with CHD and EF impairments and their parents find all components of the intervention suitable and enjoyable and indicate a high level of satisfaction with the E-Fit intervention
- Children with CHD and EF impairments can integrate all components of the E-Fit intervention
- Although exploratory due to its small sample size, the E-Fit intervention shows potential for improving EFs in children with CHD and EF impairments

For peer review only

2. Methods and analyses

2.1 Intervention development

Before developing E-Fit and the present study, we conducted a needs assessment to address the unique EF needs of children with CHD.

2.1.1 Focus group interviews

We conducted two separate online focus group interviews: one with adolescents with CHD aged 14 to 16 years and another with their parents. Participants were recruited from the Teen Heart Study, which investigated EFs in children and adolescents with CHD at the University Children's Hospital Zurich. The Teen Heart Study included patients without genetic or dysmorphic syndromes who were born between 2004 and 2012 and underwent their first cardiopulmonary bypass (CPB) surgery before the age of 6 years [30]. Participants were asked what they consider the ideal content and scope of an intervention supporting EFs in children with CHD. These interviews were recorded with the consent of the participants and then transcribed and analyzed with structured qualitative content analysis [31]. This analysis produced nine categories, and all participants' suggestions and statements were assigned to one of these categories (table S1).

2.1.2 Delphi survey

The results of the focus group interviews were used to initiate a Delphi survey [32,33] by transforming the statements obtained from parents and children into a survey.

The Delphi survey was completed online by children with CHD (n = 7) aged 12 to 13 years who had not participated in the preceding focus group interviews, their teachers (n = 5), parents (n = 7), and health professionals (n = 5). These were all recruited from international hospitals and research institutions. The families were again recruited from the Teen Heart Study, but following focus group interview suggestions, only patients between 10 and 13 years were asked to participate. Hence, the Delphi survey was completed by four stakeholder groups: children with CHD, parents of children with CHD, teachers of children with CHD, and health professionals.

For survey one, respondents received a link by e-mail and were invited to rate each item on a Likert scale from 1 for "fully disagree" to 9 for "fully agree". They were encouraged to make further suggestions yet to be represented in the survey. Survey two was sent to all respondents of survey one. All items from survey one were included, and additional items suggested by participants were added following revision and categorization. The results of survey one were included in survey two: A table displayed the median and interquartile range (IQR) per stakeholder group for each item, and each participant was reminded of the scores they had given in survey one. Participants were asked to consider the input of all stakeholder groups in survey

For peer review only

one and then review their answers. Items reaching consensus (IQR <2) were dropped from the subsequent round. Survey three was sent to all respondents of survey two. Items for which no consensus was reached in survey two were included. No more items were added. Again, the results of survey two were included in survey three. Participants again reviewed their answers. At the end of survey three, every participant was asked about their interest in participating in the final consensus meeting.

Overall, participants completed three rounds with the goal of consensus-seeking across stakeholder groups, defined as IQR<2 for each item [34]. Items for which no consensus was reached after three rounds were discussed in a final consensus meeting with three parents, two health professionals, and the study team.

2.1.3 Final suggestions

The results of the Delphi survey showed that the wishes most strongly expressed by parents were that the intervention should include strategies supporting homework and everyday life activities and that the children should learn to develop their own strategies. Families participating in the Delphi survey wanted activities that did not resemble schoolwork, improved reasoning skills, and addressed academic future planning. Parents emphasized that the ideal age at which an EF intervention should start depends on the individual child, ranging from 4 to 18 (see 2_Appendix_Delphi, figure S1) Literature shows that EF development progresses considerably during childhood and adolescence [18]. This opens a window of opportunity for EF training during these periods [35,36]. Also, the families' perceived need for therapy is crucial for adherence [37]. During school age and adolescence, families become more aware of the sequelae of CHD and thus become more willing to participate in an intervention program. Participants also wanted minimal parental participation in the intervention, and the focus group interviews indicated that participants became aware of EF problems shortly before high school when remedial action might still be effective.

Consequently, we decided on an age range of 10 to 12 years instead of younger or older. No consensus was reached on whether the intervention should include personal meetings with the study team, whether the intervention should occur in a nearby community center, or on the design of the games (figure S2). The consensus meeting indicated that responses on these topics differed between children and families, and general agreement was difficult to reach. Hence, literature and practice in previous programs were used to decide on these elements [18,20,21,37–43]. For details, see 2_Appendix_Delphi.

2.2 Study design to test the feasibility of the intervention

This is a single-center, single-blinded randomized-controlled feasibility study to test the feasibility of the E-Fit intervention.

For peer review only

All participants will undergo a baseline neurodevelopmental assessment and be randomly assigned to either the E-Fit intervention or the control group. All participants will undergo a 2-month postintervention and a 6-month follow-up assessment. Investigators administering neuropsychological tests will be blinded to the participant's allocation. The families and the psychologist who conducts the intervention will be aware of the allocation (table S2).

2.3 Participants and recruitment

This study will include children with CHD who underwent CPB in Switzerland before one year of age. Diagnoses may include transposition of the great arteries, hypoplastic left heart syndrome, and other complex CHDs requiring CPB. At recruitment, children are 10-12 years old. Children diagnosed with a genetic or dysmorphic syndrome such as trisomy-21 or Ellis-van Creveld syndrome or with known large cerebral lesions or injuries, including stroke and severe hypoxic-ischemic encephalopathy with significant neurological sequelae preventing them from performing the assessments will be excluded.

Children will be recruited consecutively from the Department of Cardiology outpatient clinic at the University Children's Hospital Zurich. They will be identified through the clinical database comprising all treated children with CHD. All eligible patients and their parents will receive a letter with the study information and be asked to contact the study team if they are interested in participating. The study team will provide further information about the study and answer questions on the phone. After agreeing to participate and giving written informed consent (3_Appendix_

Form, parents and the child's principal teacher will fill in the Behavior Rating Inventory of Executive Functions (BRIEF) screening questionnaire. The BRIEF assesses EF behaviors in children and adolescents on three summary scales: a Behavior Regulation Index, a Metacognition Index, and a Global Executive Composite. Participants will be included in the study if a T-score ≥ 60 on any of the three summary scales is reported by either parents or teachers.

2.4 Patient involvement statement

As described above (see 2.1), patients, parents, and teachers were involved in the intervention development and design. Study results will be disseminated to participants and their families through a newsletter and a public informative meeting.

2.5 Randomization and stratification

After passing the screening questionnaire, participants will be randomly assigned to either the intervention or control group in a 1:1 design by block randomization (block size = 4, stratification variable: maternal education

For peer review only

level on a 6-point Likert scale). This list of random numbers will be generated prior to the start of the study in the statistical software R [44] and implemented in the randomization module on the research electronic database Redcap® [45], which allows allocation to be concealed until the participant is enrolled. At the end of the first assessment, after all tests and questionnaires are completed, the child will open an envelope prepared by a study member otherwise not involved in the study. The note in the envelope will reveal the allocation status to the family and baseline assessor.

2.6 Intervention group: E-Fit

The executive function intervention (E-Fit) targets four EF domains that have been identified as being impaired by two meta-analyses on EFs in children with CHD: inhibition, planning, flexibility, and working memory [4,5]. These are addressed in a multimodal approach consisting of three personalized and adaptive elements: 1) computerized training alone, 2) remote 1:1 strategy coaching with the child and a psychologist, and 3) analog board and card games fostering EF abilities that can be played alone or with friends and family (table S3). The intervention lasts for 2 months.

Computerized training

Participants use the platform CogniFit Inc © 2022 (CogniFit) for the computerized training. CogniFit was selected based on literature [46,47] and its successful use in other similarly aged populations [48] and because it targets several EF domains. This is in contrast to Cogmed, which only targets working memory. From the CogniFit platform, eleven games targeting EF domains were selected (table S4). These games are adaptive to the child's level of performance. Participants play the assigned games at home on a computer, smartphone, or tablet. A device is provided if needed. The child is asked to play three times a week for 20 min.

Strategy Coaching

The strategy coaching is conducted remotely. A psychologist is assigned to each family as a coach to monitor and support them throughout the intervention. The child meets with the coach once a week for 60 minutes for a total of eight sessions.

The first session discusses baseline assessment results and the child's strengths and weaknesses [49]. Further, the first module of CogniFit is performed to make sure the child understands how to use CogniFit. In this session, a parent participates with the child.

For the six following sessions, parents fill in a short questionnaire [39] to identify EF domains in which their child faces difficulties. Of eleven EF domains rated, the six indicating the most difficulties are selected for the coaching sessions. Each of these six sessions has standardized guidelines and starts with a story about an EF and an explanation of how this function engages our brain [40–43]. Afterwards, the child completes a task

For peer review only

teaching a strategy for the EF in question [50] (for more details, see 1_Appendix_further_material). During the coaching sessions, solutions to difficulties with adhering to E-Fit are suggested.

The eighth and final coaching session consists of the wrap-up and outlook, where a parent again participates with the child [51].

Additionally, the coach calls the parents in the first and fourth week to discuss the upcoming sessions that will be conducted with the child only. Parents can suggest specific situations to be addressed in the subsequent strategy coaching. In response, the coach provides tips on how parents can support their child with the respective EF [50]. For an overview of the strategy coaching procedure, see Table 1.

Following the manual, the strategy coaching teaches strategies to promote EFs in everyday life [50]. A clinical neuropsychologist reviewed the strategy coaching, and the manual is available in German language.

Analog training

Fourteen board and card games are provided to the families (table S5). They are suited for this age group and target EF abilities. A special needs educator, a trained game educator, helped select the games. Families are asked to report the date, duration, and name of the analog games played.

2.7 Control group

The control group receives standard of care, including cardiac surveillance, neurodevelopmental counseling, and therapies such as speech and physiotherapy. Because the control group families know the scope of the tested intervention, this may lead to contamination: during the trial, parents of control children may engage their children in activities used in the intervention fostering EF functions. To detect this and where occurring, to measure the time spent on it, parents and children complete an electronic diary four times a week for eight weeks to monitor the child's everyday activities (see 4_Appendix). After the final visit at the end of study participation, the control group families receive the list of EF-enhancing analog games used, EF fact sheets, and a 2-month CogniFit login.

2.8 Primary outcome measures

The feasibility of the E-Fit intervention will be measured in six areas, as described by Bowen et al. [52]. Except for Practicality and Limited efficacy, primary outcome measures will be collected only in the intervention group throughout the intervention period. For a summary of measures and assessment timepoints see Table 2. The following areas will be assessed.

Acceptability

For peer review only

Acceptability examines suitability, satisfaction, and attractiveness to program deliverers and recipients. It is measured by questions about satisfaction, intent to continue use, and perceived appropriateness.

Demand

Demand is assessed by the extent to which the intervention is likely to be used or how much demand exists. This includes measures for the fit within the family, perceived positive or negative effects on the family, actual use, expressed interest or intention to use, and perceived demand.

Implementation

Implementation is concerned with how providers can successfully deliver the intervention to intended participants. Implementation is measured by the success and degree of delivery. For this purpose, the E-Fit Fidelity Measurement System was newly developed according to existing guidelines [53].

Practicality

Practicality explores how an intervention can be delivered to intended participants with existing means, resources, and circumstances. It includes factors affecting implementation ease or difficulty, efficiency, speed and quality of implementation, positive or negative effects on target participants, and the participant's ability to complete the intervention activities.

Integration

Integration assesses the extent to which the intervention can be integrated within the existing system. This addresses perceived fit with infrastructure and perceived sustainability.

Limited efficacy

Limited efficacy explores whether the new intervention shows promise of succeeding with the intended population. This is measured by evaluating the program's intended effects or process on key intermediate variables, effect-size estimation, and maintenance of changes from initial change (See Table 3 for an overview of EF measures).

For peer review only

2.9 Secondary outcome measures

In addition to primary outcome measures, we will examine other variables assessing the success of the intervention. The following secondary outcome measures are to be collected from both groups at baseline, 2-month, and 6-month follow-up: The Family Relationship Index is a self-report measure used to evaluate subjective quality of family environment for parents [54]. The Kidscreen-10 assesses the child's subjective health and well-being, and the Kidscreen-27 assesses the child's health and well-being from a parent's perspective; these are combined to assess the child's QoL [55]. The parents will complete the short form of the Conners-3 and the Social Responsiveness Scale [56,57] to screen for behavioral problems. The Resilience

Scale (RS-13) is a self-reported questionnaire assessing resilience by acceptance of self-, life- and personal competencies. This questionnaire will be completed by the parents and the child [58].

2.10 Covariate measures

Medical, sociodemographic, and patient-specific factors, and IQ will be measured in both groups at baseline and be treated as covariates for the effectiveness of the intervention and to describe the population who participated in the intervention.

Socioeconomic status (SES) will be estimated on a six-point scale for both paternal and maternal education; leading to the lowest possible SES sum score of 2, and the highest of 12 [59]. Cardiac and neonatal data will be collected from medical records. A short form of the Wechsler Intelligence Scale for Children, fifth edition will be used to assess total IQ once at the baseline assessment [60].

2.11 Determination of sample size

This is a proof-of-concept study to test the feasibility of a new intervention. Accordingly, the primary outcome is feasibility, thus no minimum sample size has been calculated [61,62]. A retrospective power analysis will be conducted. Instead, the sample size was defined from previous literature [62,63]. Using a pilot study with a sample size <30 may lead to the potential to underrecruit for the main study [62]. To provide a high level of confidence, a total sample size between 35 and 55 is suggested to account also for small effect sizes. As the total population to recruit from for the present study is relatively small, we decided on a sample size of 40 (E-Fit intervention group n = 20, control group n = 20).

Approximately 210 children are eligible for screening and are to be contacted. In a study by Spillmann et al. [15] using a cut-off of $\geq$ 65 in the BRIEF, around 20% of children with complex CHD had EF difficulties at 10 years. Using data from children and adolescents between 10 and 15 years and a cut-off of $\geq$ 60, we estimate around 30% of eligible children to demonstrate EF difficulties, leading to a potential sample size of 60 children. We assume a response rate of around 75% from those families with children with EF problems, leading to a study sample of approximately 45 patients. A drop-out rate of approximately 10% allows the planned sample of 40 participants to be achieved.

2.12 Data analysis plan

The feasibility of the intervention will be delineated from the descriptive statistics.

Descriptive statistics will be calculated, including means, SD, medians, and IQRs for continuous variables and frequency counts and percentages for categorical variables. The newly developed E-Fit Fidelity Measurement System will be evaluated. Two raters will complete it individually, and inter-rater reliability will be reported. To

For peer review only

report limited efficacy, within-subject differences between EF performances from baseline to post-treatment and 6-month follow-up assessments are to be investigated across treatment groups with an intention-to-treat analysis. Linear regression controlling for covariates, involving differences in means and 95% CI, will be used to assess differences between groups and timepoints for continuous outcomes. Proportions and logistic regressions for binary outcomes will be used to examine group effects (e.g., comparison of clinically relevant vs. not clinically relevant scores in the BRIEF, $\chi 2$ tests, ORs, and 95% CI). Given that missing values are missing at random, they will be imputed using multiple imputations.

The data will be visually inspected to identify discordant and influential observations. All analyses will be conducted with an alpha level of 0.05. The statistical software R will be used for all analyses [64]. The final project report will describe and justify any deviations from the original statistical plan.

3. Ethics and dissemination

The protocol was approved by the ethical committee of the Canton of Zurich in Switzerland (BASEC-Nr: 2021-02413) and was registered on ClinicalTrials.gov (NCT05198583). Written informed consent will be obtained from the parents. Data handling, record keeping, and archiving will be performed according to the guidelines given by the ethical committee. The results of this study will be disseminated through presentations at national and international conferences, publications in peer-reviewed journals, and directly to families who participated in the study.

Key outputs from the trial will contribute to our dissemination and impact agenda: Pilot data will inform the decision whether to proceed to a definitive randomized controlled trial and if we do, we will have evidence to support funding applications and a finalized intervention delivery manual to enable multicenter replication of E-Fit.

4. Acknowledgments

The authors thank Nina Zeltner, who supported the qualitative analysis, and Thomas Pletschko, who reviewed the structure and content of the strategy coaching.

Word count main document: 3958

Funding, roles and responsibilities

For peer review only

This work was supported by the Swiss National Science Foundation (SNF) grant number 32003B_172914. This funding source had no role in the design of this study and will not have any role during its execution, analyses, interpretation of data, or decision to submit results.

CogniFit Inc © 2022 had no role in the design of this study and will not have any role during its execution, analyses, interpretation of data, or decision to submit results.

Authors contributions

BL, ME, FW, RO'GT, OK, ML, and AS conceived and designed the study. *BL* is the grant holder. *AS* wrote and revised the manuscript. All authors critically revised the manuscript for important intellectual content.

For peer review only

Table 1. Strategy coaching procedure.

	Strategy coaching procedure							
	Week 1	***Week 2***	***Week 3***	***Week 4***	***Week 5***	***Week 6***	***Week 7***	***Week 8***
Child		60min individual topic	60min individual topic	60min individual topic	60min individual topic	60min individual topic	60min individual topic	
Parent	30min Call			30min Call				
Parent and Child	60min initialization session							45min Wrap up session

For peer review only

Table 2 Feasibility focus areas and assessment tools

Area of focus	Assessment tool	Group	Timepoint	Completed by
Acceptability	Acceptance and Feasibility scale [65]	E-Fit	Final coaching	Parents and children
	Children's Feeling scale [65]	E-Fit	Intervention period, before and after each online training	Children
	Observed engagement [66]	E-Fit	Each coaching session	Coach
Demand	Acceptance and Feasibility scale	E-Fit	Final coaching	Parents and children
	Fun and Demand [67]	E-Fit	Intervention period, before and after each online training	Children
	Session data: duration, number of completed sessions, and for the computerized training, the coaching, and the card games	E-Fit	Intervention period	
	Recruitment rate	E-Fit	End of study	
Implementation	E-Fit Fidelity Measurement System tool, developed based on Feely et. al [53]	E-Fit	End of study	Two separate raters
Practicality	Acceptance and Feasibility scale [65]	E-Fit	Final coaching	Parents and children
	Feedback in coaching sessions	E-Fit	Each coaching session	Parents and children
	Observed involvement [66]	E-Fit	End of study	
	Session rating scale [68]	E-Fit	Each coaching session	Children
	Observed engagement [66]	E-Fit	Each coaching session	Coaches
	Recruitment rates	Both	End of study	
	Retention rates	Both	End of study	
	Completion of weekly sessions	E-Fit	End of study	
	f events (AEs)	Both	Continuously	
Integration	Acceptance and Feasibility scale	E-Fit	Final coaching	Parents and children
Limited efficacy	Delis-Kaplan Executive Function System [69]	Both	BL, T_1, T_2	
	Wechsler Intelligence Scale for Children- 5th edition [60]	Both	BL, T_1, T_2	
	Test of Attentional Performance [70]	Both	BL, T_1, T_2	
	Balloon Analog Risk Task [71]	Both	BL, T_1, T_2	
	Behavior Rating Inventory for Executive Function [72]	Both	BL, T_1, T_2	Parents* and teachers

*Note: *if possible, completed by both parents separately*

For peer review only

Table 3 Executive function domains

EF domain	Assessment test
Inhibition	Color Word Interference Task (D-KEFS)<br>Go/No-Go (TAP)
Cognitive Flexibility	Trail-making Test (D-KEFS)<br>Color Word Interference Task (D-KEFS)
Planning	Tower Test (D-KEFS)
Working Memory	Digit Span (WISC-V)<br>Picture Span (WISC-V)<br>Number Letter Sequencing (WISC-V)
Risk-taking	Balloon Analog Risk Task

Abbreviations: D-KEFS, Delis-Kaplan Executive Function System; EF, executive function; WISC -V, Wechsler Intelligence Scale for Children fifth edition

For peer review only

References

1 Bernier P-L, Stefanescu A, Samoukovic G, *et al.* The Challenge of Congenital Heart Disease Worldwide: Epidemiologic and Demographic Facts. *Semin Thorac Cardiovasc Surg Pediatr Card Surg Annu* 2010;**13**:26–34. doi:10.1053/j.pcsu.2010.02.005

2 Wernovsky G, Licht DJ. Neurodevelopmental Outcomes in Children with Congenital Heart Disease – What can we impact? *Pediatr Crit Care Med J Soc Crit Care Med World Fed Pediatr Intensive Crit Care Soc* 2016;**17**:S232–42. doi:10.1097/PCC.0000000000000800

3 Marino BS, Lipkin PH, Newburger JW, *et al.* Neurodevelopmental Outcomes in Children With Congenital Heart Disease: Evaluation and Management. *Circulation* 2012;**126**:1143–72. doi:10.1161/CIR.0b013e318265ee8a

4 Feldmann M, Bataillard C, Ehrler M, *et al.* Cognitive and Executive Function in Congenital Heart Disease: A Meta-analysis. *Pediatrics* 2021;**148**:e2021050875. doi:10.1542/peds.2021-050875

5 Jackson WM, Davis N, Calderon J, *et al.* Executive functions in children with heart disease: a systematic review and meta-analysis. *Cardiol Young* 2021;**31**:1914–22. doi:10.1017/S1047951121001074

6 Diamond A. Executive Functions. *Annu Rev Psychol* 2013;**64**:135–68. doi:10.1146/annurev-psych-113011-143750

7 Lehto JE, Juujärvi P, Kooistra L, *et al.* Dimensions of executive functioning: Evidence from children. *Br J Dev Psychol* 2003;**21**:59–80. doi:10.1348/026151003321164627

8 Miyake A, Friedman NP, Emerson MJ, *et al.* The Unity and Diversity of Executive Functions and Their Contributions to Complex “Frontal Lobe” Tasks: A Latent Variable Analysis. *Cognit Psychol* 2000;**41**:49–100. doi:10.1006/cogp.1999.0734

9 Miyake A, Friedman NP. The Nature and Organization of Individual Differences in Executive Functions: Four General Conclusions. *Curr Dir Psychol Sci* 2012;**21**:8–14. doi:10.1177/0963721411429458

10 Baggetta P, Alexander PA. Conceptualization and Operationalization of Executive Function. *Mind Brain Educ* 2016;**10**:10–33. doi:10.1111/mbe.12100

11 Blair C, Razza RP. Relating Effortful Control, Executive Function, and False Belief Understanding to Emerging Math and Literacy Ability in Kindergarten. *Child Dev* 2007;**78**:647–63. doi:10.1111/j.1467-8624.2007.01019.x

12 Miller M, Nevado-Montenegro AJ, Hinshaw SP. Childhood Executive Function Continues to Predict Outcomes in Young Adult Females with and Without Childhood-Diagnosed ADHD. *J Abnorm Child Psychol* 2012;**40**:657–68. doi:10.1007/s10802-011-9599-y

13 Taylor Tavares JV, Clark L, Cannon DM, *et al.* Distinct Profiles of Neurocognitive Function in Unmedicated Unipolar Depression and Bipolar II Depression. *Biol Psychiatry* 2007;**62**:917–24. doi:10.1016/j.biopsych.2007.05.034

14 Baler RD, Volkow ND. Drug addiction: the neurobiology of disrupted self-control. *Trends Mol Med* 2006;**12**:559–66. doi:10.1016/j.molmed.2006.10.005

15 Spillmann R, Polentarutti S, Ehrler M, *et al.* Congenital heart disease in school-aged children: Cognition, education, and participation in leisure activities. *Pediatr Res* 2021;:1–7. doi:10.1038/s41390-021-01853-4

16 Sanz JH, Wang J, Berl MM, *et al.* Executive Function and Psychosocial Quality of Life in School Age Children with Congenital Heart Disease. *J Pediatr* 2018;**202**:63–9. doi:10.1016/j.jpeds.2018.07.018

17 Jackson JL, Gerardo GM, Monti JD, *et al.* Executive Function and Internalizing Symptoms in Adolescents and Young Adults With Congenital Heart Disease: The Role of Coping. *J Pediatr Psychol* 2018;**43**:906–15. doi:10.1093/jpepsy/jsx154

For peer review only

For peer review only - http://bmjopen.bmj.com/site/about/guidelines.xhtml

18 Calderon J, Bellinger DC. Executive function deficits in congenital heart disease: why is intervention important? *Cardiol Young* 2015;**25**:1238–46. doi:10.1017/S1047951115001134

19 Diamond A. Why improving and assessing executive functions early in life is critical. In: *Executive function in preschool-age children: Integrating measurement, neurodevelopment, and translational research*. Washington, DC, US: : American Psychological Association 2016. 11–43. doi:10.1037/14797-002

20 Calderon J, Wypij D, Rofeberg V, *et al.* Randomized Controlled Trial of Working Memory Intervention in Congenital Heart Disease. *J Pediatr* 2020;**227**:191-198.e3. doi:10.1016/j.jpeds.2020.08.038

21 Jordan LC, Siciliano RE, Cole DA, *et al.* Cognitive training in children with hypoplastic left heart syndrome: A pilot randomized trial. *Prog Pediatr Cardiol* 2020;**57**:101185. doi:10.1016/j.ppedcard.2019.101185

22 Cogmed. https://www.cogmed.com/training (accessed 19 Jan 2023).

23 Takacs ZK, Kassai R. The efficacy of different interventions to foster children's executive function skills: A series of meta-analyses. *Psychol Bull* 20190429;**145**:653. doi:10.1037/bul0000195

24 Diamond A, Lee K. Interventions Shown to Aid Executive Function Development in Children 4 to 12 Years Old. *Science* Published Online First: 19 August 2011. doi:10.1126/science.1204529

25 Flook L, Smalley SL, Kitil MJ, *et al.* Effects of Mindful Awareness Practices on Executive Functions in Elementary School Children. *J Appl Sch Psychol* 2010;**26**:70–95. doi:10.1080/15377900903379125

26 Schonert-Reichl K, Lawlor M. The Effects of a Mindfulness-Based Education Program on Pre- and Early Adolescents' Well-Being and Social and Emotional Competence. *Mindfulness* 2010;**1**:137–51. doi:10.1007/s12671-010-0011-8

27 Modi AC, Mara CA, Schmidt M, *et al.* Epilepsy Journey: A proof of concept trial of a Web-based executive functioning intervention for adolescents with epilepsy. *Epilepsia* 2019;**60**:1895–907. doi:10.1111/epi.16317

28 Gonçalves PD, Ometto M, Sendoya G, *et al.* Neuropsychological Rehabilitation of Executive Functions: Challenges and Perspectives. *J Behav Brain Sci* 2014;**2014**. doi:10.4236/jbbs.2014.41004

29 Mackey AP, Hill SS, Stone SI, *et al.* Differential effects of reasoning and speed training in children. *Dev Sci* 2011;**14**:582–90. doi:10.1111/j.1467-7687.2010.01005.x

30 Ehrler M, Naef N, Tuura RO, *et al.* Executive function and brain development in adolescents with severe congenital heart disease (Teen Heart Study): protocol of a prospective cohort study. *BMJ Open* 2019;**9**:e032363. doi:10.1136/bmjopen-2019-032363

31 Kukartz U. *Qualitative Inhaltsanalyse. Methoden, Praxis, Computerunterstützung*. 4. überarbeitete Auflage. 2018.

32 Gordon T, Helmer O. REPORT ON A LONG-RANGE FORECASTING STUDY. 1964. https://apps.dtic.mil/sti/citations/AD0607777 (accessed 23 Jan 2023).

33 Hasson F, Keeney S, McKenna H. Research guidelines for the Delphi survey technique. 2000;**32**:1008–15.

34 Scheibe M, Skutsch M, Schofer J. *The Delphi Method: Techniques and Applications*. London Reading, Mass.: Addison-Wesley 1975.

35 Drzewiecki CM, Juraska JM. The structural reorganization of the prefrontal cortex during adolescence as a framework for vulnerability to the environment. *Pharmacol Biochem Behav* 2020;**199**:173044. doi:10.1016/j.pbb.2020.173044

For peer review only - http://bmjopen.bmj.com/site/about/guidelines.xhtml

36 Teffer K, Semendeferi K. Chapter 9 - Human prefrontal cortex: Evolution, development, and pathology. In: Hofman MA, Falk D, eds. *Progress in Brain Research*. Elsevier 2012. 191–218. doi:10.1016/B978-0-444-53860-4.00009-X

37 Long SH, Eldridge BJ, Harris SR, *et al.* Challenges in Trying to Implement an Early Intervention Program for Infants with Congenital Heart Disease. *Pediatr Phys Ther* 2015;**27**:38–43. doi:10.1097/PEP.0000000000000101

38 Cognifit. Cognifit Inc © 2022. 2022.

39 Dawson P, Guare R. *Schlau aber...* 1st ed. Bern: : Verlag Hans Huber, Hogrefe AG 2012.

40 Liebers A, Kubesch S. *Ein Ritter in der Klasse*. 1st ed. Verlag Bildung Plus 2013.

41 Liebers A, Kubesch S. *Mathetest und Drachenhörnchen*. 1st ed. Verlag Bildung Plus 2013.

42 Liebers A, Kubesch S. *Ob Gespenter Fußball spielen?* 1st ed. Verlag Bildung Plus 2014.

43 Liebers A, Kubesch S. *Stopp oder es kracht!* Verlag Bildung Plus 2014.

44 R Core Team. R: A language and environment for statistical computing. Vienna, Austria: 2020. https://www.R-project.org/

45 Wright A. REDCap: A Tool for the Electronic Capture of Research Data. *J Electron Resour Med Libr* 2016;**13**:197–201. doi:10.1080/15424065.2016.1259026

46 Bustamante JC, Navarro J-J. Technological Tools for the Intervention and Computerized Dynamic Assessment of Executive Functions. Handb. Res. Neurocognitive Dev. Exec. Funct. Implic. Interv. 2022;:310–39. doi:10.4018/978-1-7998-9075-1.ch014

47 Rachanioti E, Bratitsis T, Alevriadou A. Cognitive games for children's Executive Functions Training with or without learning difficulties: an Overview. In: *Proceedings of the 8th International Conference on Software Development and Technologies for Enhancing Accessibility and Fighting Info-exclusion*. Thessaloniki Greece: : ACM 2018. 165–71. doi:10.1145/3218585.3218665

48 Horowitz-Kraus T. Differential Effect of Cognitive Training on Executive Functions and Reading Abilities in Children With ADHD and in Children With ADHD Comorbid With Reading Difficulties. *J Atten Disord* 2015;**19**:515–26. doi:10.1177/1087054713502079

49 Leiss U. Die neuropsychologische Ergebnisbesprechung als Intervention – Eine Brücke zwischen Befund und Alltag. In: Pletschko T, Leiss U, Pal-Handl K, *et al.*, eds. *Neuropsychologische Therapie mit Kindern und Jugendlichen: Praktische Behandlungskonzepte bei neurokognitiven Funktionsstörungen*. Berlin, Heidelberg: : Springer 2020. 35–43. doi:10.1007/978-3-662-59288-5_4

50 Dawson P, Guare R. *Smart But Scattered: The Revolutionary "executive Skills" Approach to Helping Kids Reach Their Potential*. Guilford Press 2009.

51 Pletschko T, Leiss U, Pal-Handl K, *et al. Neuropsychologische Therapie mit Kindern und Jugendlichen: Praktische Behandlungskonzepte bei neurokognitiven Funktionsstörungen*. Springer-Verlag 2020.

52 Bowen DJ, Kreuter M, Spring B, *et al.* How We Design Feasibility Studies. *Am J Prev Med* 2009;**36**:452–7. doi:10.1016/j.amepre.2009.02.002

53 Feely M, Seay KD, Lanier P, *et al.* Measuring Fidelity in Research Studies: A Field Guide to Developing a Comprehensive Fidelity Measurement System. *Child Adolesc Soc Work J* 2018;**35**:139–52. doi:10.1007/s10560-017-0512-6

54 Hoge RD, Andrews DA, Faulkner P, *et al.* The family relationship index: Validity data. *J Clin Psychol* 1989;**45**:897–903. doi:10.1002/1097-4679(198911)45:6<897::AID-JCLP2270450611>3.0.CO;2-T

For peer review only

For peer review only - http://bmjopen.bmj.com/site/about/guidelines.xhtml

55 Ravens-Sieberer U. The KIDSCREEN questionnaires. Quality of life quiestionners for children and adolescents - handbook. 2006.

56 Lidzba K, Chrstiansen H, Drechsler R. Conners Skalen zu Aufmerksamkeit und Verhalten - 3. Deutschsprachige Adaptation der Conners 3rd Edition® (Conners 3®) von C. Keith Conners. 2013.

57 Bölte S, Poustka F. Skala zur Erfassung sozialer Reaktivität - Dimensionale Autismus-Diagnostik. Deutsche Fassung der Scoial Responsiveness Scale (SRS) von John N. Constantino und Christian P. Gruber. 2007.

58 Leppert K, Koch B, Brähler E, *et al.* Resilience scale - evaluation of a long (RS-25) and a short version (RS-13). *Klin Diagn Eval* 2008.

59 Largo RH, Pfister D, Molinari L, *et al.* Significance of prenatal, perinatal and postnatal factors in the development of AGA preterm infants at five to seven years. *Dev Med Child Neurol* 1989;**31**:440–56.

60 Wechsler D. Study Citation Wechsler, D. (2014). WISC-V: Technical and Interpretive Manual. Bloomington. 2014.

61 Eldridge SM, Lancaster GA, Campbell MJ, *et al.* Defining Feasibility and Pilot Studies in Preparation for Randomised Controlled Trials: Development of a Conceptual Framework. *PLOS ONE* 2016;**11**:e0150205. doi:10.1371/journal.pone.0150205

62 Sim J, Lewis M. The size of a pilot study for a clinical trial should be calculated in relation to considerations of precision and efficiency. *J Clin Epidemiol* 2012;**65**:301–8. doi:10.1016/j.jclinepi.2011.07.011

63 Billingham SA, Whitehead AL, Julious SA. An audit of sample sizes for pilot and feasibility trials being undertaken in the United Kingdom registered in the United Kingdom Clinical Research Network database. *BMC Med Res Methodol* 2013;**13**:104. doi:10.1186/1471-2288-13-104

64 RStudio Team. RStudio: Integrated Development Environment for R. 2021.http://www.rstudio.com/

For peer review only

For peer review only - http://bmjopen.bmj.com/site/about/guidelines.xhtml

A) Computerized training (CogniFit Inc © 2022)

Game	Trained skills			
	flexibility	working memory	planning	inhibition
Traffic Manager			x	
Water Lilies		x	x	
Reaction Field	x			x
Happy Hopper				x
Neuron Madness				x
Minus Malus	x	x		
Tennis Bowling	x			
Candy Line Up		x	x	
Bee Balloon	x			
Slice and Drop			x	
Cube Foundry			x	

Note: x *= primarily trained functions,* x *= additionally trained functions.*

It is important to note that a contract was concluded with CogniFit Inc © 2022, because they provide discounts on their services in exchange for the pseudonymized baseline assessment data. Families had the choice whether to provide their data or not.

B) Strategy coaching

At the beginning of the intervention, parents complete a short questionnaire [1], assessing the behavior of their child in everyday life. The questionnaire was designed for parents to identify EF domains in which their children face difficulties. Lower scores indicate greater difficulties. Out of eleven EF domains rated, the six lowest scored are selected for the coaching sessions. In the first coaching session, the child's performance in the EF baseline assessment are discussed, and strengths and difficulties are identified [2]. Each of the next six sessions focuses on one of the six EF domains identified as most affected [1]. At the beginning of each session, the previous week is reviewed, focusing on the EF intervention and coaching instructions given at the previous session. Also, difficulties in regard to adherence are discussed and solutions suggested by the coach. In the second part of the session, a short story is read, featuring protagonists dealing with similar challenge as is the topic of that coaching session [3–6]. In the third part of the session, the focus is transferred to the child's situation by asking about how they could handle similar situations. Further, the child completes a task for the coaching session. Templates for these are available in Dawson et al.'s book *Smart but scattered* [1] and include checklist, plans, and tables. At the end of the session, the child is encouraged to use these strategies the following week, and the parents are informed by email about these strategies after the session. The parents receive fact sheets regarding the discussed EF domain of that particular session with tips for everyday life and a short summary on what was done in the session. All parental tips are also taken from the German version of *Smart but scattered* [1].

For peer review only - http://bmjopen.bmj.com/site/about/guidelines.xhtml

C) Analog training

Game	Trained skills			
	flexibility	working memory	planning	inhibition
Gold (Game Factory ®)	x	x	x	
Dodelido (“Drei Magier ®”)	x	x		x
Särge schubsen (“Drei Magier ®”)	x	x	x	x
Geistesblitz 2.0 (“Zoch”)	x	x		x
Beaver Gang (“Amigo Spiele”)	x	x	x	
Beaver Clan (“Amigo Spiele”)	x	x	x	
Memo Dice (“Amigo Spiele”)	x	x	x	
5er finden (HABA ®)	x		x	
The nasty 7 (“Drei Hasen in der Abendsonne”)	x	x		x
Yokai (Game Factory ®)	x	x	x	
Okiya (“Pegasus Spiele”)	x		x	
Perplexus Crazy run (2 PE Original, 5 FT 208, 6 PE Epic)				x
Thunder Pops				x

Games selected based on https://www.spielendfoerdern.ch/assets/spielliste_1.5.pdf

For peer review only

1 Dawson P, Guare R. *Schlau aber...* 1st ed. Bern: : Verlag Hans Huber, Hogrefe AG 2012.
2 Leiss U. Die neuropsychologische Ergebnisbesprechung als Intervention – Eine Brücke zwischen Befund und Alltag. In: Pletschko T, Leiss U, Pal-Handl K, *et al.*, eds. *Neuropsychologische Therapie mit Kindern und Jugendlichen: Praktische Behandlungskonzepte bei neurokognitiven Funktionsstörungen*. Berlin, Heidelberg: : Springer 2020. 35–43. doi:10.1007/978-3-662-59288-5_4
3 Liebers A, Kubesch S. *Ob Gespenter Fußball spielen?* 1st ed. Verlag Bildung Plus 2014.
4 Liebers A, Kubesch S. *Mathetest und Drachenhörnchen*. 1st ed. Verlag Bildung Plus 2013.
5 Liebers A, Kubesch S. *Ein Ritter in der Klasse*. 1st ed. Verlag Bildung Plus 2013.
6 Liebers A, Kubesch S. *Stopp oder es kracht!* Verlag Bildung Plus 2014.

For peer review only

A) Development of the Delphi survey

Sentences derived by qualitative content analysis from the focus groups were sorted into nine categories. Then, the statements were summarized and finally used to define the items in the Delphi survey. An example of statements and the consequent items is given in table S6. The items are deliberately positively formulated. Some items were highly specific to children with CHD. Hence, the items for the “identity” and “dealing with the disease” categories were dropped for the Delphi survey. The final set of items included in the Delphi survey is provided in figure S1.

B) Rationale behind the final intervention design

B.1) Age

The age range resulting from the focus groups was between 10 and 12 years of age. In the Delphi survey, participants were quite discordant. The consensus meeting was unable to define an ideal age, and it was felt that the age for an intervention was very child specific. The literature shows that EF development progresses considerably during childhood and adolescence [1]. Brain structures associated with EFs still mature during adolescence and early adulthood [2]. This opens a window of opportunity for EF training during these periods [3]. In addition, the families’ perceived need for therapy is crucial for adherence [4]. During school-age and adolescence, families become more aware of the sequelae of the CHD and thus are willing to participate in an intervention program. The participants also wanted minimal parental participation in the intervention, and the focus group discussion indicated that participants became aware of EF problems shortly before high school, when remedial action might still be effective. Consequently, we decided on an age range of 10 to 12 years instead of younger or older [5–7].

B.2) Virtual and analog games

Another element key to the success of any intervention is motivation [8]. Johann and Karbach compared six computer-based cognitive training programs [9]. They found evidence that playful interventions promote motivation and adherence in typically developing elementary school students. This reinforced the effects achieved by the EF training [9]. In addition, they emphasize the difference between computer-based training with added game elements and regular video games [10]: Videogames such as Super Mario and Fruit Ninja have no effect on EFs. In contrast, computer-based training with added game elements, such as in Lumosity® or CogniFit Inc © 2022 [11,12] shows clear benefits for EF improvement. In computer-based approaches, the training effect is maintained, and motivation is increased [10]. Because an intervention should be enjoyable to keep participants motivated, analog games such as board and card games have also been tested as cognitive training [13,14]. One obstacle associated with interventions using analog games is the mandatory involvement

For peer review only - http://bmjopen.bmj.com/site/about/guidelines.xhtml

of other family or study members, which might overstrain time resources. However, participants of both the focus group and the Delphi survey indicated that it would be important to include analog games in the intervention. Hence, different analog games were added to the intervention, including games that can be played alone, and this part was decided to be open in terms of frequency and time spent on it. Moreover, whether the intervention should be online or analog was discussed without reaching a consensus by the Delphi survey participants. A systematic review and meta-analysis by Cao et al. found slight-to-moderate evidence for the effectiveness of computer-based interventions in promoting EF [10]. Computer-based interventions have been used in the population of CHD patients previously, and in the Delphi survey, only 4 of 12 participants disagreed with the online format. Hence, we decided that the intervention should include computer-based games. In addition, participating children could also use analog games. We decided to use existing computer-based and analog games instead of creating new games.

The intervention also includes analog and computerized games which, in addition to EF, promote eye-hand coordination, thus meeting a suggestion that motor games be included.

B.3) Strategy Coaching

The most highly rated items were related to learning strategies addressing everyday lives and homework. This is in line with the literature: another substantial shortcoming of many interventional studies is the lack of transfer [7,15–17]. Transfer refers to the degree of improvement in specific tasks requiring the same cognitive functions, termed near transfer, and in general cognitive abilities, termed far transfer [18]. A way to overcome a lack of transfer is by providing personal coaching sessions that support EF strategies in daily life and take into account children's emotional and social development [7]. Hence, we decided on adding strategy coaching. Career planning was a topic discussed in the Delphi survey. It was decided not to include this topic, as this is not appropriate for an age group of 10–12-year-old children.

B.4) Length, frequency per week and overall duration of the intervention

The most difficult aspect of the development of the intervention related to the minutes per day, frequency per week, and overall duration of the sessions. A wide range of possibilities was suggested (see figure S2). These could not all be met. The length of the computerized training sessions was defined as 20 minutes, which was in agreement with the participants' suggestions. Other studies using session lengths of 20 minutes trained three to four times a week [19,20], which was a higher frequency than suggested by the families. Consequently, we decided on a frequency of three times a week and an overall duration of 8 weeks instead of the 6 months suggested (figure S2).

For peer review only - http://bmjopen.bmj.com/site/about/guidelines.xhtml

Table S6 Examples of item construction

Categories	Examples	Summary	Item
1 Identity/EF	*"But what I would certainly like for [my son] is … that he is trained a bit. … What should [he] do first, or how can [he] plan the day."*	Daily planning as an everyday problem.	Children with congenital heart defects structure their everyday life
2 Identity/Relation to peers	*"he needs more time and he's a bit slower and then he sees [his] siblings [and] that they're always faster, and then he always makes himself small and that's a shame."*	Is slower than Peers which affects him negatively.	Children with congenital heart defects feel equal compared to their siblings/peers
3 Scope of intervention/game suggestions	*"analog games would also be OK, [I]can imagine that."*	Analog games.	Dice games should be part of such an intervention.
4 Scope of intervention/structure	*"we would like it if it were possible online, because travelling … is always- … a bit far, yes. Especially during school time"*	Online is better for families who live further away from the hospital.	The intervention should be conducted online
5 Scope of intervention/possible distracting factors	*"they just have … a lot of homework …, then he plays the cello, then he goes to the youth club, everything interests him."*	Other hobbies also need a lot of time and should still have room.	Hobbies could get in the way of the intervention
6 Scope of intervention/possible supporting factors	*"That it [should not be like] 'oh that in addition,' but 'great, … that would be great right now and is manageable in terms of time'."*	Time-manageable and fun.	This is the maximum amount of time per day the participants should work on the intervention (in free time):
7 Dealing with the disease/challenges	*"[that] is … something that … she finds quite difficult and where she probably swallows a lot."*	She finds it difficult to talk about her illness and swallows a lot.	Children with congenital heart defects talk about their heart defect
8 Dealing with the disease/supporting factors	*"contact with other heart children, is certainly motivating."*	Contact with other heart children can be motivating.	During the intervention, the children and adolescents should have contact with other children and adolescents
9 Further ideas	*"I think [contact with other children] will motivate them more than if they do their exercise in their corner [or] maybe talk to someone from your team from time to time"*	Contact with other children with CHD or with the study team	In such an intervention, there should be face-to-face meetings with the study team

For peer review only - http://bmjopen.bmj.com/site/about/guidelines.xhtml

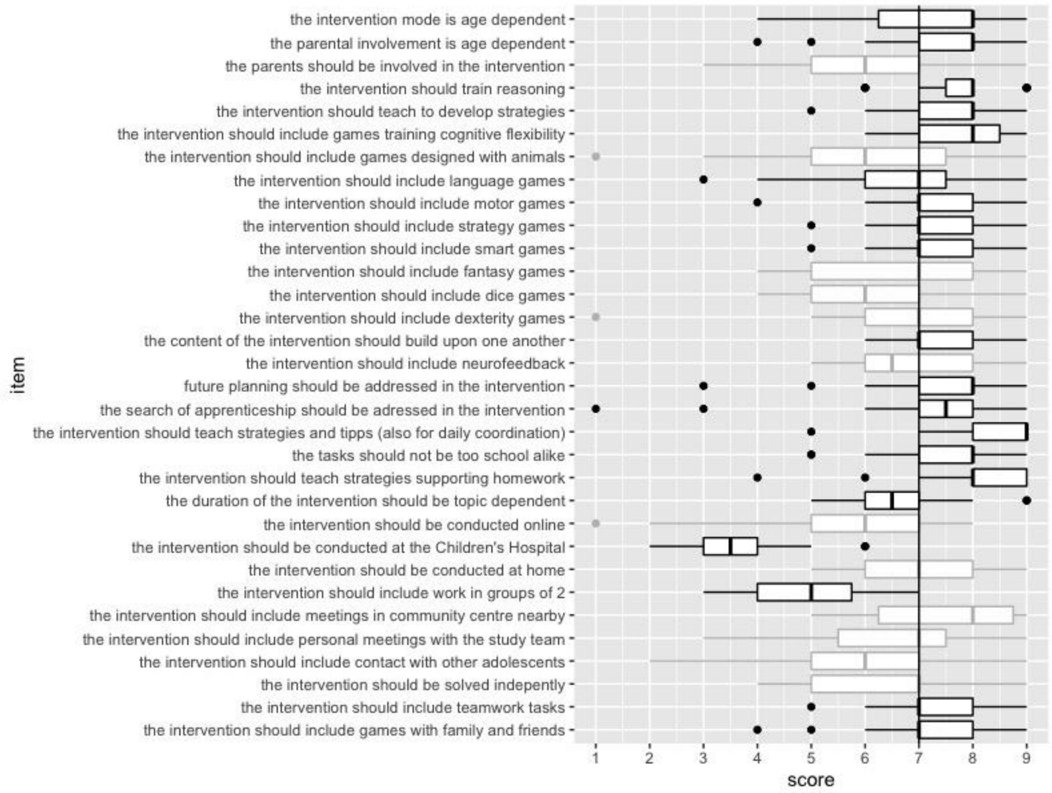

Figure S1. Ratings on Delphi items. 1 = fully disagree, 9 = fully agree. With a median of 7 and an IQR < 2 the item was considered accepted by the participants. Items in gray are the items for which no consensus was found.

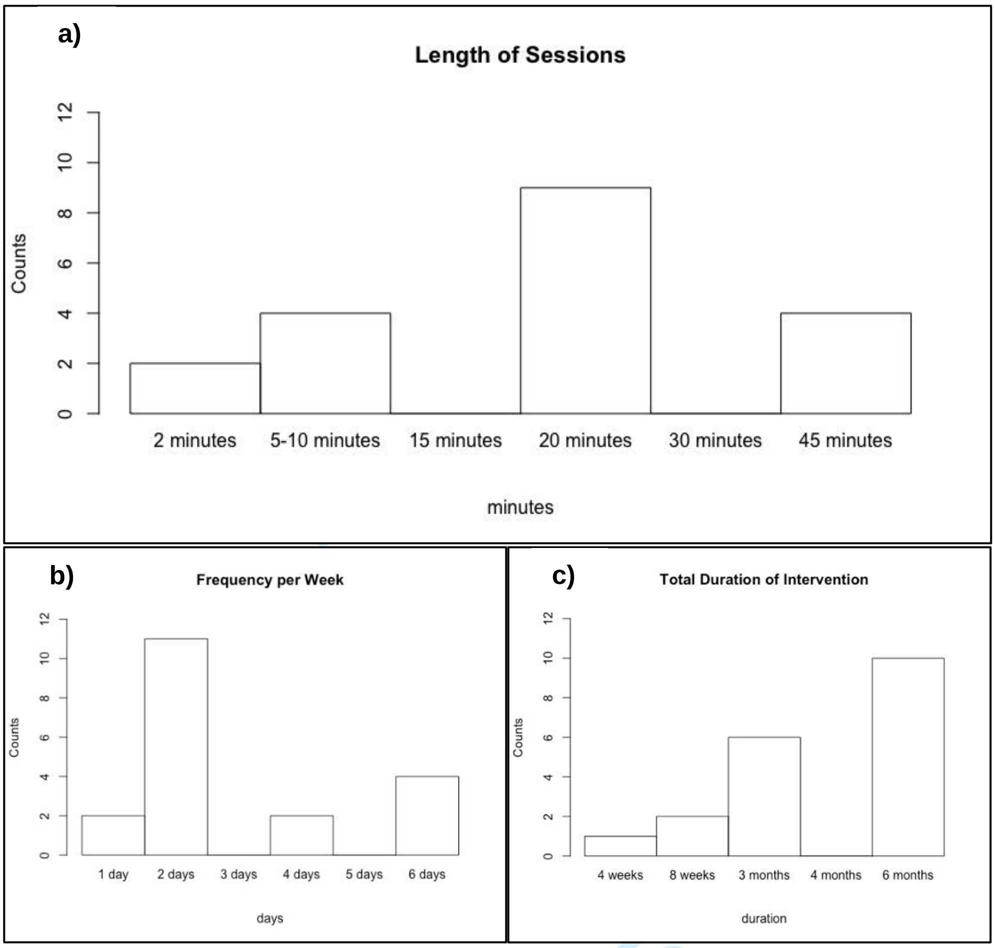

Figure S2. Counts of votes for a) length of sessions, b) frequency per week, and c) duration of the intervention.

1 Calderon J, Bellinger DC. Executive function deficits in congenital heart disease: why is intervention important? *Cardiology in the Young* 2015;**25**:1238–46. doi:10.1017/S1047951115001134

2 Teffer K, Semendeferi K. Chapter 9 - Human prefrontal cortex: Evolution, development, and pathology. In: Hofman MA, Falk D, eds. *Progress in Brain Research*. Elsevier 2012. 191–218. doi:10.1016/B978-0-444-53860-4.00009-X

3 Drzewiecki CM, Juraska JM. The structural reorganization of the prefrontal cortex during adolescence as a framework for vulnerability to the environment. *Pharmacology Biochemistry and Behavior* 2020;**199**:173044. doi:10.1016/j.pbb.2020.173044

4 Long SH, Eldridge BJ, Harris SR, *et al.* Challenges in Trying to Implement an Early Intervention Program for Infants with Congenital Heart Disease. *Pediatric Physical Therapy* 2015;**27**:38–43. doi:10.1097/PEP.0000000000000101

5 Young S, Myanthi Amarasinghe J. Practitioner Review: Non-pharmacological treatments for ADHD: A lifespan approach. *Journal of Child Psychology and Psychiatry* 2010;**51**:116–33. doi:10.1111/j.1469-7610.2009.02191.x

6 Diamond A. Executive Functions: Insights Into Ways to Help More Children Thrive. 2014.

7 Diamond A, Lee K. Interventions Shown to Aid Executive Function Development in Children 4 to 12 Years Old. *Science* Published Online First: 19 August 2011. doi:10.1126/science.1204529

8 Mohammed S, Flores L, Deveau J, *et al.* The Benefits and Challenges of Implementing Motivational Features to Boost Cognitive Training Outcome. *J Cogn Enhanc* 2017;**1**:491–507. doi:10.1007/s41465-017-0047-y

9 Johann VE, Karbach J. Effects of game-based and standard executive control training on cognitive and academic abilities in elementary school children. *Developmental Science* 2020;**23**:e12866. doi:10.1111/desc.12866

10 Cao Y, Huang T, Huang J, *et al.* Effects and Moderators of Computer-Based Training on Children's Executive Functions: A Systematic Review and Meta-Analysis. *Front Psychol* 2020;**11**:580329. doi:10.3389/fpsyg.2020.580329

11 Lumos Labs, Inc. Lumosity ®. 2022.

12 Cognifit. Cognifit Inc © 2022. 2022.

13 Estrada-Plana V, Esquerda M, Mangues R, *et al.* A Pilot Study of the Efficacy of a Cognitive Training Based on Board Games in Children with Attention-Deficit/Hyperactivity Disorder: A Randomized Controlled Trial. *Games for Health Journal* 2019;**8**:265–74. doi:10.1089/g4h.2018.0051

14 Gonçalves PD, Ometto M, Sendoya G, *et al.* Neuropsychological Rehabilitation of Executive Functions: Challenges and Perspectives. *Journal of Behavioral and Brain Science* 2014;**2014**. doi:10.4236/jbbs.2014.41004

15 Thorell LB, Lindqvist S, Bergman Nutley S, *et al.* Training and transfer effects of executive functions in preschool children. *Developmental Science* 2009;**12**:106–13. doi:10.1111/j.1467-7687.2008.00745.x

16 Diamond A, Ling DS. Conclusions about interventions, programs, and approaches for improving executive functions that appear justified and those that, despite much hype, do not. *Developmental Cognitive Neuroscience* 2016;**18**:34–48. doi:10.1016/j.dcn.2015.11.005

For peer review only

17 Gunzenhauser C, Nückles M. Training Executive Functions to Improve Academic Achievement: Tackling Avenues to Far Transfer. *Frontiers in Psychology* 2021;**12**.https://www.frontiersin.org/article/10.3389/fpsyg.2021.624008 (accessed 11 Mar 2022).

18 Barnett SM, Ceci SJ. When and where do we apply what we learn?: A taxonomy for far transfer. *Psychological Bulletin* 2002;**128**:612–37. doi:10.1037/0033-2909.128.4.612

19 Greco G. MULTILATERAL TRAINING USING PHYSICAL ACTIVITY AND SOCIAL GAMES IMPROVES MOTOR SKILLS AND EXECUTIVE FUNCTION IN CHILDREN WITH AUTISM SPECTRUM DISORDER. *European Journal of Special Education Research* Published Online First: 16 March 2020. doi:10.46827/ejse.v0i0.2928

20 Ramezan Saatchi L, Khodabakhsh Pirkalani R, Moradisabzevar M, *et al.* The effect of computerized Cognitive Training package on executive functions of people with major depressive disorder. *Iranian Journal of Psychiatric Nursing* 2022;**10**:39–52. doi:10.22034/IJPN.10.3.39

For peer review only

Einwilligungserklärung

Schriftliche Einwilligungserklärung zur Teilnahme an einer klinischen Studie
Bitte lesen Sie dieses Formular sorgfältig durch. Bitte fragen Sie, wenn Sie etwas nicht verstehen oder wissen möchten. Für die Teilnahme ist Ihre schriftliche Einwilligung notwendig.

**BASEC-Nummer (nach Einreichung):**	
**Titel der Studie (wissenschaftlich und Laiensprache):**	Executive functions intervention in children and adolescents with severe congenital heart disease: a feasibility study (E-Fit)<br><br>Intervention bei Kindern und Jugendlichen mit schweren angeborenen Herzfehlern: eine Machbarkeitsstudie (E-Fit)
**Verantwortliche Institution (Sponsor mit Adresse):**	Prof. Dr. med. Bea Latal<br>Abteilung für Entwicklungspädiatrie<br>Universitäts-Kinderspital Zürich<br>Steinwiesstrasse 75, 8032 Zürich
**Ort der Durchführung:**	Universitäts-Kinderspital Zürich
**Prüfärztin/Prüfarzt am Studienort:**<br>Name und Vorname in Druckbuchstaben:	Prof. Dr. med. Bea Latal
**Teilnehmerin/Teilnehmer:**<br>Name und Vorname in Druckbuchstaben:<br>Geburtsdatum:	____________<br>____________<br>☐ weiblich ☐ männlich

- Ich und mein Kind wurden von der unterzeichnenden Studienkoordination mündlich und schriftlich über den Zweck, den Ablauf der Studie mit E-Fit über mögliche Vor- und Nachteile sowie über eventuelle Risiken informiert.
- Ich und mein Kind nehmen an dieser Studie freiwillig teil und akzeptiere den Inhalt der mir ausgehändigten schriftlichen Information. Ich und mein Kind hatten genügend Zeit, unsere Entscheidung zu treffen.
- Meine Fragen und die meines Kindes im Zusammenhang mit der Teilnahme an dieser Studie sind uns beantwortet worden. Ich und mein Kind behalten die schriftliche Information und erhalten eine Kopie unserer schriftlichen Einwilligungserklärung.
- Ich und mein Kind sind einverstanden, dass die Kinderärztin/der Kinderarzt meines Kindes über die Teilnahme an der Studie informiert wird.
- Ich und mein Kind sind einverstanden, dass die zuständigen Fachleute des Sponsors, der zuständigen Ethikkommission zu Prüf- und Kontrollzwecken in unsere unverschlüsselten Daten Einsicht nehmen dürfen, jedoch unter strikter Einhaltung der Vertraulichkeit.
- Bei Ergebnissen (und/oder Zufallsbefunden), die direkt die Gesundheit meines Kindes betreffen, werden ich und mein Kind informiert. Wenn ich und mein Kind das nicht wünschen, informieren wir die Studienkoordination.
- Ich und mein Kind wissen, dass die gesundheitsbezogenen und persönlichen Daten meines Kindes nur in verschlüsselter Form zu Forschungszwecken für diese Studie (auch im Ausland)

For peer review only - http://bmjopen.bmj.com/site/about/guidelines.xhtml

weitergegeben werden können. Der Sponsor gewährleistet, dass der Datenschutz nach Schweizer Standard eingehalten wird.

- Ich und mein Kind können jederzeit und ohne Angabe von Gründen von der Studienteilnahme zurücktreten. Die weitere medizinische Behandlung meines Kindes ist unabhängig von der Studienteilnahme gewährleistet. Die bis zum Rücktritt erhobenen Daten werden noch im Rahmen der Studie ausgewertet.
- Die Haftpflichtversicherung Universitäts-Kinderspital Zürich kommt für allfällige Schäden auf.
- Bei Zufallsbefunden möchten wir

  a) ☐ in jedem Fall informiert werden
  b) ☐ nicht informiert werden
  c) ☐ die Entscheidung folgender Person überlassen: ....

- Ich und mein Kind sind damit einverstanden, dass die Studienleitenden den aktuellsten kardiologischen Befund beim/bei der zuständigen Kardiolog:in einholen, um den Gesundheitszustand meines Kindes einzuschätzen.

  Name Kardiologe:____________________________

  Name Klinik: ________________________________

- Ich bin damit einverstanden, dass persönliche Daten von mir im Rahmen dieser Studie ausgewertet und verschlüsselt and Drittpersonen innerhalb des Universitäts-Kinderspitals Zürich weitergegeben werden können.
  ☐ Ja ☐ Nein

- Ich und mein Kind sind damit einverstanden, dass persönliche Daten verschlüsselt an Drittpersonen innerhalb des Universitäts-Kinderspitals Zürich weitergegeben werden können.
  ☐ Ja ☐ Nein

- Ich und mein Kind sind damit einverstanden, dass die Daten aus der neuropsychologischen Abklärung verschlüsselt zu Forschungszwecken an CogniFit© weitergegeben werden können.
  ☐ Ja ☐ Nein

- Ich und mein Kind sind damit einverstanden, dass während der neuropsychologischen Abklärung und der Intervention Videoaufnahmen gemacht und verschlüsselt abgespeichert werden.
  ☐ Ja ☐ Nein

- Ich und mein Kind sind damit einverstanden, dass die Hauptlehrperson über die Studienteilnahme informiert und miteinbezogen wird.
  ☐ Ja ☐ Nein

For peer review only

For peer review only - http://bmjopen.bmj.com/site/about/guidelines.xhtml

TEEN HEART STUDY E-FIT

UNIVERSITÄTS-KINDERSPITAL ZÜRICH
Forschungszentrum für das Kind FZK

Die unterzeichnenden Personen bezeugen, dass das Aufklärungsgespräch stattgefunden hat und dass das urteilsfähige Kind in die Studienteilnahme eingewilligt hat und/oder sich keine Anzeichen von Widerstand gegen die Teilnahme erkennen lassen.

Ort, Datum	Unterschrift gesetzliche Vertretung des Kindes

Bestätigung der Studienkoordination: Hiermit bestätige ich, dass ich dieser Teilnehmerin/diesem Teilnehmer Wesen, Bedeutung und Tragweite der Studie erläutert habe. Ich versichere, alle im Zusammenhang mit dieser Studie stehenden Verpflichtungen gemäss in der Schweiz geltenden Rechts zu erfüllen. Sollte ich im Verlauf der Studie von Aspekten erfahren, welche die Bereitschaft der Teilnehmerin/des Teilnehmers zur Studienteilnahme beeinflussen könnten, werde ich sie/ihn umgehend darüber informieren.

Ort, Datum	Name und Vorname der Studienkoordination in Druckbuchstaben<br><br>Unterschrift der Studienkoordination

For peer review only - http://bmjopen.bmj.com/site/about/guidelines.xhtml

TEEN HEART STUDY E-FIT

Acceptance and Feasibility Scale Parents

	Fully disagree			Fully agree
The program was useful for my child.	☐	☐	☐	☐
My child would do the program again.	☐	☐	☐	☐
I would recommend this program to other children.	☐	☐	☐	☐
My child has applied the skills learned.	☐	☐	☐	☐
My child has a plan for dealing with future problems.	☐	☐	☐	☐
My child has made positive changes.	☐	☐	☐	☐
My child has used the skills to become more organized.	☐	☐	☐	☐
My child has used reminder strategies to remember things more often.	☐	☐	☐	☐
My child uses techniques to plan.	☐	☐	☐	☐
My child has met his or her program goals.	☐	☐	☐	☐
My child has a more positive attitude than before.	☐	☐	☐	☐
My child is better at solving problems.	☐	☐	☐	☐
My child remembers things better.	☐	☐	☐	☐
My child is more organized.	☐	☐	☐	☐
My child is better at paying attention and remembering things.	☐	☐	☐	☐
My child is better at getting things done, such as homework or chores.	☐	☐	☐	☐
My child is better at controlling his or her emotions.	☐	☐	☐	☐
My child is better at controlling his or her actions.	☐	☐	☐	☐
My child is better at stopping and thinking before acting.	☐	☐	☐	☐
My child is better able to handle unforeseen changes in plans.	☐	☐	☐	☐
My child feels less stressed.	☐	☐	☐	☐
My child does better in school.	☐	☐	☐	☐

For peer review only - http://bmjopen.bmj.com/site/about/guidelines.xhtml
adapted after Modi et al., 2019

TEEN HEART STUDY E-FIT

Acceptance and Feasibility Scale Children

	Fully disagree			Fully agree
Everything worked as it should.	☐	☐	☐	☐
It was easy to use the online website for the games.	☐	☐	☐	☐
I liked the online games.	☐	☐	☐	☐
It was easy to get in touch with the coach through Microsoft Teams/Zoom.	☐	☐	☐	☐
Meeting with a coach in person would be better.	☐	☐	☐	☐
I felt connected to the coach.	☐	☐	☐	☐
I felt that the coach contributed to my learning beyond the sessions.	☐	☐	☐	☐
The information from the coaching sessions was helpful to me.	☐	☐	☐	☐
I enjoyed the coaching sessions.	☐	☐	☐	☐
I enjoyed the analogue games.	☐	☐	☐	☐
It was easy to understand the game instructions (videos and or brain cards).	☐	☐	☐	☐
The program (as a whole) was too long.	☐	☐	☐	☐
The program (as a whole) was too short.	☐	☐	☐	☐
The program (as a whole) was as I expected it to be.	☐	☐	☐	☐
I liked the program (as a whole).	☐	☐	☐	☐
The content was not relevant to me.	☐	☐	☐	☐
The program (as a whole) was useful to me.	☐	☐	☐	☐
I would do the program (as a whole) again.	☐	☐	☐	☐
I would recommend the program (as a whole) to other children.	☐	☐	☐	☐
What part of the program did you enjoy the most?	☐	☐	☐	☐
What part of the program helped you the most?	☐	☐	☐	☐

	no	yes		
Could any part be dropped?	☐	☐	If yes which one?	

TEEN HEART STUDY
E-FIT

	Fully disagree			Fully agree
I applied the skills I learned during the program.	☐	☐	☐	☐
I have a plan for dealing with future problems.	☐	☐	☐	☐
I made positive changes.	☐	☐	☐	☐
I have used skills to get more organized.	☐	☐	☐	☐
I have used remembering strategies to remember things better.	☐	☐	☐	☐
I have used techniques to plan.	☐	☐	☐	☐
I have achieved the goals I had at the beginning of the program.	☐	☐	☐	☐
I have a more positive attitude than before.	☐	☐	☐	☐
I am better at solving problems.	☐	☐	☐	☐
I am better at remembering things.	☐	☐	☐	☐
I am more organized.	☐	☐	☐	☐
I know strategies that help me pay attention and remember.	☐	☐	☐	☐
I start things, like homework or chores, better than I used to.	☐	☐	☐	☐
I can control my emotions better than I used to.	☐	☐	☐	☐
I can control my actions better than I used to.	☐	☐	☐	☐
I am better able to stop and think before I act.	☐	☐	☐	☐
I can better cope with unforeseen changes in plans.	☐	☐	☐	☐
I feel less stressed.	☐	☐	☐	☐
I do better in school.	☐	☐	☐	☐

For peer review only - http://bmjopen.bmj.com/site/about/guidelines.xhtml
adapted after Modi et al., 2019

Everyday Activities Diary

List of activities: School *1 box = 1 quarter of an hour*

What did your child do today?

Please indicate your child's activities today (to the nearest quarter of an hour)

		06:00	07:00	08:00	09:00	10:00	11:00	12:00
**General**								
Sleeping								
Eating								
**School**								
Lessons								
Physical education								
Recess								
**Leisure time**								
Homework								
Reading or browsing through books on his/her own								
Playing a musical instrument or singing								
Watching TV, videos or DVDs								
Computer or video games, Playstation, Nintendo or the Internet								
Other quite activities or games (listening to music or stories, drawing, painting, handiwork or board games)								
Relatively vigorous games with friends or siblings (playground, role-playing, hide-and-seek, etc.)	Indoor							
	Outdoor							
Vigorous games, running around or ball games (not in a sports club)	Indoor							
	Outdoor							
Training, riding or ballet (in a club)	Indoor							
	Outdoor							
**Travel**								
By foot								
By bicycle, scooter, inline skates or skateboard								
By train, tram or bus								
By car								
**Special**								
Activity difficult to classify								

only

		12:00	13:00	14:00	15:00	16:00	17:00	18:00	19:00	20:00	21:00	22:00	23:00	24:00	01:00	02:00	03:00	04:00	05:00	06:00
**General**																				
Sleeping																				
Eating																				
**School**																				
Lessons																				
Physical education																				
Recess																				
**Leisure time**																				
Homework																				
Reading or browsing through books on his/her own																				
Playing a musical instrument or singing																				
Watching TV, videos or DVDs																				
Computer or video games, Playstation, Nintendo or the Internet																				
Other quite activities or games (listening to music or stories, drawing, painting, handiwork or board games)																				
Relatively vigorous games with friends or siblings (playground, role-playing, hide-and-seek, etc.)	Indoor																			
	Outdoor																			
Vigorous games, running around or ball games (not in a sports club)	Indoor																			
	Outdoor																			
Training, riding or ballet (in a club)	Indoor																			
	Outdoor																			
**Travel**																				
By foot																				
By bicycle, scooter, inline skates or skateboard																				
By train, tram or bus																				
By car																				
**Special**																				
Activity difficult to classify																				

UNIVERSITÄTS-
KINDERSPITAL
ZÜRICH
Forschungszentrum
für das Kind FZK

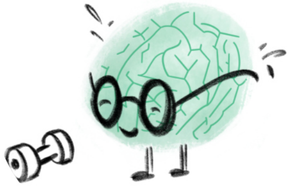

Coaching Content Preparatory Session

Timepoint	Content		Item number
	Explained why we did the assessment	☐ yes ☐ no	1
	Neuroinfo for Kids (NIK) page 3 read aloud	☐ yes ☐ no	2
	Strengths and weaknesses sheet filled in	☐ yes ☐ no	3
	NIK page 5 read aloud	☐ yes ☐ no	4
	NIK page 6 read aloud	☐ yes ☐ no	5
	Results discussed on normal distribution	☐ yes ☐ no	6
	NIK results (2) page read aloud	☐ yes ☐ no	7
	NIK page 10 read aloud	☐ yes ☐ no	8
	Letter written	☐ yes ☐ no	9
	Duration of session 60min	☐ yes ☐ no<br>If no, duration in min: ____________	10

For peer review only

Coaching Content EF Sessions 1-6

**Timepoint**	**Content**		Item number
	Question asked:<br>Were you able to try out what we talked about last week?	☐ yes ☐ no	10
	Question asked:<br>Did you play the card games? (Which ones?)	☐ yes ☐ no	11
	Question asked:<br>Did everything work out with the online games? I saw you played 3x last week and always started 2x, great! (if necessary point out if something was forgotten)	☐ yes ☐ no	12
	Question asked:<br>Did you add the stickers to your poster?	☐ yes ☐ no	13
	Session topic explained	☐ yes ☐ no	14
	Session goal explained	☐ yes ☐ no	15
	All listed pages of the story read aloud/viewed	☐ yes ☐ no	16
	Assignment completed as described in the session guideline	☐ yes ☐ no	17
	Homework assigned	☐ yes ☐ no	18
	Session duration 60min	☐ yes ☐ no<br>If no, duration in min:<br>___________	19

For peer review only - http://bmjopen.bmj.com/site/about/guidelines.xhtml

Coaching Content Last Session

Timepoint	Content		Item number
	Question asked:<br>Were you able to try out what we talked about last week?	☐ yes ☐ no	10.7
	Question asked:<br>Did you play the card games? (Which ones?)	☐ yes ☐ no	11.7
	Question asked:<br>Did everything work out with the online games? I saw you played 3x last week and always started 2x, great! (if necessary point out if something was forgotten)	☐ yes ☐ no	12.7
	Question asked:<br>Did you add the stickers to your poster?	☐ yes ☐ no	13.7
	Overview of this session provided	☐ yes ☐ no	20
	Summary of the last 8 weeks	☐ yes ☐ no	21
	Evaluation questions asked according to guideline	☐ yes ☐ no	22
	How to continue explained according to guideline	☐ yes ☐ no	23
	Questions and comments of the family clarified	☐ yes ☐ no	24
	Session duration 60min	☐ yes ☐ no<br>If no, duration in min:<br>___________	19.7

For peer review only - http://bmjopen.bmj.com/site/about/guidelines.xhtml

Computerized Games

Date	Content		Item number
	Sessions 1&2 completed	☐ yes ☐ no	25
	Sessions 3&4 completed	☐ yes ☐ no	26
	Sessions 5&6 completed	☐ yes ☐ no	27
	Sessions 7&8 completed	☐ yes ☐ no	28
	Sessions 9&10 completed	☐ yes ☐ no	29
	Sessions 11&12 completed	☐ yes ☐ no	30
	Sessions 13&14 completed	☐ yes ☐ no	31
	Sessions 15&16 completed	☐ yes ☐ no	32
	Sessions 17&18 completed	☐ yes ☐ no	33
	Sessions 19&20 completed	☐ yes ☐ no	34
	Sessions 21&22 completed	☐ yes ☐ no	35
	Sessions 23&24 completed	☐ yes ☐ no	36
	Sessions 25&26 completed	☐ yes ☐ no	37

For peer review only

For peer review only - http://bmjopen.bmj.com/site/about/guidelines.xhtml

Analogue Games

Duration	Content		Item number
	Gold	☐ yes ☐ no	38
	Dodelido	☐ yes ☐ no	39
	Särge schubsen	☐ yes ☐ no	40
	Geistesblitz 2.0	☐ yes ☐ no	41
	Beaver Gang	☐ yes ☐ no	42
	Beaver Clan	☐ yes ☐ no	43
	Memo Dice	☐ yes ☐ no	44
	5-er finden	☐ yes ☐ no	45
	The nasty 7	☐ yes ☐ no	46
	Yokai	☐ yes ☐ no	47
	Okiya	☐ yes ☐ no	48
	Perplexus Crazy run 2 PE	☐ yes ☐ no	49
	Perplexus Crazy run 5 FT 208	☐ yes ☐ no	50
	Perplexus Crazy run 6 PE Epic	☐ yes ☐ no	51
	Thunder Pops	☐ yes ☐ no	52

For peer review only - http://bmjopen.bmj.com/site/about/guidelines.xhtml

Supplementary information for the SPIRIT Guidelines

A) Table S3 Dataset information

Data category	Information
Primary registry and trial identifying number	ClinicalTrials.gov<br>NCT05198583
Date of registration in primary registry	19 January, 2022
Source(s) of monetary or material support	Swiss National Science Foundation (SNF 32003B_172914)
Contact for public & scientific queries	Prof. Dr. Beatrice Latal; bea.latal@kispi.uzh.ch
Scientific title	Multimodal personalized executive function intervention (E-Fit) for school-age children with complex congenital heart disease: protocol for a randomized-controlled feasibility study.
Country of recruitment	Switzerland
Health condition(s) or problem(s) studied	Executive dysfunction, severe congenital heart disease
Intervention(s)	Intervention group: executive function intervention (E-Fit)<br>Control group: electronic diary
Key inclusion and exclusion criteria	Ages eligible for study: 10-12 years<br>Sexes eligible for study: both<br>Accepts healthy volunteers: no<br>Inclusion criteria: child born with congenital heart disease (10-12 years), cardiopulmonary bypass surgery prior to one year of age, executive function (EF) deficits<br>Exclusion criteria: heart surgery not performed in Switzerland, known genetic or dysmorphic syndrome
Study type	Interventional allocation: randomized<br>Intervention model: parallel assignment<br>Masking: single blind, outcome assessors<br>Primary purpose: feasibility<br>Phase I
Date of first enrolment	April 2022
Target sample size	40
Recruitment status	Recruiting
Primary Outcome(s)	Feasibility
Key secondary outcomes	Executive function performance

For peer review only

B) Organizational structure and responsibilities

Principal Investigator: Beatrice Latal, Child Development Center, University Children's Hospital Zurich Switzerland

Preparation of protocol and revisions

Publication of study reports

Lead Investigator: Alenka Schmid, Child Development Center, University Children's Hospital Zurich Switzerland

Responsible for identification, recruitment, data collection, and completion of case report forms (CRFs), along with patients adherence to study protocol. Responsible for maintenance of trial IT system and data entry, data verification, and randomization

Responsible for trial master file

Recruitment of patients and liaising with principal investigator

Preparation of CRFs

Design and conduct of E-Fit Feasibility Study

Authorship eligibility guideline

Authors on this paper made a significant contribution to the conception or design of the project or the acquisition, analysis, or interpretation of data for the work, and/or drafted the work or reviewed and revised it critically for important intellectual content.

C) Data management and safety monitoring

Study and questionnaire data are collected by physicians, psychologists, or psychologists in training and recorded in Redcap® [1]. Data from routine visits are derived by a physician from the electronic medical charts of the hospital's data management system, Phoenix, with access limited to the staff of the Children`s Hospital and with strict regulation of data protection. Any prints are locked in a drawer, with access only by the investigator.

Members of the Children's Research Centre of the University Children's Hospital Zurich fulfil the monitoring duties. This includes double checking the data, prestudy monitoring, after enrolment of the 2nd patient, after the 20th patient and a close-out visit. Monitoring covers all study documents, source data, and the trial master file. No interim analyses are planned. This trial is considered as a nonsignificant risk device study and was reviewed accordingly by the Cantonal Ethics Committee of Zurich.

Parents and participants may withdraw from participation at any time. Families are contacted by phone to ascertain the reason for discontinuation as this is important information for the primary outcome of the present study, feasibility. All data collected until drop out will be used for analysis. Serious events and serious adverse events are reported in accordance with the guidelines of the Zurich Cantonal

Ethics Committee, based on the Swiss Federal Act on Research Involving Human Beings. Protocol modifications and amendments will be submitted to the ethical committees for approval. Amendments to the study protocol will be added to publications reporting the study outcomes.

1 Wright A. REDCap: A Tool for the Electronic Capture of Research Data. *J Electron Resour Med Libr* 2016;**13**:197–201. doi:10.1080/15424065.2016.1259026

For peer review only

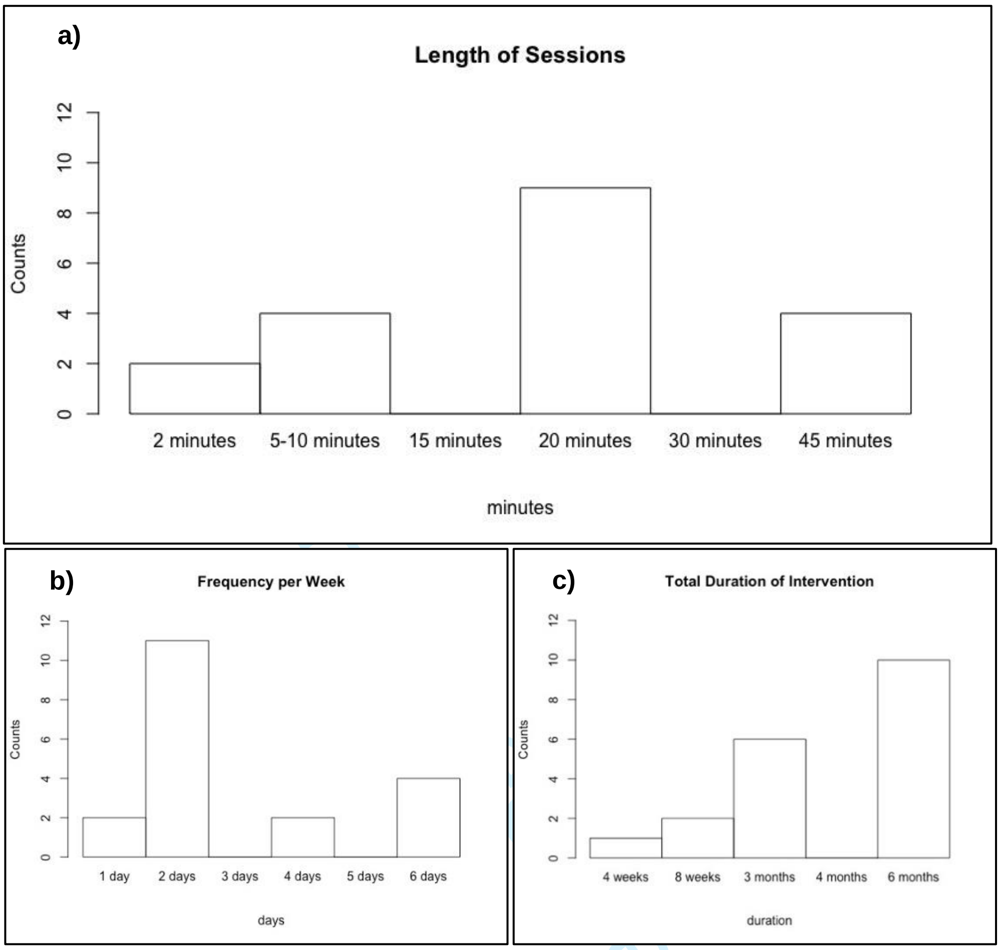

Figure S1. Counts of votes for a) length of sessions, b) frequency per week, and c) duration of the intervention.

For peer review only - http://bmjopen.bmj.com/site/about/guidelines.xhtml

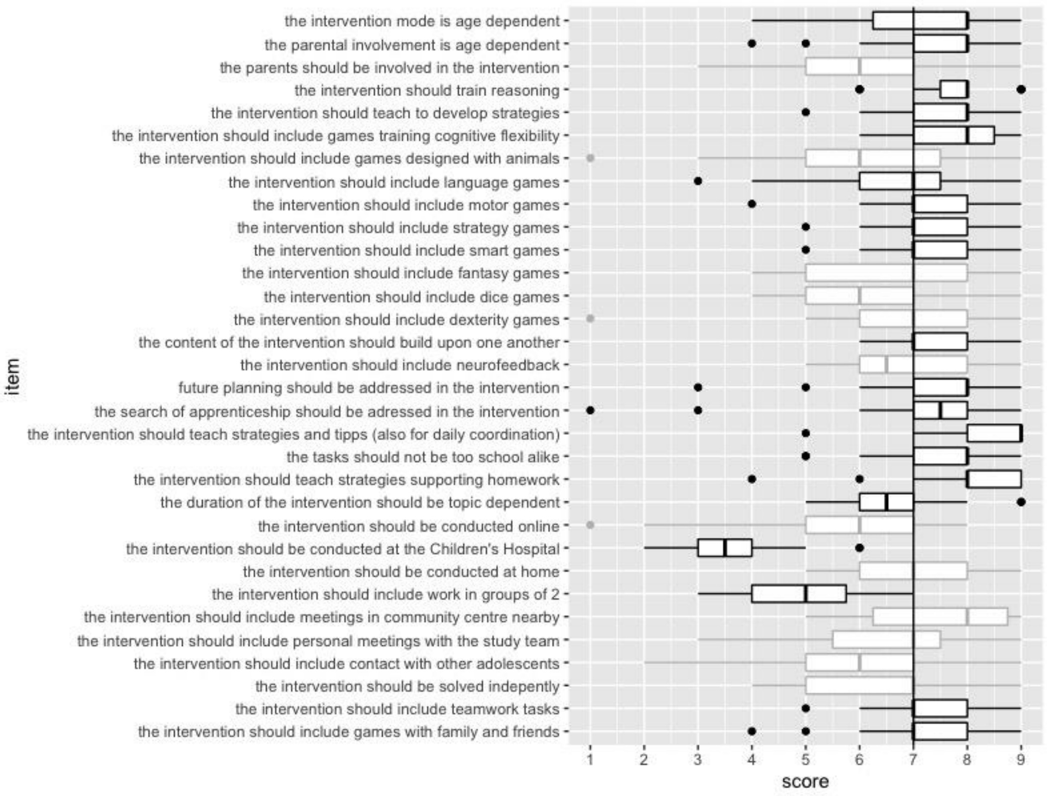

Figure S2. Ratings on Delphi items. 1 = fully disagree, 9 = fully agree. With a median of 7 and an IQR < 2, the item was considered accepted by the participants. Items in gray are the items for which no consensus was found.

For peer review only - http://bmjopen.bmj.com/site/about/guidelines.xhtml

Table S1 Timeline of the study protocol

	Study timeline				
	**Enrolment**	**Allocation**	**Post-Allocation**		
**TIMEPOINT**			***Baseline***	***+ 2 months***	***+ 6 months***
**ENROLMENT:**					
**Informed consent**	x				
**Eligibility screen**	x				
**Allocation**		x			
**INTERVENTIONS:**					
E-Fit Intervention			↔	↔	
Daily Diary			↔	↔	
**ASSESSMENTS:**					
IQ			x		
EF assessment			x	x	x
Questionnaires			x	x	x
Feasibility	x	x	↔	↔	
SES			x		
Medical information			x		

Note : EF = Executive function; SES = socioeconomic status [1]

For peer review only

Table S2 Example week for a participating family

	Exemplar week					
	MO	***TUE***	***WED***	***THU***	***FRI***	***WEEKEND***
**E-FIT INTERVENTION**						
Parents	30min Call					
Child	60min Coaching Session[a]	20min CogniFit[b]		20min CogniFit[b]	20min CogniFit[b]	Card games
**CONTROL GROUP**						
Parents	10min Diary		10min Diary		10min Diary	10min Diary
Child	10min Diary		10min Diary		10min Diary	10min Diary

Note: a = Filling in Session Rating Scale; b = Filling in Children's Feeling scale and Fun and Demand

For peer review only

Table S3. Computerized training (CogniFit Inc © 2022)

Game	Trained skills			
	flexibility	working memory	planning	inhibition
Traffic Manager			x	
Water Lilies		x	x	
Reaction Field	x			x
Happy Hopper				x
Neuron Madness				x
Minus Malus	x	x		
Tennis Bowling	x			
Candy Line Up		x	x	
Bee Balloon	x			
Slice and Drop			x	
Cube Foundry			x	

For peer review only

Table S4. Analog training

Game	Trained skills			
	flexibility	working memory	planning	inhibition
Gold (Game Factory ®)	x	x	x	
Dodelido (“Drei Magier ®”)	x	x		x
Särge schubsen (“Drei Magier ®”)	x	x	x	x
Geistesblitz 2.0 (“Zoch”)	x	x		x
Beaver Gang (“Amigo Spiele”)	x	x	x	
Beaver Clan (“Amigo Spiele”)	x	x	x	
Memo Dice (“Amigo Spiele”)	x	x	x	
5er finden (HABA ®)	x		x	
The nasty 7 (“Drei Hasen in der Abendsonne”)	x	x		x
Yokai (Game Factory ®)	x	x	x	
Okiya (“Pegasus Spiele”)	x		x	
Perplexus Crazy run (2 PE Original, 5 FT 208, 6 PE Epic)				x
Thunder Pops				x

Table S5 Examples of item construction

Categories	Examples	Summary	Item
1 Identity/EF	*"But what I would certainly like for [my son] is … that he is trained a bit. … What should [he] do first, or how can [he] plan the day."*	Daily planning as an everyday problem.	Children with congenital heart defects structure their everyday life
2 Identity/Relation to peers	*"he needs more time and he's a bit slower and then he sees [his] siblings [and] that they're always faster, and then he always makes himself small and that's a shame."*	Is slower than Peers which affects him negatively.	Children with congenital heart defects feel equal compared to their siblings/peers
3 Scope of intervention/game suggestions	*"analog games would also be OK, [I]can imagine that."*	Analog games.	Dice games should be part of such an intervention.
4 Scope of intervention/structure	*"we would like it if it were possible online, because travelling … is always- … a bit far, yes. Especially during school time"*	Online is better for families who live further away from the hospital.	The intervention should be conducted online
5 Scope of intervention/possible distracting factors	*"they just have … a lot of homework …, then he plays the cello, then he goes to the youth club, everything interests him."*	Other hobbies also need a lot of time and should still have room.	Hobbies could get in the way of the intervention
6 Scope of intervention/possible supporting factors	*"That it [should not be like] 'oh that in addition,' but 'great, … that would be great right now and is manageable in terms of time'."*	Time-manageable and fun.	This is the maximum amount of time per day the participants should work on the intervention (in free time):
7 Dealing with the disease/challenges	*"[that] is … something that … she finds quite difficult and where she probably swallows a lot."*	She finds it difficult to talk about her illness and swallows a lot.	Children with congenital heart defects talk about their heart defect
8 Dealing with the disease/supporting factors	*"contact with other heart children, is certainly motivating."*	Contact with other heart children can be motivating.	During the intervention, the children and adolescents should have contact with other children and adolescents
9 Further ideas	*"I think [contact with other children] will motivate them more than if they do their exercise in their corner [or] maybe talk to someone from your team from time to time"*	Contact with other children with CHD or with the study team	In such an intervention, there should be face-to-face meetings with the study team

For peer review only - http://bmjopen.bmj.com/site/about/guidelines.xhtml

1 SPIRIT 2013 Guideline. Schedule of enrolment, interventions, and assessments – GUIDANCE FOR CLINICAL TRIAL PROTOCOLS. https://www.spirit-statement.org/schedule-of-enrolment-interventions-and-assessments/ (accessed 8 Mar 2023).

For peer review only

SPIRIT

STANDARD PROTOCOL ITEMS: RECOMMENDATIONS FOR INTERVENTIONAL TRIALS

SPIRIT 2013 Checklist: Recommended items to address in a clinical trial protocol and related documents*

Section/item	Item No	Description	Page No
**Administrative information**			
Title	1	Descriptive title identifying the study design, population, interventions, and, if applicable, trial acronym	1
Trial registration	2a	Trial identifier and registry name. If not yet registered, name of intended registry	2
	2b	All items from the World Health Organization Trial Registration Data Set	See https://register.clinicaltrials.gov/teenheart
Protocol version	3	Date and version identifier	1
Funding	4	Sources and types of financial, material, and other support	15
Roles and responsibilities	5a	Names, affiliations, and roles of protocol contributors	1, 2_Appendix p.2
	5b	Name and contact information for the trial sponsor	1
	5c	Role of study sponsor and funders, if any, in study design; collection, management, analysis, and interpretation of data; writing of the report; and the decision to submit the report for publication, including whether they will have ultimate authority over any of these activities	15
	5d	Composition, roles, and responsibilities of the coordinating centre, steering committee, endpoint adjudication committee, data management team, and other individuals or groups overseeing the trial, if applicable (see Item 21a for data monitoring committee)	2_Appendix p.2
**Introduction**			
Background and rationale	6a	Description of research question and justification for undertaking the trial, including summary of relevant studies (published and unpublished) examining benefits and harms for each intervention	p. 4-5
	6b	Explanation for choice of comparators	n.a.
Objectives	7	Specific objectives or hypotheses	p. 5

For peer review only

Trial design	8	Description of trial design including type of trial (eg, parallel group, crossover, factorial, single group), allocation ratio, and framework (eg, superiority, equivalence, noninferiority, exploratory)	p. 8
**Methods: Participants, interventions, and outcomes**			
Study setting	9	Description of study settings (eg, community clinic, academic hospital) and list of countries where data will be collected. Reference to where list of study sites can be obtained	p. 8
Eligibility criteria	10	Inclusion and exclusion criteria for participants. If applicable, eligibility criteria for study centres and individuals who will perform the interventions (eg, surgeons, psychotherapists)	p. 8
Interventions	11a	Interventions for each group with sufficient detail to allow replication, including how and when they will be administered	p.9-10 3_Appendix
	11b	Criteria for discontinuing or modifying allocated interventions for a given trial participant (eg, drug dose change in response to harms, participant request, or improving/worsening disease)	n.a.
	11c	Strategies to improve adherence to intervention protocols, and any procedures for monitoring adherence (eg, drug tablet return, laboratory tests)	3_Appendix p.1
	11d	Relevant concomitant care and interventions that are permitted or prohibited during the trial	n.a.
Outcomes	12	Primary, secondary, and other outcomes, including the specific measurement variable (eg, systolic blood pressure), analysis metric (eg, change from baseline, final value, time to event), method of aggregation (eg, median, proportion), and time point for each outcome. Explanation of the clinical relevance of chosen efficacy and harm outcomes is strongly recommended	p.11-13
Participant timeline	13	Time schedule of enrolment, interventions (including any run-ins and washouts), assessments, and visits for participants. A schematic diagram is highly recommended (see Figure)	p.19
Sample size	14	Estimated number of participants needed to achieve study objectives and how it was determined, including clinical and statistical assumptions supporting any sample size calculations	p.12

For peer review only - http://bmjopen.bmj.com/site/about/guidelines.xhtml

Recruitment	15	Strategies for achieving adequate participant enrolment to reach target sample size	p. 8
**Methods: Assignment of interventions (for controlled trials)**			
Allocation:			
Sequence generation	16a	Method of generating the allocation sequence (eg, computer-generated random numbers), and list of any factors for stratification. To reduce predictability of a random sequence, details of any planned restriction (eg, blocking) should be provided in a separate document that is unavailable to those who enrol participants or assign interventions	p.9
Allocation concealment mechanism	16b	Mechanism of implementing the allocation sequence (eg, central telephone; sequentially numbered, opaque, sealed envelopes), describing any steps to conceal the sequence until interventions are assigned	p.9
Implementatio n	16c	Who will generate the allocation sequence, who will enrol participants, and who will assign participants to interventions	p.9
Blinding (masking)	17a	Who will be blinded after assignment to interventions (eg, trial participants, care providers, outcome assessors, data analysts), and how	p.8 - 9
	17b	If blinded, circumstances under which unblinding is permissible, and procedure for revealing a participant's allocated intervention during the trial	n.a.
**Methods: Data collection, management, and analysis**			
Data collection methods	18a	Plans for assessment and collection of outcome, baseline, and other trial data, including any related processes to promote data quality (eg, duplicate measurements, training of assessors) and a description of study instruments (eg, questionnaires, laboratory tests) along with their reliability and validity, if known. Reference to where data collection forms can be found, if not in the protocol	2_Appendix p.2
	18b	Plans to promote participant retention and complete follow-up, including list of any outcome data to be collected for participants who discontinue or deviate from intervention protocols	2_Appendix p.2
Data management	19	Plans for data entry, coding, security, and storage, including any related processes to promote data quality (eg, double data entry; range checks for data values). Reference to	2_Appendix p.2

For peer review only

		where details of data management procedures can be found, if not in the protocol	
Statistical methods	20a	Statistical methods for analysing primary and secondary outcomes. Reference to where other details of the statistical analysis plan can be found, if not in the protocol	p.13
	20b	Methods for any additional analyses (eg, subgroup and adjusted analyses)	n.a.
	20c	Definition of analysis population relating to protocol non-adherence (eg, as randomised analysis), and any statistical methods to handle missing data (eg, multiple imputation)	p.13
**Methods: Monitoring**			
Data monitoring	21a	Composition of data monitoring committee (DMC); summary of its role and reporting structure; statement of whether it is independent from the sponsor and competing interests; and reference to where further details about its charter can be found, if not in the protocol. Alternatively, an explanation of why a DMC is not needed	2_Appendix p.2
	21b	Description of any interim analyses and stopping guidelines, including who will have access to these interim results and make the final decision to terminate the trial	n.a.
Harms	22	Plans for collecting, assessing, reporting, and managing solicited and spontaneously reported adverse events and other unintended effects of trial interventions or trial conduct	2_Appendix p.2
Auditing	23	Frequency and procedures for auditing trial conduct, if any, and whether the process will be independent from investigators and the sponsor	n.a.
**Ethics and dissemination**			
Research ethics approval	24	Plans for seeking research ethics committee/institutional review board (REC/IRB) approval	p.13
Protocol amendments	25	Plans for communicating important protocol modifications (eg, changes to eligibility criteria, outcomes, analyses) to relevant parties (eg, investigators, REC/IRBs, trial participants, trial registries, journals, regulators)	2_Appendix p.3
Consent or assent	26a	Who will obtain informed consent or assent from potential trial participants or authorised surrogates, and how (see Item 32)	p.8
	26b	Additional consent provisions for collection and use of participant data and biological specimens in ancillary studies, if applicable	n.a.

For peer review only

Confidentiality	27	How personal information about potential and enrolled participants will be collected, shared, and maintained in order to protect confidentiality before, during, and after the trial	2_Appendix p.2
Declaration of interests	28	Financial and other competing interests for principal investigators for the overall trial and each study site	p. 1
Access to data	29	Statement of who will have access to the final trial dataset, and disclosure of contractual agreements that limit such access for investigators	2_Appendix p.2
Ancillary and post-trial care	30	Provisions, if any, for ancillary and post-trial care, and for compensation to those who suffer harm from trial participation	n.a.
Dissemination policy	31a	Plans for investigators and sponsor to communicate trial results to participants, healthcare professionals, the public, and other relevant groups (eg, via publication, reporting in results databases, or other data sharing arrangements), including any publication restrictions	p.13
	31b	Authorship eligibility guidelines and any intended use of professional writers	2_Appendix p.2
	31c	Plans, if any, for granting public access to the full protocol, participant-level dataset, and statistical code	n.a.
**Appendices**			
Informed consent materials	32	Model consent form and other related documentation given to participants and authorised surrogates	1_Appendix
Biological specimens	33	Plans for collection, laboratory evaluation, and storage of biological specimens for genetic or molecular analysis in the current trial and for future use in ancillary studies, if applicable	n.a.

*It is strongly recommended that this checklist be read in conjunction with the SPIRIT 2013 Explanation & Elaboration for important clarification on the items. Amendments to the protocol should be tracked and dated. The SPIRIT checklist is copyrighted by the SPIRIT Group under the Creative Commons "Attribution-NonCommercial-NoDerivs 3.0 Unported" license.

For peer review only - http://bmjopen.bmj.com/site/about/guidelines.xhtml

BMJ Open

Multimodal personalized executive function intervention (E-Fit) for school-age children with complex congenital heart disease: protocol for a randomized-controlled feasibility study.

Journal:	*BMJ Open*
Manuscript ID	bmjopen-2023-073345.R1
Article Type:	Protocol
Date Submitted by the Author:	23-Aug-2023
Complete List of Authors:	Schmid, Alenka; Universitäts-Kinderspital Zürich,<br>Ehrler, Melanie; University Children Hospital Zurich, Child Development Center; University Children's Hospital Zurich, Children's Research Center<br>Wehrle, Flavia; University Children’s Hospital Zurich, Child Development Center; University Children’s Hospital Zurich, Children’s Research Center<br>Tuura, Ruth; University Children's Hospital of Zurich, Child Development Center; University Children's Hospital Zurich, Children's Research Center<br>Kretschmar, Oliver; Universitäts-Kinderspital Zürich, Department of Pediatric Cardiology; University of Zurich Faculty of Medicine,<br>Landolt, Markus; University Children's Hospital Zurich, Department of Psychosomatics and Psychiatry; University of Zurich, Division of Child and Adolescent Health Psychology<br>Latal, Beatrice; University Children’s Hospital Zurich, Child Development Center; University of Zurich
**Primary Subject Heading**:	Paediatrics
Secondary Subject Heading:	Patient-centred medicine, Qualitative research
Keywords:	Developmental neurology & neurodisability < PAEDIATRICS, Congenital heart disease < CARDIOLOGY, Adolescent, Feasibility Studies

SCHOLARONE™
Manuscripts

Multimodal personalized executive function intervention (E-Fit) for school-age children with complex congenital heart disease: protocol for a randomized-controlled feasibility study.

Alenka Schmid[1], Melanie Ehrler[1,2,3], Flavia M. Wehrle[1,2,5], Ruth O'Gorman Tuura[1,2,4,6], Oliver Kretschmar[2,4,7], Markus A. Landolt[2,8,9], Beatrice Latal[1,2,3,4]

[1]Child Development Center, University Children's Hospital Zurich, Zurich, Switzerland

[2]Children's Research Center, University Children's Hospital Zurich, Zurich, Switzerland

[3]URPP Adaptive Brain Circuits in Development and Learning, University of Zurich, Zurich, Switzerland

[4]University of Zurich, Zurich, Switzerland

[5]Department of Neonatology and Intensive Care, University Children's Hospital Zurich, Zurich, Switzerland

[6]MR Research Centre, University Children's Hospital Zurich, Zurich, Switzerland

[7]Department of Cardiology, University Children's Hospital Zurich, Zurich, Switzerland

[8]Department of Psychosomatics and Psychiatry, University Children's Hospital Zurich, Zurich, Switzerland

[9]Division of Child and Adolescent Health Psychology, Department of Psychology, University of Zurich, Zurich, Switzerland

ORCID iDs

Alenka Schmid https://orcid.org/0000-0001-5013-0530

Melanie Ehrler https://orcid.org/0000-0003-4253-2016

Flavia M. Wehrle https://orcid.org/0000-0001-5992-0424

Markus A. Landolt https://orcid.org/0000-0003-0760-5558

Correspondence to

Prof. Dr. Beatrice Latal; bea.latal@kispi.uzh.ch

Child Development Center

University Children's Hospital Zurich

Steinwiesstrasse 75, 8032 Zurich, Switzerland

Protocol version

17.08.23, Version 2.0

Keywords: Feasibility Studies; Developmental neurology & neurodisability; Congenital heart disease; Adolescent

For peer review only

Abstract

Introduction

Children with congenital heart disease (CHD) are at risk for executive functions (EF) impairments. To date, interventions have limited effects on EF in children and adolescents with complex CHD. Therefore, we developed a new multimodal and personalized EF intervention. This study aims to test the feasibility of this intervention called "E-Fit" for children with complex CHD and EF impairments.

Methods and analysis

This is a single-center, single-blinded randomized-controlled feasibility study, exploring the E-Fit intervention. We aim to enroll 40 children with CHD aged 10 to 12 years, who underwent infant cardiopulmonary bypass surgery, and show clinically relevant EF impairments (T-score ≥ 60 on any Behavior Rating Inventory for Executive Function questionnaire summary scale). The multimodal intervention was developed with focus groups and the Delphi method involving children and adolescents with CHD, their parents and teachers, and health professionals. The intervention is composed of three elements: computer-based EF training using CogniFit Inc © 2022, performed three times a week at home; weekly EF remote strategy coaching; and analog games. The content of the computer and strategy training is personalized to the child's EF difficulties. The control group follows their daily routines as before and completes a diary about their everyday activities four times a week. 1:1 randomization allocates participants. Feasibility is measured by the participants' and providers' ratings of the participants' adherence and exposure to the intervention, recruitment rates, and the evaluation of the intended effects of the program.

Ethics and Dissemination

The local ethics committee approval was obtained (BASEC-Nr: 2021-02413). Parents provide written informed consent. Key outputs from the trial will be disseminated through presentations at conferences, peer-reviewed publications, and directly to participating families. Further these results will inform the decision whether to proceed to a randomized controlled trial to investigate effectiveness.

Registration details

ClinicalTrials.gov: NCT05198583

Article Summary

Strengths and Limitations

For peer review only

- The intervention was designed based on extensive input from patients with CHD, families, teachers, and health professionals provided through focus group interviews and the Delphi method.
- Using a multiple-domain approach, the intervention will be personalized towards individual patients' and families' needs.
- Assessors are blinded to the group allocation of participants.
- Families of the control group may be aware of the content of the intervention and engage in its activities which might reduce the effect sizes of the intervention.
- Considering the limited sample size of this feasibility study, the effectiveness of the training will only be estimated exploratorily.

For peer review only

1. Introduction

Eight out of 1000 children are born with congenital heart disease (CHD; [1]); of these, one third require open heart surgery during infancy [2]. In these children, neurodevelopmental problems are known to be a main comorbidity [3]. Studies have shown that the mean IQ of children with CHD is within the normal range but lower than that of their healthy peers [4]. Despite this normal intellectual functioning, children with complex CHD, particularly, are at risk for higher-order cognitive deficits: executive function (EF) impairments [4,5]. The main EF are inhibition; cognitive flexibility, including fluency; planning; and working memory [4–10]. Children with CHD show mild to moderate impairments in all these EF domains [4,5] without a clear predominance of any specific EF domain. In typically developing children, EF are more closely related to school readiness even than IQ, and EF predict academic achievement [11]. Accordingly, EF difficulties in childhood have been related to higher unemployment rates in adulthood [12]. Further, EF difficulties in adulthood can increase the risk of addictive behavior, and psychiatric diseases such as anxiety and depression [13,14].

Many sequelae of EF difficulties have also been demonstrated in children with CHD. They face lower school achievement and lower psychosocial quality of life (QoL) during school age [15,16]. Further, lower EF have been associated with a higher rate of internalizing symptoms in adolescents and adults with CHD [17]. Given the lifelong academic, psychological, and social burden associated with EF difficulties, evidence-based interventions improving EF functions are urgently needed, particularly for children and adolescents with CHD [18,19].

1.1 EF Intervention

To date, only two studies have investigated the efficacy of EF interventions in children with CHD. Both studies included school-aged children and adolescents with CHD and tested Cogmed Working Memory Training (Cogmed; [20,21]). Cogmed is a computerized program designed to improve attention and working memory, a core dimension of EF [22]. Participants completed regular Cogmed sessions at home over for at least 5 weeks [21,23]. These studies demonstrated inconsistent findings: Calderon et al. found no improvement of working memory either at postintervention or at 3-month follow up, but participants who did the Cogmed sessions showed significant improvements in inhibitory control and self-regulation at the 3-month follow up. Parents reported improved cognitive regulatory skills at postintervention and at 3-month follow up [20].

In contrast, Jordan et al. found improved working memory postintervention in participants who were assigned to Cogmed compared to the control group. However, this improvement did not persist longer than 6-months follow up [21]. These findings are in line with the inconsistent results of a recent meta-analysis of EF

For peer review only

interventions in other children at risk for EF impairments [24]. This meta-analysis reported only small to moderate effects of computerized interventions of single functions and no evidence for long-term effects. These inconsistencies between EF interventions have led to investigations about factors impacting the effectiveness of an EF intervention [25]. One of those factors is motivation, which influences the effort a participant is willing to make, which further impacts the intervention's success [26]. Game elements are able to increase motivation and therefore, utilizing computerized interventions with game elements are a favorable option [27]. Also, analog games, such as board and card games have been tested as EF interventions and have shown effectiveness in enhancing the functions trained [28,29]. Moreover, by requiring children to play with others, such games may enhance self-monitoring [28]. Studies combining computer and analog games demonstrated improvements in trained reasoning and speed tasks in healthy children with low socioeconomic status aged 7 to 10 years [30]. However, despite the enjoyment factor, a major challenge in interventional studies is the limited transfer of training effects to other cognitive abilities [31–34]. Importantly, a meta-analysis revealed that programs emphasizing teaching social-emotional strategies and self-regulation had the most significant positive impact on EF, particularly for children with neurodevelopmental disorders or behavioral problems. These effects appeared to be stronger than those observed in trainings primarily focusing on tasks specifically training EF and may address the transfer of training to general cognitive abilities [18,24,31]. Therefore, exploring intervention approaches beyond computerized and tasks specific training is crucial [20]. Mindfulness education has improved EF behavior, optimism, and classroom social competence behaviors in healthy children and adolescents [35,36]. Similarly, problem-solving solutions to EF-related challenges in everyday life improved parent- and self-rated global EF behaviors in children with epilepsy [37]. Hence, mindfulness interventions and strategy coaching may address the transfer of training to everyday life by targeting sensory awareness and attention regulation, thus promoting emotional and social development [18,31].

Combining established and engaging computerized and analog training that improves individual EF, alongside strategy coaching aiming at integrating these benefits into daily life, presents a promising approach for addressing the aforementioned limitations of current interventions.

Because children with CHD experience difficulties in various EF domains with significant academic, psychological, and social consequences, an intervention integrating several of these approaches may be particularly suited to improving these children's EF and, eventually, their long-term development and social integration.

For peer review only

1.2 Aims

This study aims to test the feasibility of a multimodal personalized EF intervention called E-Fit for children with complex CHD and EF impairments. To evaluate E-Fit's feasibility, the following hypotheses will be explored:

- Children with CHD and EF impairments adhere to at least 80% of the strategy coaching and computerized training sessions.
- Children with CHD and EF impairments and their parents find all components of the intervention suitable, enjoyable and report a high level of satisfaction, all rated as 3 or higher on a 4-point rating scale.
- Children with CHD and EF impairments can integrate all components of the E-Fit intervention into their lives and environment, as it is designed to be user-friendly and easily accessible, all rated as 3 or higher on a 4-point rating scale.
- Although exploratory due to its small sample size, we anticipate a moderate effect size of the E-Fit intervention for improving EF in children with CHD and EF impairments pre- to post-intervention

The rationale for projection numbers provided in the hypotheses is as follows:

Adherence of 80% is based on previous intervention studies [23].

The decision to use rating scale ratings of 3 or higher is founded on the 4-point rating scale (1 = do not agree at all - 4 fully agree), Therefore, 3 and above are considered affirmative.

Effect sizes are based on results of a meta-analysis. Specifically, a value of .38 pertains to explicit EF practice (including computerized and analog training, across all populations), while .46 corresponds to strategy coaching across all populations [24].

2. Methods and analysis

In this section, we will first describe the development of the intervention, which involved a needs assessment preceding the present investigation. Subsequently, we will outline the methods utilized in the present feasibility study. For an overview, see figure 1.

2.1 Needs assessment

Before developing E-Fit and the present study, we conducted a needs assessment. This assessment aimed to specifically identify the unique EF intervention needs of children with CHD by means of focus group interviews and the Delphi method. Below, you will find comprehensive details about the focus group interviews and Delphi method, including the outcomes that led to the final intervention.

For peer review only

2.1.1 Focus group interviews

We conducted two separate online focus group interviews: one with five adolescents with CHD aged 14 to 16 years and another with seven of their parents (n_{female}= 5). Participants were recruited from the Teen Heart Study, which investigated EF in children and adolescents with CHD at the University Children's Hospital Zurich. The Teen Heart Study included children and adolescents with CHD without genetic or dysmorphic syndromes who were born between 2004 and 2012 and underwent their first cardiopulmonary bypass (CPB) surgery before the age of 6 years [25]. Participants were asked what they consider the ideal content and scope of an intervention supporting EF in children with CHD (e.g.: 1. how much time can or should your child invest per a) week, b) per day, c) how long in total? 2. Which difficulties does your child have in everyday life?, see 1_Appendix). These interviews were recorded with the consent of the participants and then transcribed and analyzed with structured qualitative content analysis [26]. This analysis produced nine categories, and all participants' suggestions and statements were assigned to one of these categories (table S1, supplemental_material).

2.1.2 Delphi method

The results from the focus group interviews informed the initiation of the Delphi method, aiming to achieve consensus on the intervention's content through three sequential questionnaires [27,28]. During this procedure, the items of a survey are rated and evaluated multiple times, each time anonymously. The participants subsequently receive the next survey, along with feedback on the overall answers [29].

The surveys were completed online by children with CHD (*n* = 7) aged 12 to 13 years who had not participated in the preceding focus group interviews, their teachers (*n* = 5), parents (*n* = 7), and health professionals (n = 5). The health professionals were experts in the field from international hospitals and research institutions, who held a comprehensive understanding of the difficulties faced by children with CHD. They had previously engaged with the research group and were contacted by email. The families were again recruited from the Teen Heart Study, but following focus group interview suggestions, only participants between 10 and 13 years were asked to participate. The families provided the email addresses of the teachers to be contacted by the study team. Hence, the surveys were completed by four stakeholder groups: children with CHD, parents of children with CHD, teachers of children with CHD, and health professionals.

For survey one, respondents received a link by email and were invited to rate each item on a Likert scale from 1 for "fully disagree" to 9 for "fully agree". They were encouraged to make further suggestions yet to be represented in the survey. Survey two was sent to all respondents of survey one. All items from survey one were included, and additional items suggested by participants were added following revision and categorization. The results of survey one were included in survey two: A table displayed the median and

For peer review only

interquartile range (IQR) per stakeholder group for each item, and each participant was reminded of the scores they had given in survey one. Participants were asked to consider the input of all stakeholder groups in survey one and then review their answers. Items reaching consensus (IQR <2) were dropped from the subsequent round. Survey three was sent to all respondents of survey two. Items for which no consensus was reached in survey two were included. No more items were added. Again, the results of survey two were included in survey three. Participants again reviewed their answers. At the end of survey three, every participant was asked about their interest in participating in the final consensus meeting. Throughout the process, all stakeholder groups responded to the same items.

Overall, participants completed three rounds with the goal of consensus-seeking across stakeholder groups, defined as IQR<2 for each item [30]. Items for which no consensus was reached after three rounds were discussed in a final consensus meeting with three parents, two health professionals, and the study team. In the consensus meeting it became clear that some aspects of the intervention are child-specific and cannot be answered conclusively. To families it was particularly important to have a diverse intervention that was not too school-like. The researchers commented on the intervention in terms of methodological aspects, which were taken into account in the study design (e.g. additional questionnaires or incentives to be added).

2.1.3 Findings from needs assessment

The results of the Delphi method, including the consensus meeting, showed that the wishes most strongly expressed by parents were that the intervention should include strategies supporting homework and everyday life activities and that the children should learn to develop their own strategies. Families participating in the Delphi method wanted activities that did not resemble schoolwork, improved reasoning skills, and addressed academic future planning (figure S1, supplemental_material). Parents emphasized that the ideal age at which an EF intervention should start depends on the individual child, ranging from 4 to 18. Literature shows that EF development progresses considerably during childhood and adolescence [18]. This opens a window of opportunity for EF training during these periods [31,32]. Also, the families' perceived need for therapy is crucial for adherence [33]. During school age and adolescence, families become more aware of the sequelae of CHD and thus become more willing to participate in an intervention program. Participants also wanted minimal parental participation in the intervention, and the focus group interviews indicated that participants became aware of EF problems shortly before high school when remedial action might still be effective.

Consequently, we decided on an age range of 10 to 12 years instead of younger or older. No consensus was reached on whether the intervention should include personal meetings with the study team, whether the intervention should occur in a nearby community center, or on the design of the games (figure S1,

For peer review only - http://bmjopen.bmj.com/site/about/guidelines.xhtml

supplemental_material). The consensus meeting indicated that responses on these topics differed between children and families, and general agreement was difficult to reach. Hence, literature and practice in previous programs were used to decide on these elements [18,20,21,33–39]. For details, see 2_Appendix and figure S2, supplemental_material.

2.2 Study design to test the feasibility of the intervention

This is a single-center, single-blinded randomized-controlled feasibility study to test the feasibility of the E-Fit intervention.

All participants undergo a baseline neurodevelopmental assessment (table 1) and are randomly then assigned to either the E-Fit intervention or to the control group. All participants undergo a 2-month postintervention and a 6-month follow-up assessment. Investigators administering neuropsychological tests are blinded to the participant's allocation. The families and the psychologist who conducts the intervention are aware of the allocation (table S2, supplemental_material).

2.3 Participants and recruitment

This study includes children with CHD who underwent CPB in Switzerland before one year of age. Diagnoses may include transposition of the great arteries, hypoplastic left heart syndrome, and other complex CHDs requiring CPB. At recruitment, children are 10-12 years old. Children diagnosed with a genetic or dysmorphic syndrome such as trisomy-21 or Ellis-van Creveld syndrome or with known large cerebral lesions or injuries, including stroke and severe hypoxic-ischemic encephalopathy with significant neurological sequelae preventing them from performing the assessments are excluded.

Recruitment for the study commenced in April 2022 and is planned to continue until fall 2023. Children from the Department of Cardiology outpatient clinic at the University Children's Hospital Zurich are being contacted consecutively. Follow-up assessments are scheduled to be completed by the beginning of 2024. Participants are identified through the clinical database comprising all treated children with CHD. All eligible children with CHD and their parents receive a letter with the study information and are asked to contact the study team if they are interested in participating. The study team provides further information about the study and answers questions on the phone. The contact information of the children's teachers are forwarded by the parents and after agreeing to participate and giving written informed consent (3_Appendix) parents and the child's principal teacher fill in the Behavior Rating Inventory of Executive Functions (BRIEF [40]) screening questionnaire. The BRIEF assesses EF behaviors in children and adolescents on three summary scales: a Behavior Regulation

For peer review only

Index, a Metacognition Index, and a Global Executive Composite. Participants are included in the study if a T-score ≥ 60 on any of the three summary scales is reported by either parents or teachers.

2.4 Randomization and stratification

After passing the screening questionnaire, participants will be randomly assigned to either the intervention or control group in a 1:1 design by block randomization (block size = 4, stratification variable: maternal education level on a 6-point Likert scale). This list of random numbers will be generated prior to the start of the study in the statistical software R [41] and implemented in the randomization module on the research electronic database Redcap® [42], which allows allocation to be concealed until the participant is enrolled. At the end of the first assessment, after all tests and questionnaires are completed, the child will open an envelope prepared by a study member otherwise not involved in the study. The note in the envelope will reveal the allocation status to the family and baseline assessor.

2.5 Intervention group: E-Fit

The executive function intervention (E-Fit) targets multiple EF domains, focusing on four that have been identified as being impaired by two meta-analyses on EF in children with CHD: inhibition, planning, flexibility, and working memory [4,5]. See 4_Appendix for details. These are addressed in a multimodal approach consisting of three personalized and adaptive elements: 1) computerized training played alone, 2) remote 1:1 strategy coaching with the child and a psychologist, and 3) analog board and card games fostering EF abilities that can be played alone or with friends and family (table S3, supplemental_material). The intervention lasts for eight weeks.

Computerized training

Participants use the platform CogniFit Inc © 2022 (CogniFit) for the computerized training. CogniFit was selected based on literature [43,44] and its successful use in other similarly aged populations [45] and because it targets several EF domains. This is in contrast to Cogmed, which only targets working memory. From the CogniFit platform, eleven games targeting the aforementioned EF domains were selected considering three games per function (table S4, supplemental_material). These games involve tasks that require information retention and manipulation, strategy development and task switching [34]. These games are adaptive to the child's level of performance. Participants play the assigned games at home on a computer, smartphone, or tablet. A device is provided if needed. The child is asked to play three times a week for 20 min over the course of the eight-weeks intervention period.

Strategy Coaching

For peer review only

The strategy coaching is conducted remotely. A psychologist is assigned to each family as a coach to monitor and support them throughout the intervention. The child meets with the coach once a week for 60 minutes for a total of eight sessions.

The first session discusses baseline assessment results and the child's strengths and weaknesses [46]. Further, the first module of CogniFit is performed to make sure the child understands how to use CogniFit. In this session, a parent participates with the child.

For the six following sessions, parents fill in a short questionnaire [35] to identify EF domains in which their child faces difficulties. Of eleven EF domains rated, the six indicating the most difficulties are selected for the coaching sessions. Each of these six sessions has standardized guidelines and starts with a story about an EF and an explanation of how this function engages our brain [36–39]. Afterwards, the child completes a task teaching a strategy for the EF in question [47] (for more details, see 4_Appendix). During the coaching sessions, solutions to difficulties with adhering to E-Fit are suggested.

The eighth and final coaching session consists of the wrap-up and outlook, where a parent again participates with the child [48].

Additionally, the coach calls the parents in the first and fourth week to discuss the upcoming sessions that will be conducted with the child only. Parents can suggest specific situations to be addressed in the subsequent strategy coaching. In response, the coach provides tips on how parents can support their child with the respective EF [47]. For an overview of the strategy coaching procedure, see table 2.

Following the manual, the strategy coaching teaches strategies to promote EF in everyday life [47]. A clinical neuropsychologist reviewed the strategy coaching, and the manual is available in German language.

Analog training

Thirteen board and card games are provided to the families (table S5, supplemental_material). They are suited for this age group and target EF abilities. A special needs and trained game educator helped select the games targeting the above-mentioned EF domains for children between 10 to 12 years of age. Like the computerized training, the analog training also demands a range of diverse functions, including rule retention, flexible adaptation to other players, impulse control, and strategic thinking. Often, a single game may require multiple functions simultaneously. Families are free to play as much as they want, but are asked to report the date, duration, and name of the analog games played.

For peer review only

2.6 Control group

The control group receives standard of care, including cardiac surveillance, neurodevelopmental counseling, and therapies such as speech and physiotherapy. Because the control group families know the scope of the tested intervention, this may lead to contamination: during the trial, parents of control children may engage their children in activities used in the intervention fostering EF functions. To detect this and where occurring, to measure the time spent on it, parents and children complete an electronic diary four times a week for eight weeks to monitor the child's everyday activities (5_Appendix and table S3, supplemental_material). After the final visit at the end of study participation, the control group families receive the list of EF-enhancing analog games used, EF fact sheets, and a 2-month CogniFit login.

2.7 Primary outcome measures

The feasibility of the E-Fit intervention will be measured in six areas, as described by Bowen et al. [49]. Except for Practicality and Limited efficacy, primary outcome measures will be collected only in the intervention group throughout the intervention period. For a summary of measures and assessment timepoints see table 3. The following areas will be assessed.

Acceptability

Acceptability examines suitability, satisfaction, and attractiveness to program deliverers and recipients. It is measured by questions about satisfaction, intent to continue use, and perceived appropriateness throughout the intervention period.

Demand

Demand is assessed by the extent to which the intervention is likely to be used or how much demand exists. This includes measures for the fit within the family, perceived positive or negative effects on the family, actual use, expressed interest or intention to use, and perceived demand throughout the intervention period.

Implementation

To ensure the successful delivery of the intervention to participants, implementation is a critical aspect. The E-Fit Fidelity Measurement System was newly developed following existing guidelines [50]. The purpose of this system is to measure the degree of fidelity with which the newly developed E-Fit was implemented. Essential components, including specific questions to be asked and required session completion, were organized, phrased and complemented by response choices (yes and no). During the study, the Fidelity Measurement System will be piloted and evaluated.

Practicality

For peer review only

Practicality explores how an intervention can be delivered to intended participants with existing means, resources, and circumstances. It includes factors affecting implementation ease or difficulty, efficiency, speed and quality of implementation, positive or negative effects on target participants, and the participant's ability to complete the intervention activities throughout the intervention period.

Integration

Integration assesses the extent to which the intervention can be integrated within the existing system. This addresses perceived fit with infrastructure and perceived sustainability during the last coaching session.

Limited efficacy

Limited efficacy explores whether the new intervention shows promise of succeeding with the intended population. This is measured by evaluating the program's intended effects or process on key intermediate variables, effect-size estimation, and maintenance of changes from initial change (See table 1 for an overview of EF measures).

2.8 Secondary outcome measures

In addition to primary outcome measures, we will examine other variables assessing the success of the intervention. The following secondary outcome measures are to be collected from both groups at baseline, 2-month, and 6-month follow-up: The Family Relationship Index is a self-report measure used to evaluate subjective quality of family environment for parents [51]. The Kidscreen-10 assesses the child's subjective health and well-being, and the Kidscreen-27 assesses the child's health and well-being from a parent's perspective; these are combined to assess the child's QoL [52]. The parents will complete the short form of the Conners-3 and the Social Responsiveness Scale [53,54] to screen for behavioral problems. The Resilience Scale (RS-13) is a self-reported questionnaire assessing resilience by acceptance of self-, life- and personal competencies. This questionnaire will be completed by the parents and the child [55].

2.9 Covariate measures

Medical, sociodemographic, and patient-specific factors, and IQ will be measured in both groups at baseline and be treated as covariates for the effectiveness of the intervention and to describe the population who participated in the intervention.

Socioeconomic status (SES) will be estimated on a six-point scale for both paternal and maternal education; leading to the lowest possible SES sum score of 2, and the highest of 12 [56]. Cardiac and neonatal data will be collected from medical records. A short form of the Wechsler Intelligence Scale for Children, fifth edition will be used to assess total IQ once at the baseline assessment [57].

For peer review only

2.10 Determination of sample size

This is a proof-of-concept study to test the feasibility of a new intervention. Accordingly, the primary outcome is feasibility, thus no minimum sample size has been calculated [58,59]. A retrospective power analysis will be conducted. Instead, the sample size was defined from previous literature [59,60]. Using a pilot study with a sample size <30 may lead to the potential to underrecruit for the main study [59]. To provide a high level of confidence, a total sample size between 35 and 55 is suggested to account also for small effect sizes. As the total population to recruit from for the present study is relatively small, we decided on a sample size of 40 (E-Fit intervention group n = 20, control group n = 20).

Approximately 210 children are eligible for screening and are to be contacted. In a study by Spillmann et al. [15] using a cut-off of $\geq$ 65 in the BRIEF, around 20% of children with complex CHD had EF difficulties at 10 years. Using data from children and adolescents between 10 and 15 years and a cut-off of $\geq$ 60, we estimate around 30% of eligible children to demonstrate EF difficulties, leading to a potential sample size of 60 children. We assume a response rate of around 75% from those families with children with EF problems, leading to a study sample of approximately 45 participants. A drop-out rate of approximately 10% allows the planned sample of 40 participants to be achieved.

2.11 Data analysis plan

The feasibility of the intervention will be delineated from the descriptive statistics.

Descriptive statistics will be calculated, including means, SD, medians, and IQRs for continuous variables and frequency counts and percentages for categorical variables. The newly developed E-Fit Fidelity Measurement System will be evaluated. Two raters will complete it individually, and inter-rater reliability will be reported. To report limited efficacy, within-subject differences between EF performances from baseline to post-treatment and 6-month follow-up assessments are to be investigated across treatment groups with an intention-to-treat analysis. Linear regression controlling for covariates, involving differences in means and 95% CI, will be used to assess differences between groups and timepoints for continuous outcomes. Proportions and logistic regressions for binary outcomes will be used to examine group effects (e.g., comparison of clinically relevant vs. not clinically relevant scores in the BRIEF, $\chi 2$ tests, ORs, and 95% CI). Given that missing values are missing at random, they will be imputed using multiple imputations.

The data will be visually inspected to identify discordant and influential observations. All analyses will be conducted with an alpha level of 0.05. The statistical software R will be used for all analyses [61]. The final project report will describe and justify any deviations from the original statistical plan.

For peer review only

2.12 Patient and public involvement

As described above (see 2.1), children and adolescents with CHD, their parents, and their teachers were involved in the intervention development and design. Study results will be disseminated to participants and their families through a newsletter and a public informative meeting.

3. Ethics and dissemination

The protocol was approved by the ethical committee of the Canton of Zurich in Switzerland (BASEC-Nr: 2021-02413) and was registered on ClinicalTrials.gov (NCT05198583). Written informed consent will be obtained from the parents. Data handling, record keeping, and archiving will be performed according to the guidelines given by the ethical committee's guidelines (for details see 6_Appendix). The results of this study will be disseminated through presentations at national and international conferences, publications in peer-reviewed journals, and directly to families who participated in the study.

Key outputs from the trial will contribute to our dissemination and impact agenda: Pilot data will inform the decision whether to proceed to a definitive randomized controlled trial and if we do, we will have evidence to support funding applications and a finalized intervention delivery manual to enable multicenter replication of E-Fit.

4. Discussion

This article outlines the development and design of a randomized-controlled feasibility study, investigating an 8-week EF intervention called "E-Fit" for children with CHD. Given the limited effects of previous EF interventions, E-Fit was newly developed based on comprehensive input from patients, families, teachers, and health professionals. It includes a strategy coaching, and both computerized and analog game-based training, and is personalized to the individual participant.

This study evaluates feasibility through the areas of focus acceptability, demand, implementation, practicality, integration and limited efficacy. The results of this study will lay the foundation for a main randomized controlled trial to investigate effectiveness in a larger sample. While E-Fit was initially developed to address EF deficits in children with CHD, successful feasibility validation could also broaden the applicability of E-Fit to other pediatric populations facing EF difficulties.

For peer review only

5. Acknowledgments

The authors thank Nina Zeltner, who supported the qualitative analysis, and Thomas Pletschko, who reviewed the structure and content of the strategy coaching.

Word count main document: 4898

For peer review only

Table 1. Executive function domains

EF domain	Assessment test
Inhibition	Color Word Interference Task (D-KEFS [62])<br>Go/No-Go (TAP [63])
Cognitive Flexibility	Trail-making Test (D-KEFS [62])<br>Color Word Interference Task (D-KEFS [62])
Planning	Tower Test (D-KEFS [62])
Working Memory	Digit Span (WISC-V [57])<br>Picture Span (WISC-V [57])<br>Number Letter Sequencing (WISC-V [57])
Risk-taking	Balloon Analog Risk Task [64]

Abbreviations: D-KEFS, Delis-Kaplan Executive Function System; EF, executive function; WISC -V, Wechsler Intelligence Scale for Children fifth edition

Table 2. Strategy coaching procedure.

	**Strategy coaching procedure**							
	Week 1	***Week 2***	***Week 3***	***Week 4***	***Week 5***	***Week 6***	***Week 7***	***Week 8***
Child		60min individual topic	60min individual topic	60min individual topic	60min individual topic	60min individual topic	60min individual topic	
Parent	30min Call			30min Call				
Parent and Child	60min initialization session							45min Wrap up session

For peer review only

Table 3. Feasibility focus areas and assessment tools

Area of focus	Assessment tool	Group	Timepoint	Completed by
Acceptability	Acceptance and Feasibility scale [65]	E-Fit	Final coaching	Parents and children
	Children's Feeling scale [65]	E-Fit	Intervention period, before and after each online training	Children
	Observed engagement [66]	E-Fit	Each coaching session	Coach
Demand	Acceptance and Feasibility scale	E-Fit	Final coaching	Parents and children
	Fun and Demand [67]	E-Fit	Intervention period, before and after each online training	Children
	Session data: duration, number of completed sessions, and for the computerized training, the coaching, and the card games	E-Fit	Intervention period	
	Recruitment rate	E-Fit	End of study	
Implementation	E-Fit Fidelity Measurement System tool, developed based on Feely et. al [50]	E-Fit	End of study	Two separate raters
Practicality	Acceptance and Feasibility scale [65]	E-Fit	Final coaching	Parents and children
	Feedback in coaching sessions	E-Fit	Each coaching session	Parents and children
	Observed involvement [66]	E-Fit	End of study	
	Session rating scale [68]	E-Fit	Each coaching session	Children
	Observed engagement [66]	E-Fit	Each coaching session	Coaches
	Recruitment rates	Both	End of study	
	Retention rates	Both	End of study	
	Completion of weekly sessions	E-Fit	End of study	
	f events (AEs)	Both	Continuously	
Integration	Acceptance and Feasibility scale	E-Fit	Final coaching	Parents and children
Limited efficacy	Delis-Kaplan Executive Function System [62]	Both	BL, T_1, T_2	
	Wechsler Intelligence Scale for Children- 5th edition [57]	Both	BL, T_1, T_2	
	Test of Attentional Performance [63]	Both	BL, T_1, T_2	
	Balloon Analog Risk Task [64]	Both	BL, T_1, T_2	
	Behavior Rating Inventory for Executive Function [40]	Both	BL, T_1, T_2	Parents* and teachers

*Note: *if possible, completed by both parents separately*

For peer review only

Abbreviations: D-KEFS, Delis-Kaplan Executive Function System; EF, executive function; WISC -V, Wechsler Intelligence Scale for Children fifth edition

Figure legend

Figure 1. Flow chart of intervention development and trial design.

Funding

This work was supported by the Swiss National Science Foundation (SNF) grant number 32003B_172914. This funding source had no role in the design of this study and will not have any role during its execution, analyses, interpretation of data, or decision to submit results.

CogniFit Inc © 2022 had no role in the design of this study and will not have any role during its execution, analyses, interpretation of data, or decision to submit results.

Authors contributions

BL, ME, FW, RO'GT, OK, ML, and AS conceived and designed the study. *BL* is the grant holder. *AS* wrote and revised the manuscript. All authors critically revised the manuscript for important intellectual content.

Competing interests

The authors have no financial or other competing interests for the overall study.

References

1 Bernier P-L, Stefanescu A, Samoukovic G, *et al.* The Challenge of Congenital Heart Disease Worldwide: Epidemiologic and Demographic Facts. *Semin Thorac Cardiovasc Surg Pediatr Card Surg Annu* 2010;**13**:26–34. doi:10.1053/j.pcsu.2010.02.005

2 Wernovsky G, Licht DJ. Neurodevelopmental Outcomes in Children with Congenital Heart Disease – What can we impact? *Pediatr Crit Care Med J Soc Crit Care Med World Fed Pediatr Intensive Crit Care Soc* 2016;**17**:S232–42. doi:10.1097/PCC.0000000000000800

3 Marino BS, Lipkin PH, Newburger JW, *et al.* Neurodevelopmental Outcomes in Children With Congenital Heart Disease: Evaluation and Management. *Circulation* 2012;**126**:1143–72. doi:10.1161/CIR.0b013e318265ee8a

4 Feldmann M, Bataillard C, Ehrler M, *et al.* Cognitive and Executive Function in Congenital Heart Disease: A Meta-analysis. *Pediatrics* 2021;**148**:e2021050875. doi:10.1542/peds.2021-050875

5 Jackson WM, Davis N, Calderon J, *et al.* Executive functions in children with heart disease: a systematic review and meta-analysis. *Cardiol Young* 2021;**31**:1914–22. doi:10.1017/S1047951121001074

6 Diamond A. Executive Functions. *Annu Rev Psychol* 2013;**64**:135–68. doi:10.1146/annurev-psych-113011-143750

7 Lehto JE, Juujärvi P, Kooistra L, *et al.* Dimensions of executive functioning: Evidence from children. *Br J Dev Psychol* 2003;**21**:59–80. doi:10.1348/026151003321164627

8 Miyake A, Friedman NP, Emerson MJ, *et al.* The Unity and Diversity of Executive Functions and Their Contributions to Complex "Frontal Lobe" Tasks: A Latent Variable Analysis. *Cognit Psychol* 2000;**41**:49–100. doi:10.1006/cogp.1999.0734

9 Miyake A, Friedman NP. The Nature and Organization of Individual Differences in Executive Functions: Four General Conclusions. *Curr Dir Psychol Sci* 2012;**21**:8–14. doi:10.1177/0963721411429458

10 Baggetta P, Alexander PA. Conceptualization and Operationalization of Executive Function. *Mind Brain Educ* 2016;**10**:10–33. doi:10.1111/mbe.12100

11 Blair C, Razza RP. Relating Effortful Control, Executive Function, and False Belief Understanding to Emerging Math and Literacy Ability in Kindergarten. *Child Dev* 2007;**78**:647–63. doi:10.1111/j.1467-8624.2007.01019.x

For peer review only - http://bmjopen.bmj.com/site/about/guidelines.xhtml

12 Miller M, Nevado-Montenegro AJ, Hinshaw SP. Childhood Executive Function Continues to Predict Outcomes in Young Adult Females with and Without Childhood-Diagnosed ADHD. *J Abnorm Child Psychol* 2012;**40**:657–68. doi:10.1007/s10802-011-9599-y
13 Taylor Tavares JV, Clark L, Cannon DM, *et al.* Distinct Profiles of Neurocognitive Function in Unmedicated Unipolar Depression and Bipolar II Depression. *Biol Psychiatry* 2007;**62**:917–24. doi:10.1016/j.biopsych.2007.05.034
14 Baler RD, Volkow ND. Drug addiction: the neurobiology of disrupted self-control. *Trends Mol Med* 2006;**12**:559–66. doi:10.1016/j.molmed.2006.10.005
15 Spillmann R, Polentarutti S, Ehrler M, *et al.* Congenital heart disease in school-aged children: Cognition, education, and participation in leisure activities. *Pediatr Res* 2021;:1–7. doi:10.1038/s41390-021-01853-4
16 Sanz JH, Wang J, Berl MM, *et al.* Executive Function and Psychosocial Quality of Life in School Age Children with Congenital Heart Disease. *J Pediatr* 2018;**202**:63–9. doi:10.1016/j.jpeds.2018.07.018
17 Jackson JL, Gerardo GM, Monti JD, *et al.* Executive Function and Internalizing Symptoms in Adolescents and Young Adults With Congenital Heart Disease: The Role of Coping. *J Pediatr Psychol* 2018;**43**:906–15. doi:10.1093/jpepsy/jsx154
18 Calderon J, Bellinger DC. Executive function deficits in congenital heart disease: why is intervention important? *Cardiol Young* 2015;**25**:1238–46. doi:10.1017/S1047951115001134
19 Diamond A. Why improving and assessing executive functions early in life is critical. In: *Executive function in preschool-age children: Integrating measurement, neurodevelopment, and translational research*. Washington, DC, US: : American Psychological Association 2016. 11–43. doi:10.1037/14797-002
20 Calderon J, Wypij D, Rofeberg V, *et al.* Randomized Controlled Trial of Working Memory Intervention in Congenital Heart Disease. *J Pediatr* 2020;**227**:191-198.e3. doi:10.1016/j.jpeds.2020.08.038
21 Jordan LC, Siciliano RE, Cole DA, *et al.* Cognitive training in children with hypoplastic left heart syndrome: A pilot randomized trial. *Prog Pediatr Cardiol* 2020;**57**:101185. doi:10.1016/j.ppedcard.2019.101185
22 Cogmed. https://www.cogmed.com/training (accessed 19 Jan 2023).
23 Calderon J, Wypij D, Rofeberg V, *et al.* Randomized Controlled Trial of Working Memory Intervention in Congenital Heart Disease. *J Pediatr* 2020;**227**:191-198.e3. doi:10.1016/j.jpeds.2020.08.038
24 Takacs ZK, Kassai R. The efficacy of different interventions to foster children's executive function skills: A series of meta-analyses. *Psychol Bull* 20190429;**145**:653. doi:10.1037/bul0000195
25 Ehrler M, Naef N, Tuura RO, *et al.* Executive function and brain development in adolescents with severe congenital heart disease (Teen Heart Study): protocol of a prospective cohort study. *BMJ Open* 2019;**9**:e032363. doi:10.1136/bmjopen-2019-032363
26 Kukartz U. *Qualitative Inhaltsanalyse. Methoden, Praxis, Computerunterstützung*. 4. überarbeitete Auflage. 2018.
27 Gordon T, Helmer O. REPORT ON A LONG-RANGE FORECASTING STUDY. 1964. https://apps.dtic.mil/sti/citations/AD0607777 (accessed 23 Jan 2023).
28 Hasson F, Keeney S, McKenna H. Research guidelines for the Delphi survey technique. 2000;**32**:1008–15.
29 Twiss BC. The Delphi method—Techniques & applications: edited by H. A. Linstone and M. Turoff, Addison-Wesley, Reading, Mass. (1975), 620 pp. $29.50 (hardback) and $16.50 (softback), also by West End House, 11 Hill's Place, London. *Long Range Plann* 1976;**9**:94–5. doi:10.1016/0024-6301(76)90044-3
30 Scheibe M, Skutsch M, Schofer J. *The Delphi Method: Techniques and Applications*. London Reading, Mass.: Addison-Wesley 1975.
31 Drzewiecki CM, Juraska JM. The structural reorganization of the prefrontal cortex during adolescence as a framework for vulnerability to the environment. *Pharmacol Biochem Behav* 2020;**199**:173044. doi:10.1016/j.pbb.2020.173044
32 Teffer K, Semendeferi K. Chapter 9 - Human prefrontal cortex: Evolution, development, and pathology. In: Hofman MA, Falk D, eds. *Progress in Brain Research*. Elsevier 2012. 191–218. doi:10.1016/B978-0-444-53860-4.00009-X
33 Long SH, Eldridge BJ, Harris SR, *et al.* Challenges in Trying to Implement an Early Intervention Program for Infants with Congenital Heart Disease. *Pediatr Phys Ther* 2015;**27**:38–43. doi:10.1097/PEP.0000000000000101
34 Cognifit. Cognifit Inc © 2022. 2022.
35 Dawson P, Guare R. *Schlau aber...* 1st ed. Bern: : Verlag Hans Huber, Hogrefe AG 2012.
36 Liebers A, Kubesch S. *Ein Ritter in der Klasse*. 1st ed. Verlag Bildung Plus 2013.
37 Liebers A, Kubesch S. *Mathetest und Drachenhörnchen*. 1st ed. Verlag Bildung Plus 2013.
38 Liebers A, Kubesch S. *Ob Gespenter Fußball spielen?* 1st ed. Verlag Bildung Plus 2014.
39 Liebers A, Kubesch S. *Stopp oder es kracht!* Verlag Bildung Plus 2014.
40 Gioia GA, Isquith PK, Guy SC, *et al.* Behavior Rating Inventory of Executive Function®, Second Edition (BRIEF®2). 2000;:2.
41 R Core Team. R: A language and environment for statistical computing. Vienna, Austria: 2020. https://www.R-project.org/
42 Wright A. REDCap: A Tool for the Electronic Capture of Research Data. *J Electron Resour Med Libr* 2016;**13**:197–201. doi:10.1080/15424065.2016.1259026
43 Bustamante JC, Navarro J-J. Technological Tools for the Intervention and Computerized Dynamic Assessment of Executive Functions. Handb. Res. Neurocognitive Dev. Exec. Funct. Implic. Interv. 2022;:310–39. doi:10.4018/978-

For peer review only - http://bmjopen.bmj.com/site/about/guidelines.xhtml

1-7998-9075-1.ch014
44 Rachanioti E, Bratitsis T, Alevriadou A. Cognitive games for children's Executive Functions Training with or without learning difficulties: an Overview. In: *Proceedings of the 8th International Conference on Software Development and Technologies for Enhancing Accessibility and Fighting Info-exclusion*. Thessaloniki Greece: : ACM 2018. 165–71. doi:10.1145/3218585.3218665
45 Horowitz-Kraus T. Differential Effect of Cognitive Training on Executive Functions and Reading Abilities in Children With ADHD and in Children With ADHD Comorbid With Reading Difficulties. *J Atten Disord* 2015;**19**:515–26. doi:10.1177/1087054713502079
46 Leiss U. Die neuropsychologische Ergebnisbesprechung als Intervention – Eine Brücke zwischen Befund und Alltag. In: Pletschko T, Leiss U, Pal-Handl K, *et al.*, eds. *Neuropsychologische Therapie mit Kindern und Jugendlichen: Praktische Behandlungskonzepte bei neurokognitiven Funktionsstörungen*. Berlin, Heidelberg: : Springer 2020. 35–43. doi:10.1007/978-3-662-59288-5_4
47 Dawson P, Guare R. *Smart But Scattered: The Revolutionary "executive Skills" Approach to Helping Kids Reach Their Potential*. Guilford Press 2009.
48 Pletschko T, Leiss U, Pal-Handl K, *et al. Neuropsychologische Therapie mit Kindern und Jugendlichen: Praktische Behandlungskonzepte bei neurokognitiven Funktionsstörungen*. Springer-Verlag 2020.
49 Bowen DJ, Kreuter M, Spring B, *et al.* How We Design Feasibility Studies. *Am J Prev Med* 2009;**36**:452–7. doi:10.1016/j.amepre.2009.02.002
50 Feely M, Seay KD, Lanier P, *et al.* Measuring Fidelity in Research Studies: A Field Guide to Developing a Comprehensive Fidelity Measurement System. *Child Adolesc Soc Work J* 2018;**35**:139–52. doi:10.1007/s10560-017-0512-6
51 Hoge RD, Andrews DA, Faulkner P, *et al.* The family relationship index: Validity data. *J Clin Psychol* 1989;**45**:897–903. doi:10.1002/1097-4679(198911)45:6<897::AID-JCLP2270450611>3.0.CO;2-T
52 Ravens-Sieberer U. The KIDSCREEN questionnaires. Quality of life quiestionners for children and adolescents - handbook. 2006.
53 Lidzba K, Chrstiansen H, Drechsler R. Conners Skalen zu Aufmerksamkeit und Verhalten - 3. Deutschsprachige Adaptation der Conners 3rd Edition® (Conners 3®) von C. Keith Conners. 2013.
54 Bölte S, Poustka F. Skala zur Erfassung sozialer Reaktivität - Dimensionale Autismus-Diagnostik. Deutsche Fassung der Scoial Responsiveness Scale (SRS) von John N. Constantino und Christian P. Gruber. 2007.
55 Leppert K, Koch B, Brähler E, *et al.* Resilience scale - evaluation of a long (RS-25) and a short version (RS-13). *Klin Diagn Eval* 2008.
56 Largo RH, Pfister D, Molinari L, *et al.* Significance of prenatal, perinatal and postnatal factors in the development of AGA preterm infants at five to seven years. *Dev Med Child Neurol* 1989;**31**:440–56.
57 Wechsler D. Study Citation Wechsler, D. (2014). WISC-V: Technical and Interpretive Manual. Bloomington. 2014.
58 Eldridge SM, Lancaster GA, Campbell MJ, *et al.* Defining Feasibility and Pilot Studies in Preparation for Randomised Controlled Trials: Development of a Conceptual Framework. *PLOS ONE* 2016;**11**:e0150205. doi:10.1371/journal.pone.0150205
59 Sim J, Lewis M. The size of a pilot study for a clinical trial should be calculated in relation to considerations of precision and efficiency. *J Clin Epidemiol* 2012;**65**:301–8. doi:10.1016/j.jclinepi.2011.07.011
60 Billingham SA, Whitehead AL, Julious SA. An audit of sample sizes for pilot and feasibility trials being undertaken in the United Kingdom registered in the United Kingdom Clinical Research Network database. *BMC Med Res Methodol* 2013;**13**:104. doi:10.1186/1471-2288-13-104
61 RStudio Team. RStudio: Integrated Development Environment for R. 2021.http://www.rstudio.com/
62 Delis DC, Kaplan E, Kramer JH. Delis-Kaplan Executive Function System. APA PsycNet. 2001.https://psycnet.apa.org/doiLanding?doi=10.1037%2Ft15082-000 (accessed 19 Jan 2022).
63 Zimmermann P, Fimm B. *Applied Neuropsychology of Attention: Theory, Diagnosis and Rehabilitation*. Psychology Press 2004.
64 Lejuez CW, Aklin W, Daughters S, *et al.* Reliability and Validity of the Youth Version of the Balloon Analogue Risk Task (BART–Y) in the Assessment of Risk-Taking Behavior Among Inner-City Adolescents. *J Clin Child Adolesc Psychol* 2007;**36**:106–11. doi:10.1080/15374410709336573
65 Modi AC, Gutierrez-Colina AM, Wagner JL, *et al.* Executive functioning phenotypes in youth with epilepsy. *Epilepsy Behav* 2019;**90**:112–8. doi:10.1016/j.yebeh.2018.11.026
66 Reeve J, Jang H, Carrell D, *et al.* Enhancing Students' Engagement by Increasing Teachers' Autonomy Support. *Motiv Emot* 2004;**28**:147–69. doi:10.1023/B:MOEM.0000032312.95499.6f
67 Vazou S, Klesel B, Lakes KD, *et al.* Rhythmic Physical Activity Intervention: Exploring Feasibility and Effectiveness in Improving Motor and Executive Function Skills in Children. *Front Psychol* 2020;**11**.https://www.frontiersin.org/article/10.3389/fpsyg.2020.556249 (accessed 19 Jan 2022).
68 Duncan B, Miller S, Sparks J, *et al.* The Session Rating Scale: Preliminary Psychometric Properties of a "Working" Alliance Measure. *J Brief Ther* 2003;**3**:3–12.

For peer review only - http://bmjopen.bmj.com/site/about/guidelines.xhtml

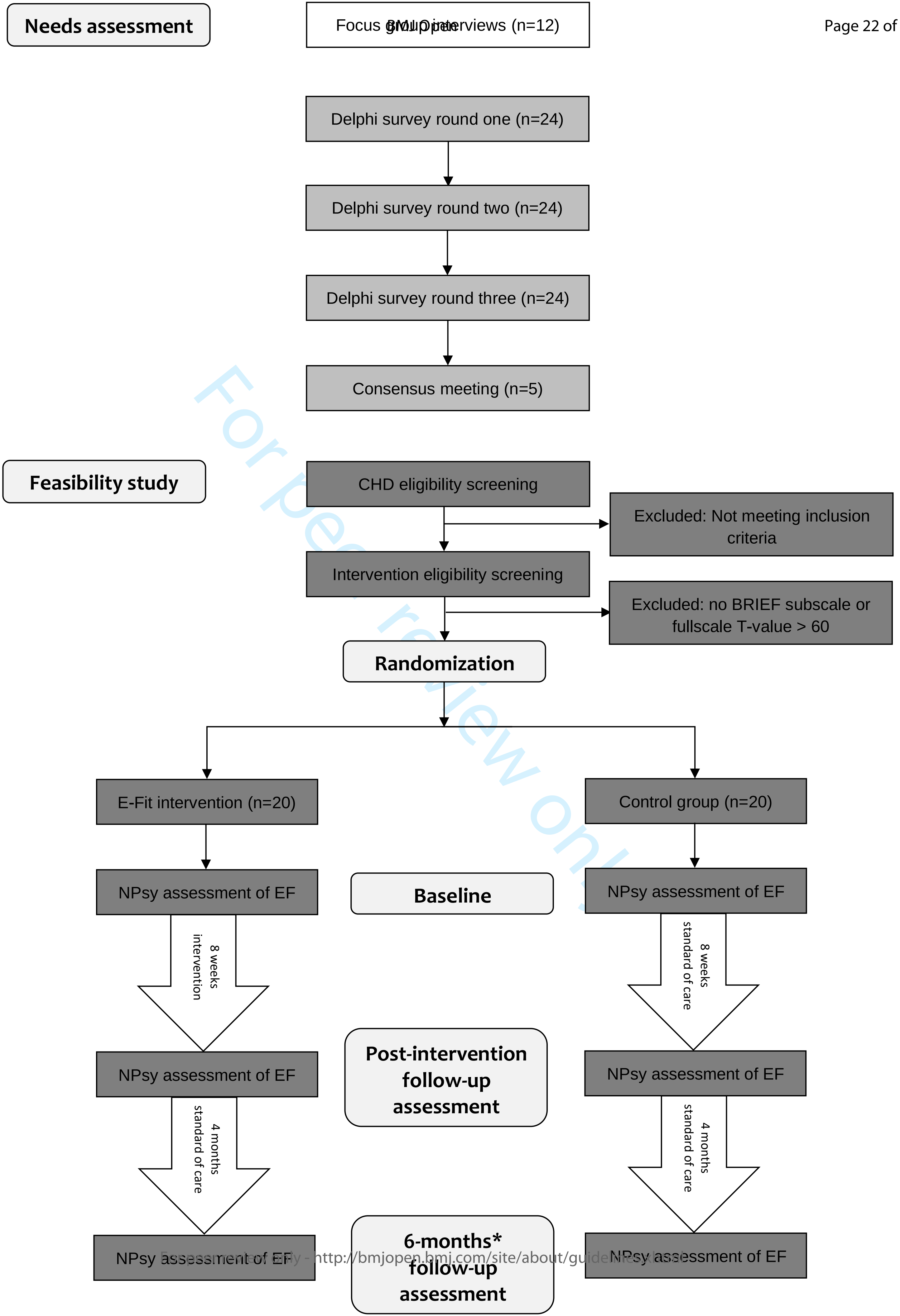

For peer review only
For peer review only - http://bmjopen.bmj.com/site/about/guidelines.xhtml

UNIVERSITY
CHILDREN'S HOSPITAL
ZURICH

The hospital of the Eleonore Foundation

Focus group November

Discussion			
**Target group**	☐ Parents ☒ Children		
**Date / time / place**	23.11.2020	18.00-19.00	Zoom
**Lead**	Alenka Schmid, Interviewer 2, Interviewer 3		
**Topic**	☒ Intervention		
**Materials / Tools**	• Dodelido (Spiel)<br>• Matches	•	•
**Preparations**	Before:<br>• PowerPoint slides<br>• Create evaluation on Google Docs<br>• Create Collaboard Account (Interviewers: https://web.collaboard.app/projects)<br>• Ensure good lighting	During:<br>• Create Breakoutrooms	

Time	Description	Moderator
18.00-18.10	**Introduction**<br><br>Short greeting, introduction (first names), arriving and rename in Zoom<br>Explain why they are here today<br>Gallery view, mute when not speaking<br>Raise hand<br>Introductions (which animal would I like to be?)	Alenka
18.10-18.20<br><br>18:20-18:30<br><br>18:30-18:40	**Main part**<br><br>Introduction and info about brain training for EF and discussion framework via PowerPoint:<br><br>Different scenarios are presented (shopping and then forgetting, etc.). Children can stretch out if this has happened to them before.<br><br>In a second step, they are asked to stretch out where they feel it happens to them more often than others.<br><br>Possible scenarios<br>- Anger/emotion control<br>- Planning/remembering (https://www.youtube.com/watch?v=o4zbeBclAtU)<br>- Distractedness (https://www.youtube.com/watch?v=xhHkk1q04GA)<br>- Forgetfulness (https://www.youtube.com/watch?v=9TfJYgnXSus)<br><br>Presenting different possibilities<br>There is something different in each break-out room. Afterwards, the participants are mixed together so that someone from each group joins for the Disney method.<br>These are the following items:<br><br>1. Rubiks Cage (watch video, game with matches)	Alenka<br><br>Alenka<br><br>Interviewer 2

For peer review only - http://bmjopen.bmj.com/site/about/guidelines.xhtml

UNIVERSITY CHILDREN'S HOSPITAL ZURICH

The hospital of the Eleonore Foundation

<table>
<tr><td>

18:40-
19:00

19:00</td><td>2. Cogmed (https://www.pearsonclinical.co.uk/Education/BestsellingInterventions/Cogmed/CogmedWorkingMemoryTraining.aspx; jeder für sich)
3. Dodelido

Disney Method (Breakout Rooms)
<table>
<tr><td>Dreamer</td><td>Participants are allowed to share and discuss their craziest ideas</td><td>5min</td></tr>
<tr><td>Critic</td><td>The participants reflect on what is feasible from their crazy ideas.</td><td>5min</td></tr>
<tr><td>Realist</td><td>The participants try to adapt their ideas so that they still have something to do with their dreams, but are more feasible from their point of view. Here they are also welcome to bring in their experiences from the previous breakout rooms.</td><td>10min</td></tr>
</table>
Return from Breakout rooms</td><td>Alenka

Interviewer 3

Interviewer 3, Interviewer 2</td></tr>
<tr><td>19:00-
19:05</td><td>Closing

Feedback (fill in online) and acknowledgement

Questions?</td><td>Alenka</td></tr>
</table>

Goal of discussion?

	• Needs assessment
**Safety considerations**	• The event will be conducted online via Zoom

Details:

https://www.youtube.com/watch?v=xlqUjpPxcTk
Keep matches ready
Google form for evaluation:

- *In what time frame should the training take place (2 weeks/3 months)?*
- *How much time can the child (and parents) invest each day?*
- *Where should the training take place (at home, at school, at KiSpi, alone, in a group, with parents)?*
- *By what media (paper tasks, computer games, board games)?*
- *How much can or do you want parents to be involved in the training?*

For peer review only

For peer review only - http://bmjopen.bmj.com/site/about/guidelines.xhtml

UNIVERSITY
CHILDREN'S HOSPITAL
ZURICH

The hospital of the Eleonore Foundation

Focus group November

Discussion			
**Target group**	☒ Parents ☐ Children		
**Date / time / place**	23.11.2020	20.00-21.00	Zoom
**Lead**	Bea Latal, Alenka Schmid		
**Topic**	☒ Intervention		
**Materials / Tools**	• Dodelido<br>• Matches	•	•
**Preparations**	Before:<br>• PowerPoint slides	During:	

Time	Description	Moderator
	**Introduction**	
20.00-20.05	Short greeting, arriving (and rename on Zoom).	Bea
	Introductions (name and name of child, favorite vacation spot), write name and corona front to back.	Alenka
20:05-20:15	Participants are given the link to a Google Survey (or Wordcloud) via Zoom Chat. In it, they can openly write down their thoughts on various topics.<br>Topics:<br>Expectations for tonight<br>Particularly positive characteristics of your child?<br>Everyday problems of the child and with the child?<br>Wishes for further development of the child?	Bea
	**Main part**	
20.15-20.25	Introduction and information about EF at CHD and discussing framework via PowerPoint.	Alenka
20:25-20:35	Introductory question:<br>- Do you feel like a training of the executive functions could help your child?	
	Afterwards, the discussion is let run its course and moderated, if necessary, further questions are introduced.<br>Possible questions:<br><br>- What problems does your child have in everyday life?<br>- What do you expect from the training?<br>- What do you expect from the people who offer and conduct training?<br>- In what time frame should a training take place. How much time can the child and parents invest?<br>- At what age should it start?<br>- Where should the training take place, by what media? Paper tasks, computer games, alone, in group, at home, at the children's hospital?<br>- How much can or do you as parents want to participate in the training?<br>- Do you think your child would be motivated to do such a training?<br>- Are there other things for which training/intervention would be useful? (E.g., stress management, social graces, understanding the illness, exercise and physical activity?)	Bea

For peer review only - http://bmjopen.bmj.com/site/about/guidelines.xhtml

UNIVERSITY
CHILDREN'S HOSPITAL
ZURICH

The hospital of the
Eleonore Foundation

20:35-20:55	Presentation of the team's ideas or direct demonstration (of the same things as to the children) and discussion of them	Alenka
20:55-21:00	**Closing**<br>Google Doc for evaluation<br>Acknowledgement and closing	Alenka<br>Bea

Goal of discussion?

	• Needs assessment
**Safety considerations**	• The event will be conducted online via Zoom

For peer review only

For peer review only - http://bmjopen.bmj.com/site/about/guidelines.xhtml

A) Development of the Delphi survey

Sentences derived by qualitative content analysis from the focus groups were sorted into nine categories. Then, the statements were summarized and finally used to define the items in the Delphi survey. An example of statements and the consequent items is given in table S6. The items are deliberately positively formulated. Some items were highly specific to children with CHD. Hence, the items for the “identity” and “dealing with the disease” categories were dropped for the Delphi survey. The final set of items included in the Delphi survey is provided in figure S1.

B) Rationale behind the final intervention design

B.1) Virtual and analog games

Another element key to the success of any intervention is motivation [1]. Johann and Karbach compared six computer-based cognitive training programs [2]. They found evidence that playful interventions promote motivation and adherence in typically developing elementary school students. This reinforced the effects achieved by the EF training [2]. In addition, they emphasize the difference between computer-based training with added game elements and regular video games [3]: Videogames such as Super Mario and Fruit Ninja have no effect on EFs. In contrast, computer-based training with added game elements, such as in Lumosity® or CogniFit Inc © 2022 [4,5] shows clear benefits for EF improvement. In computer-based approaches, the training effect is maintained, and motivation is increased [3]. Because an intervention should be enjoyable to keep participants motivated, analog games such as board and card games have also been tested as cognitive training [6,7]. One obstacle associated with interventions using analog games is the mandatory involvement of other family or study members, which might overstrain time resources. However, participants of both the focus group and the Delphi survey indicated that it would be important to include analog games in the intervention. Hence, different analog games were added to the intervention, including games that can be played alone, and this part was decided to be open in terms of frequency and time spent on it. Moreover, whether the intervention should be online or analog was discussed without reaching a consensus by the Delphi survey participants. A systematic review and meta-analysis by Cao et al. found slight-to-moderate evidence for the effectiveness of computer-based interventions in promoting EF [3]. Computer-based interventions have been used in the population of CHD patients previously, and in the Delphi survey, only 4 of 12 participants disagreed with the online format. Hence, we decided that the intervention should include computer-based games. In addition, participating children could also use analog games. We decided to use existing computer-based and analog games instead of creating new games.

The intervention also includes analog and computerized games which, in addition to EF, promote eye-hand coordination, thus meeting a suggestion that motor games be included.

For peer review only - http://bmjopen.bmj.com/site/about/guidelines.xhtml

B.2) Strategy Coaching

The most highly rated items were related to learning strategies addressing everyday lives and homework. This is in line with the literature: another substantial shortcoming of many interventional studies is the lack of transfer [8–11]. Transfer refers to the degree of improvement in specific tasks requiring the same cognitive functions, termed near transfer, and in general cognitive abilities, termed far transfer [12]. A way to overcome a lack of transfer is by providing personal coaching sessions that support EF strategies in daily life and take into account children's emotional and social development [8]. Hence, we decided on adding strategy coaching. Career planning was a topic discussed in the Delphi survey. It was decided not to include this topic, as this is not appropriate for an age group of 10–12-year-old children.

B.3) Length, frequency per week and overall duration of the intervention

The most difficult aspect of the development of the intervention related to the minutes per day, frequency per week, and overall duration of the sessions. A wide range of possibilities was suggested (see figure S2). These could not all be met. The length of the computerized training sessions was defined as 20 minutes, which was in agreement with the participants' suggestions. Other studies using session lengths of 20 minutes trained three to four times a week [13,14], which was a higher frequency than suggested by the families. Consequently, we decided on a frequency of three times a week and an overall duration of 8 weeks instead of the 6 months suggested (figure S2).

For peer review only

1 Mohammed S, Flores L, Deveau J, *et al.* The Benefits and Challenges of Implementing Motivational Features to Boost Cognitive Training Outcome. *J Cogn Enhanc* 2017;**1**:491–507. doi:10.1007/s41465-017-0047-y
2 Johann VE, Karbach J. Effects of game-based and standard executive control training on cognitive and academic abilities in elementary school children. *Developmental Science* 2020;**23**:e12866. doi:10.1111/desc.12866
3 Cao Y, Huang T, Huang J, *et al.* Effects and Moderators of Computer-Based Training on Children's Executive Functions: A Systematic Review and Meta-Analysis. *Front Psychol* 2020;**11**:580329. doi:10.3389/fpsyg.2020.580329
4 Lumos Labs, Inc. Lumosity ®. 2022.
5 Cognifit. Cognifit Inc © 2022. 2022.
6 Estrada-Plana V, Esquerda M, Mangues R, *et al.* A Pilot Study of the Efficacy of a Cognitive Training Based on Board Games in Children with Attention-Deficit/Hyperactivity Disorder: A Randomized Controlled Trial. *Games for Health Journal* 2019;**8**:265–74. doi:10.1089/g4h.2018.0051
7 Gonçalves PD, Ometto M, Sendoya G, *et al.* Neuropsychological Rehabilitation of Executive Functions: Challenges and Perspectives. *Journal of Behavioral and Brain Science* 2014;**2014**. doi:10.4236/jbbs.2014.41004
8 Diamond A, Lee K. Interventions Shown to Aid Executive Function Development in Children 4 to 12 Years Old. *Science* Published Online First: 19 August 2011. doi:10.1126/science.1204529
9 Thorell LB, Lindqvist S, Bergman Nutley S, *et al.* Training and transfer effects of executive functions in preschool children. *Developmental Science* 2009;**12**:106–13. doi:10.1111/j.1467-7687.2008.00745.x
10 Diamond A, Ling DS. Conclusions about interventions, programs, and approaches for improving executive functions that appear justified and those that, despite much hype, do not. *Developmental Cognitive Neuroscience* 2016;**18**:34–48. doi:10.1016/j.dcn.2015.11.005
11 Gunzenhauser C, Nückles M. Training Executive Functions to Improve Academic Achievement: Tackling Avenues to Far Transfer. *Frontiers in Psychology* 2021;**12**.https://www.frontiersin.org/article/10.3389/fpsyg.2021.624008 (accessed 11 Mar 2022).
12 Barnett SM, Ceci SJ. When and where do we apply what we learn?: A taxonomy for far transfer. *Psychological Bulletin* 2002;**128**:612–37. doi:10.1037/0033-2909.128.4.612
13 Greco G. MULTILATERAL TRAINING USING PHYSICAL ACTIVITY AND SOCIAL GAMES IMPROVES MOTOR SKILLS AND EXECUTIVE FUNCTION IN CHILDREN WITH AUTISM SPECTRUM DISORDER. *European Journal of Special Education Research* Published Online First: 16 March 2020. doi:10.46827/ejse.v0i0.2928
14 Ramezan Saatchi L, Khodabakhsh Pirkalani R, Moradisabzevar M, *et al.* The effect of computerized Cognitive Training package on executive functions of people with major depressive disorder. *Iranian Journal of Psychiatric Nursing* 2022;**10**:39–52. doi:10.22034/IJPN.10.3.39

For peer review only

UNIVERSITÄTS-KINDERSPITAL ZÜRICH
Forschungszentrum für das Kind FZK

Einwilligungserklärung

Schriftliche Einwilligungserklärung zur Teilnahme an einer klinischen Studie
Bitte lesen Sie dieses Formular sorgfältig durch. Bitte fragen Sie, wenn Sie etwas nicht verstehen oder wissen möchten. Für die Teilnahme ist Ihre schriftliche Einwilligung notwendig.

**BASEC-Nummer (nach Einreichung):**	
**Titel der Studie (wissenschaftlich und Laiensprache)**:	Executive functions intervention in children and adolescents with severe congenital heart disease: a feasibility study (E-Fit)<br><br>Intervention bei Kindern und Jugendlichen mit schweren angeborenen Herzfehlern: eine Machbarkeitsstudie (E-Fit)
**Verantwortliche Institution (Sponsor mit Adresse)**:	Prof. Dr. med. Bea Latal<br>Abteilung für Entwicklungspädiatrie<br>Universitäts-Kinderspital Zürich<br>Steinwiesstrasse 75, 8032 Zürich
**Ort der Durchführung**:	Universitäts-Kinderspital Zürich
**Prüfärztin/Prüfarzt am Studienort:**<br>Name und Vorname in Druckbuchstaben:	Prof. Dr. med. Bea Latal
**Teilnehmerin/Teilnehmer:**<br>Name und Vorname in Druckbuchstaben:<br>Geburtsdatum:	____________________<br>____________________<br>☐ weiblich ☐ männlich

- Ich und mein Kind wurden von der unterzeichnenden Studienkoordination mündlich und schriftlich über den Zweck, den Ablauf der Studie mit E-Fit über mögliche Vor- und Nachteile sowie über eventuelle Risiken informiert.
- Ich und mein Kind nehmen an dieser Studie freiwillig teil und akzeptiere den Inhalt der mir ausgehändigten schriftlichen Information. Ich und mein Kind hatten genügend Zeit, unsere Entscheidung zu treffen.
- Meine Fragen und die meines Kindes im Zusammenhang mit der Teilnahme an dieser Studie sind uns beantwortet worden. Ich und mein Kind behalten die schriftliche Information und erhalten eine Kopie unserer schriftlichen Einwilligungserklärung.
- Ich und mein Kind sind einverstanden, dass die Kinderärztin/der Kinderarzt meines Kindes über die Teilnahme an der Studie informiert wird.
- Ich und mein Kind sind einverstanden, dass die zuständigen Fachleute des Sponsors, der zuständigen Ethikkommission zu Prüf- und Kontrollzwecken in unsere unverschlüsselten Daten Einsicht nehmen dürfen, jedoch unter strikter Einhaltung der Vertraulichkeit.
- Bei Ergebnissen (und/oder Zufallsbefunden), die direkt die Gesundheit meines Kindes betreffen, werden ich und mein Kind informiert. Wenn ich und mein Kind das nicht wünschen, informieren wir die Studienkoordination.
- Ich und mein Kind wissen, dass die gesundheitsbezogenen und persönlichen Daten meines Kindes nur in verschlüsselter Form zu Forschungszwecken für diese Studie (auch im Ausland)

For peer review only

For peer review only - http://bmjopen.bmj.com/site/about/guidelines.xhtml

weitergegeben werden können. Der Sponsor gewährleistet, dass der Datenschutz nach Schweizer Standard eingehalten wird.

- Ich und mein Kind können jederzeit und ohne Angabe von Gründen von der Studienteilnahme zurücktreten. Die weitere medizinische Behandlung meines Kindes ist unabhängig von der Studienteilnahme gewährleistet. Die bis zum Rücktritt erhobenen Daten werden noch im Rahmen der Studie ausgewertet.
- Die Haftpflichtversicherung Universitäts-Kinderspital Zürich kommt für allfällige Schäden auf.
- Bei Zufallsbefunden möchten wir

  a) ☐ in jedem Fall informiert werden
  b) ☐ nicht informiert werden
  c) ☐ die Entscheidung folgender Person überlassen: ....

- Ich und mein Kind sind damit einverstanden, dass die Studienleitenden den aktuellsten kardiologischen Befund beim/bei der zuständigen Kardiolog:in einholen, um den Gesundheitszustand meines Kindes einzuschätzen.

  Name Kardiologe:___________________________

  Name Klinik: _______________________________

- Ich bin damit einverstanden, dass persönliche Daten von mir im Rahmen dieser Studie ausgewertet und verschlüsselt and Drittpersonen innerhalb des Universitäts-Kinderspitals Zürich weitergegeben werden können.
  ☐ Ja ☐ Nein

- Ich und mein Kind sind damit einverstanden, dass persönliche Daten verschlüsselt an Drittpersonen innerhalb des Universitäts-Kinderspitals Zürich weitergegeben werden können.
  ☐ Ja ☐ Nein

- Ich und mein Kind sind damit einverstanden, dass die Daten aus der neuropsychologischen Abklärung verschlüsselt zu Forschungszwecken an CogniFit© weitergegeben werden können.
  ☐ Ja ☐ Nein

- Ich und mein Kind sind damit einverstanden, dass während der neuropsychologischen Abklärung und der Intervention Videoaufnahmen gemacht und verschlüsselt abgespeichert werden.
  ☐ Ja ☐ Nein

- Ich und mein Kind sind damit einverstanden, dass die Hauptlehrperson über die Studienteilnahme informiert und miteinbezogen wird.
  ☐ Ja ☐ Nein

For peer review only - http://bmjopen.bmj.com/site/about/guidelines.xhtml

TEEN HEART STUDY E-FIT

UNIVERSITÄTS-KINDERSPITAL ZÜRICH
Forschungszentrum für das Kind FZK

Die unterzeichnenden Personen bezeugen, dass das Aufklärungsgespräch stattgefunden hat und dass das urteilsfähige Kind in die Studienteilnahme eingewilligt hat und/oder sich keine Anzeichen von Widerstand gegen die Teilnahme erkennen lassen.

Ort, Datum	Unterschrift gesetzliche Vertretung des Kindes

Bestätigung der Studienkoordination: Hiermit bestätige ich, dass ich dieser Teilnehmerin/diesem Teilnehmer Wesen, Bedeutung und Tragweite der Studie erläutert habe. Ich versichere, alle im Zusammenhang mit dieser Studie stehenden Verpflichtungen gemäss in der Schweiz geltenden Rechts zu erfüllen. Sollte ich im Verlauf der Studie von Aspekten erfahren, welche die Bereitschaft der Teilnehmerin/des Teilnehmers zur Studienteilnahme beeinflussen könnten, werde ich sie/ihn umgehend darüber informieren.

Ort, Datum	Name und Vorname der Studienkoordination in Druckbuchstaben<br><br>Unterschrift der Studienkoordination

For peer review only - http://bmjopen.bmj.com/site/about/guidelines.xhtml

A) Computerized training (CogniFit Inc © 2022)

It is important to note that a contract was concluded with CogniFit Inc © 2022, because they provide discounts on their services in exchange for the pseudonymized baseline assessment data. Families had the choice whether to provide their data or not.

B) Strategy coaching

At the beginning of the intervention, parents complete a short questionnaire [1], assessing the behavior of their child in everyday life. The questionnaire was designed for parents to identify EF domains in which their children face difficulties. Lower scores indicate greater difficulties. Out of eleven EF domains rated, the six lowest scored are selected for the coaching sessions. Possible topics are working memory, attention control, emotion regulation, flexibility, initiating action, metacognition, organization, planning, response inhibition, time management and goal-directed persistence. In the first coaching session, the child's performance in the EF baseline assessment are discussed, and strengths and difficulties are identified [2]. Each of the next six sessions focuses on one of the six EF domains identified as most affected [1]. At the beginning of each session, the previous week is reviewed, focusing on the EF intervention and coaching instructions given at the previous session. Also, difficulties in regard to adherence are discussed and solutions suggested by the coach. In the second part of the session, a short story is read, featuring protagonists dealing with similar challenge as is the topic of that coaching session [3–6]. In the third part of the session, the focus is transferred to the child's situation by asking about how they could handle similar situations. Further, the child completes a task for the coaching session. Templates for these are available in Dawson et al.'s book *Smart but scattered* [1] and include checklist, plans, and tables. At the end of the session, the child is encouraged to use these strategies the following week, and the parents are informed by email about these strategies after the session. The parents receive fact sheets regarding the discussed EF domain of that particular session with tips for everyday life and a short summary on what was done in the session. All parental tips are also taken from the German version of *Smart but scattered* [1].

For peer review only - http://bmjopen.bmj.com/site/about/guidelines.xhtml

1 Dawson P, Guare R. *Schlau aber...* 1st ed. Bern: : Verlag Hans Huber, Hogrefe AG 2012.
2 Leiss U. Die neuropsychologische Ergebnisbesprechung als Intervention – Eine Brücke zwischen Befund und Alltag. In: Pletschko T, Leiss U, Pal-Handl K, *et al.*, eds. *Neuropsychologische Therapie mit Kindern und Jugendlichen: Praktische Behandlungskonzepte bei neurokognitiven Funktionsstörungen*. Berlin, Heidelberg: : Springer 2020. 35–43. doi:10.1007/978-3-662-59288-5_4
3 Liebers A, Kubesch S. *Ob Gespenter Fußball spielen?* 1st ed. Verlag Bildung Plus 2014.
4 Liebers A, Kubesch S. *Mathetest und Drachenhörnchen*. 1st ed. Verlag Bildung Plus 2013.
5 Liebers A, Kubesch S. *Ein Ritter in der Klasse*. 1st ed. Verlag Bildung Plus 2013.
6 Liebers A, Kubesch S. *Stopp oder es kracht!* Verlag Bildung Plus 2014.

For peer review only

TEEN HEART STUDY
E-FIT

Acceptance and Feasibility Scale Parents

	Fully disagree			Fully agree
The program was useful for my child.	☐	☐	☐	☐
My child would do the program again.	☐	☐	☐	☐
I would recommend this program to other children.	☐	☐	☐	☐
My child has applied the skills learned.	☐	☐	☐	☐
My child has a plan for dealing with future problems.	☐	☐	☐	☐
My child has made positive changes.	☐	☐	☐	☐
My child has used the skills to become more organized.	☐	☐	☐	☐
My child has used reminder strategies to remember things more often.	☐	☐	☐	☐
My child uses techniques to plan.	☐	☐	☐	☐
My child has met his or her program goals.	☐	☐	☐	☐
My child has a more positive attitude than before.	☐	☐	☐	☐
My child is better at solving problems.	☐	☐	☐	☐
My child remembers things better.	☐	☐	☐	☐
My child is more organized.	☐	☐	☐	☐
My child is better at paying attention and remembering things.	☐	☐	☐	☐
My child is better at getting things done, such as homework or chores.	☐	☐	☐	☐
My child is better at controlling his or her emotions.	☐	☐	☐	☐
My child is better at controlling his or her actions.	☐	☐	☐	☐
My child is better at stopping and thinking before acting.	☐	☐	☐	☐
My child is better able to handle unforeseen changes in plans.	☐	☐	☐	☐
My child feels less stressed.	☐	☐	☐	☐
My child does better in school.	☐	☐	☐	☐

For peer review only - http://bmjopen.bmj.com/site/about/guidelines.xhtml
adapted after Modi et al., 2019

TEEN HEART STUDY E-FIT

Acceptance and Feasibility Scale Children

	Fully disagree			Fully agree
Everything worked as it should.	☐	☐	☐	☐
It was easy to use the online website for the games.	☐	☐	☐	☐
I liked the online games.	☐	☐	☐	☐
It was easy to get in touch with the coach through Microsoft Teams/Zoom.	☐	☐	☐	☐
Meeting with a coach in person would be better.	☐	☐	☐	☐
I felt connected to the coach.	☐	☐	☐	☐
I felt that the coach contributed to my learning beyond the sessions.	☐	☐	☐	☐
The information from the coaching sessions was helpful to me.	☐	☐	☐	☐
I enjoyed the coaching sessions.	☐	☐	☐	☐
I enjoyed the analogue games.	☐	☐	☐	☐
It was easy to understand the game instructions (videos and or brain cards).	☐	☐	☐	☐
The program (as a whole) was too long.	☐	☐	☐	☐
The program (as a whole) was too short.	☐	☐	☐	☐
The program (as a whole) was as I expected it to be.	☐	☐	☐	☐
I liked the program (as a whole).	☐	☐	☐	☐
The content was not relevant to me.	☐	☐	☐	☐
The program (as a whole) was useful to me.	☐	☐	☐	☐
I would do the program (as a whole) again.	☐	☐	☐	☐
I would recommend the program (as a whole) to other children.	☐	☐	☐	☐
What part of the program did you enjoy the most?	☐	☐	☐	☐
What part of the program helped you the most?	☐	☐	☐	☐

	no	yes		
Could any part be dropped?	☐	☐	If yes which one?	

TEEN HEART STUDY E-FIT

	Fully disagree			Fully agree
I applied the skills I learned during the program.	☐	☐	☐	☐
I have a plan for dealing with future problems.	☐	☐	☐	☐
I made positive changes.	☐	☐	☐	☐
I have used skills to get more organized.	☐	☐	☐	☐
I have used remembering strategies to remember things better.	☐	☐	☐	☐
I have used techniques to plan.	☐	☐	☐	☐
I have achieved the goals I had at the beginning of the program.	☐	☐	☐	☐
I have a more positive attitude than before.	☐	☐	☐	☐
I am better at solving problems.	☐	☐	☐	☐
I am better at remembering things.	☐	☐	☐	☐
I am more organized.	☐	☐	☐	☐
I know strategies that help me pay attention and remember.	☐	☐	☐	☐
I start things, like homework or chores, better than I used to.	☐	☐	☐	☐
I can control my emotions better than I used to.	☐	☐	☐	☐
I can control my actions better than I used to.	☐	☐	☐	☐
I am better able to stop and think before I act.	☐	☐	☐	☐
I can better cope with unforeseen changes in plans.	☐	☐	☐	☐
I feel less stressed.	☐	☐	☐	☐
I do better in school.	☐	☐	☐	☐

For peer review only - http://bmjopen.bmj.com/site/about/guidelines.xhtml

Everyday Activities Diary

List of activities: School *1 box = 1 quarter of an hour*

What did your child do today?

Please indicate your child's activities today (to the nearest quarter of an hour)

		06:00	07:00	08:00	09:00	10:00	11:00	12:00
**General**								
Sleeping								
Eating								
**School**								
Lessons								
Physical education								
Recess								
**Leisure time**								
Homework								
Reading or browsing through books on his/her own								
Playing a musical instrument or singing								
Watching TV, videos or DVDs								
Computer or video games, Playstation, Nintendo or the Internet								
Other quite activities or games (listening to music or stories, drawing, painting, handiwork or board games)								
Relatively vigorous games with friends or siblings (playground, role-playing, hide-and-seek, etc.)	Indoor							
	Outdoor							
Vigorous games, running around or ball games (not in a sports club)	Indoor							
	Outdoor							
Training, riding or ballet (in a club)	Indoor							
	Outdoor							
**Travel**								
By foot								
By bicycle, scooter, inline skates or skateboard								
By train, tram or bus								
By car								
**Special**								
Activity difficult to classify								

only

		12:00	13:00	14:00	15:00	16:00	17:00	18:00	19:00	20:00	21:00	22:00	23:00	24:00	01:00	02:00	03:00	04:00	05:00	06:00
**General**																				
Sleeping																				
Eating																				
**School**																				
Lessons																				
Physical education																				
Recess																				
**Leisure time**																				
Homework																				
Reading or browsing through books on his/her own																				
Playing a musical instrument or singing																				
Watching TV, videos or DVDs																				
Computer or video games, Playstation, Nintendo or the Internet																				
Other quite activities or games (listening to music or stories, drawing, painting, handiwork or board games)																				
Relatively vigorous games with friends or siblings (playground, role-playing, hide-and-seek, etc.)	Indoor																			
	Outdoor																			
Vigorous games, running around or ball games (not in a sports club)	Indoor																			
	Outdoor																			
Training, riding or ballet (in a club)	Indoor																			
	Outdoor																			
**Travel**																				
By foot																				
By bicycle, scooter, inline skates or skateboard																				
By train, tram or bus																				
By car																				
**Special**																				
Activity difficult to classify																				

UNIVERSITÄTS-
KINDERSPITAL
ZÜRICH
Forschungszentrum
für das Kind FZK

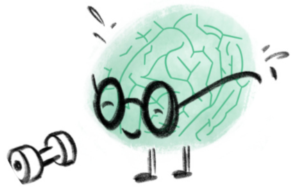

Coaching Content Preparatory Session

Timepoint	Content		Item number
	Explained why we did the assessment	☐ yes ☐ no	1
	Neuroinfo for Kids (NIK) page 3 read aloud	☐ yes ☐ no	2
	Strengths and weaknesses sheet filled in	☐ yes ☐ no	3
	NIK page 5 read aloud	☐ yes ☐ no	4
	NIK page 6 read aloud	☐ yes ☐ no	5
	Results discussed on normal distribution	☐ yes ☐ no	6
	NIK results (2) page read aloud	☐ yes ☐ no	7
	NIK page 10 read aloud	☐ yes ☐ no	8
	Letter written	☐ yes ☐ no	9
	Duration of session 60min	☐ yes ☐ no<br>If no, duration in min:<br>___________	10

For peer review only

Coaching Content EF Sessions 1-6

Timepoint	Content		Item number
	Question asked:<br>Were you able to try out what we talked about last week?	☐ yes ☐ no	10
	Question asked:<br>Did you play the card games? (Which ones?)	☐ yes ☐ no	11
	Question asked:<br>Did everything work out with the online games? I saw you played 3x last week and always started 2x, great! (if necessary point out if something was forgotten)	☐ yes ☐ no	12
	Question asked:<br>Did you add the stickers to your poster?	☐ yes ☐ no	13
	Session topic explained	☐ yes ☐ no	14
	Session goal explained	☐ yes ☐ no	15
	All listed pages of the story read aloud/viewed	☐ yes ☐ no	16
	Assignment completed as described in the session guideline	☐ yes ☐ no	17
	Homework assigned	☐ yes ☐ no	18
	Session duration 60min	☐ yes ☐ no<br>If no, duration in min:<br>__________	19

E-Fit Fidelity Measurement System

For peer review only

Coaching Content Last Session

Timepoint	Content		Item number
	Question asked: Were you able to try out what we talked about last week?	☐ yes ☐ no	10.7
	Question asked: Did you play the card games? (Which ones?)	☐ yes ☐ no	11.7
	Question asked: Did everything work out with the online games? I saw you played 3x last week and always started 2x, great! (if necessary point out if something was forgotten)	☐ yes ☐ no	12.7
	Question asked: Did you add the stickers to your poster?	☐ yes ☐ no	13.7
	Overview of this session provided	☐ yes ☐ no	20
	Summary of the last 8 weeks	☐ yes ☐ no	21
	Evaluation questions asked according to guideline	☐ yes ☐ no	22
	How to continue explained according to guideline	☐ yes ☐ no	23
	Questions and comments of the family clarified	☐ yes ☐ no	24
	Session duration 60min	☐ yes ☐ no If no, duration in min: ____________	19.7

For peer review only - http://bmjopen.bmj.com/site/about/guidelines.xhtml

Computerized Games

Date	Content		Item number
	Sessions 1&2 completed	☐ yes ☐ no	25
	Sessions 3&4 completed	☐ yes ☐ no	26
	Sessions 5&6 completed	☐ yes ☐ no	27
	Sessions 7&8 completed	☐ yes ☐ no	28
	Sessions 9&10 completed	☐ yes ☐ no	29
	Sessions 11&12 completed	☐ yes ☐ no	30
	Sessions 13&14 completed	☐ yes ☐ no	31
	Sessions 15&16 completed	☐ yes ☐ no	32
	Sessions 17&18 completed	☐ yes ☐ no	33
	Sessions 19&20 completed	☐ yes ☐ no	34
	Sessions 21&22 completed	☐ yes ☐ no	35
	Sessions 23&24 completed	☐ yes ☐ no	36
	Sessions 25&26 completed	☐ yes ☐ no	37

For peer review only

For peer review only - http://bmjopen.bmj.com/site/about/guidelines.xhtml

Analogue Games

Duration	Content		Item number
	Gold	☐ yes ☐ no	38
	Dodelido	☐ yes ☐ no	39
	Särge schubsen	☐ yes ☐ no	40
	Geistesblitz 2.0	☐ yes ☐ no	41
	Beaver Gang	☐ yes ☐ no	42
	Beaver Clan	☐ yes ☐ no	43
	Memo Dice	☐ yes ☐ no	44
	5-er finden	☐ yes ☐ no	45
	The nasty 7	☐ yes ☐ no	46
	Yokai	☐ yes ☐ no	47
	Okiya	☐ yes ☐ no	48
	Perplexus Crazy run 2 PE	☐ yes ☐ no	49
	Perplexus Crazy run 5 FT 208	☐ yes ☐ no	50
	Perplexus Crazy run 6 PE Epic	☐ yes ☐ no	51
	Thunder Pops	☐ yes ☐ no	52

For peer review only - http://bmjopen.bmj.com/site/about/guidelines.xhtml

Table S1 Examples of item construction

Categories	Examples	Summary	Item
1 Identity/EF	*"But what I would certainly like for [my son] is … that he is trained a bit. … What should [he] do first, or how can [he] plan the day."*	Daily planning as an everyday problem.	Children with congenital heart defects structure their everyday life
2 Identity/Relation to peers	*"he needs more time and he's a bit slower and then he sees [his] siblings [and] that they're always faster, and then he always makes himself small and that's a shame."*	Is slower than Peers which affects him negatively.	Children with congenital heart defects feel equal compared to their siblings/peers
3 Scope of intervention/game suggestions	*"analog games would also be OK, [I]can imagine that."*	Analog games.	Dice games should be part of such an intervention.
4 Scope of intervention/structure	*"we would like it if it were possible online, because travelling … is always- … a bit far, yes. Especially during school time"*	Online is better for families who live further away from the hospital.	The intervention should be conducted online
5 Scope of intervention/possible distracting factors	*"they just have … a lot of homework …, then he plays the cello, then he goes to the youth club, everything interests him."*	Other hobbies also need a lot of time and should still have room.	Hobbies could get in the way of the intervention
6 Scope of intervention/possible supporting factors	*"That it [should not be like] 'oh that in addition,' but 'great, … that would be great right now and is manageable in terms of time'."*	Time-manageable and fun.	This is the maximum amount of time per day the participants should work on the intervention (in free time):
7 Dealing with the disease/challenges	*"[that] is … something that … she finds quite difficult and where she probably swallows a lot."*	She finds it difficult to talk about her illness and swallows a lot.	Children with congenital heart defects talk about their heart defect
8 Dealing with the disease/supporting factors	*"contact with other heart children, is certainly motivating."*	Contact with other heart children can be motivating.	During the intervention, the children and adolescents should have contact with other children and adolescents
9 Further ideas	*"I think [contact with other children] will motivate them more than if they do their exercise in their corner [or] maybe talk to someone from your team from time to time"*	Contact with other children with CHD or with the study team	In such an intervention, there should be face-to-face meetings with the study team

For peer review only

Table S2 Timeline of the study protocol

	Study timeline				
	**Enrolment**	**Allocation**	**Post-Allocation**		
**TIMEPOINT**			***Baseline***	***+ 2 months***	***+ 6 months***
**ENROLMENT:**					
**Informed consent**	x				
**Eligibility screen**	x				
**Allocation**		x			
**INTERVENTIONS:**					
E-Fit Intervention			◆――	――◆	
Daily Diary			◆――	――◆	
**ASSESSMENTS:**					
IQ			x		
EF assessment			x	x	x
Questionnaires			x	x	x
Feasibility	x	x	◆――	――◆	
SES			x		
Medical information			x		

Note : EF = Executive function; SES = socioeconomic status

For peer review only

Table S3 Example week for a participating family

	Exemplar week					
	MO	***TUE***	***WED***	***THU***	***FRI***	***WEEKEND***
**E-FIT INTERVENTION**						
Parents	30min Call					
Child	60min Coaching Session[a]	20min CogniFit[b]		20min CogniFit[b]	20min CogniFit[b]	Card games
**CONTROL GROUP**						
Parents	10min Diary		10min Diary		10min Diary	10min Diary
Child	10min Diary		10min Diary		10min Diary	10min Diary

Note: a = Filling in Session Rating Scale; b = Filling in Children's Feeling scale and Fun and Demand

For peer review only

Table S4. Computerized training (CogniFit Inc © 2022)

Game	Trained skills			
	flexibility	working memory	planning	inhibition
Traffic Manager			x	
Water Lilies		x	x	
Reaction Field	x			x
Happy Hopper				x
Neuron Madness				x
Minus Malus	x	x		
Tennis Bowling	x			
Candy Line Up		x	x	
Bee Balloon	x			
Slice and Drop			x	
Cube Foundry			x	

Note: x *= primarily trained functions,* x *= additionally trained functions.*

For peer review only

Table S5. Analog training

Game	Trained skills				Players
	flexibility	working memory	planning	inhibition	
Gold (Game Factory ®)	x	x	x		2-5
Dodelido ("Drei Magier ®")	x	x		x	2-6
Särge schubsen ("Drei Magier ®")	x	x	x	x	2-6
Geistesblitz 2.0 ("Zoch")	x	x		x	2-8
Beaver Gang ("Amigo Spiele")	x	x	x		2-6
Beaver Clan ("Amigo Spiele")	x	x	x		2-6
Memo Dice ("Amigo Spiele")	x	x	x		2-4
5er finden (HABA ®)	x		x		1-4
The nasty 7 ("Drei Hasen in der Abendsonne")	x	x		x	2-6
Yokai (Game Factory ®)	x	x	x		2-4
Okiya ("Pegasus Spiele")	x		x		2
Perplexus Crazy run (2 PE Original, 5 FT 208, 6 PE Epic)				x	1
Thunder Pops				x	1

Games selected based on https://www.spielendfoerdern.ch/_files/ugd/8fa6b3_f84a167cfa3c405f8d3244a83242e7ac.pdf

For peer review only

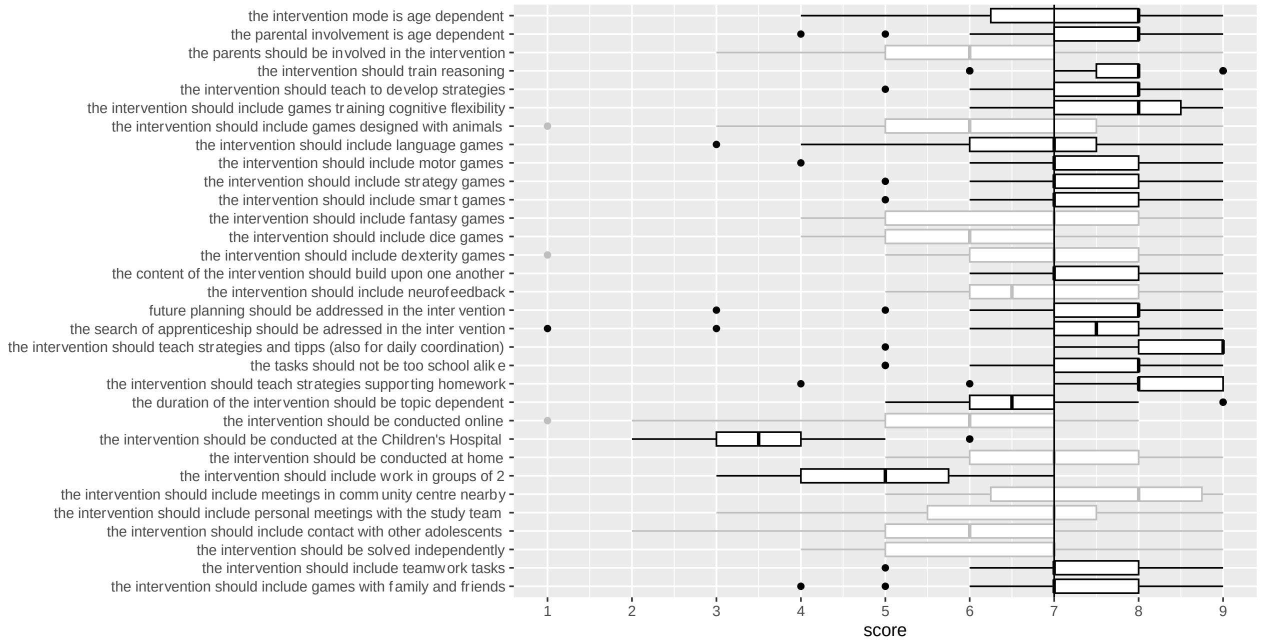

Figure S1. Ratings on Delphi items. 1 = fully disagree, 9 = fully agree. With a median of 7 and an IQR < 2 the item was considered accepted by the participants. Items in gray are the items for which no consensus was found.

For peer review only - http://bmjopen.bmj.com/site/about/guidelines.xhtml

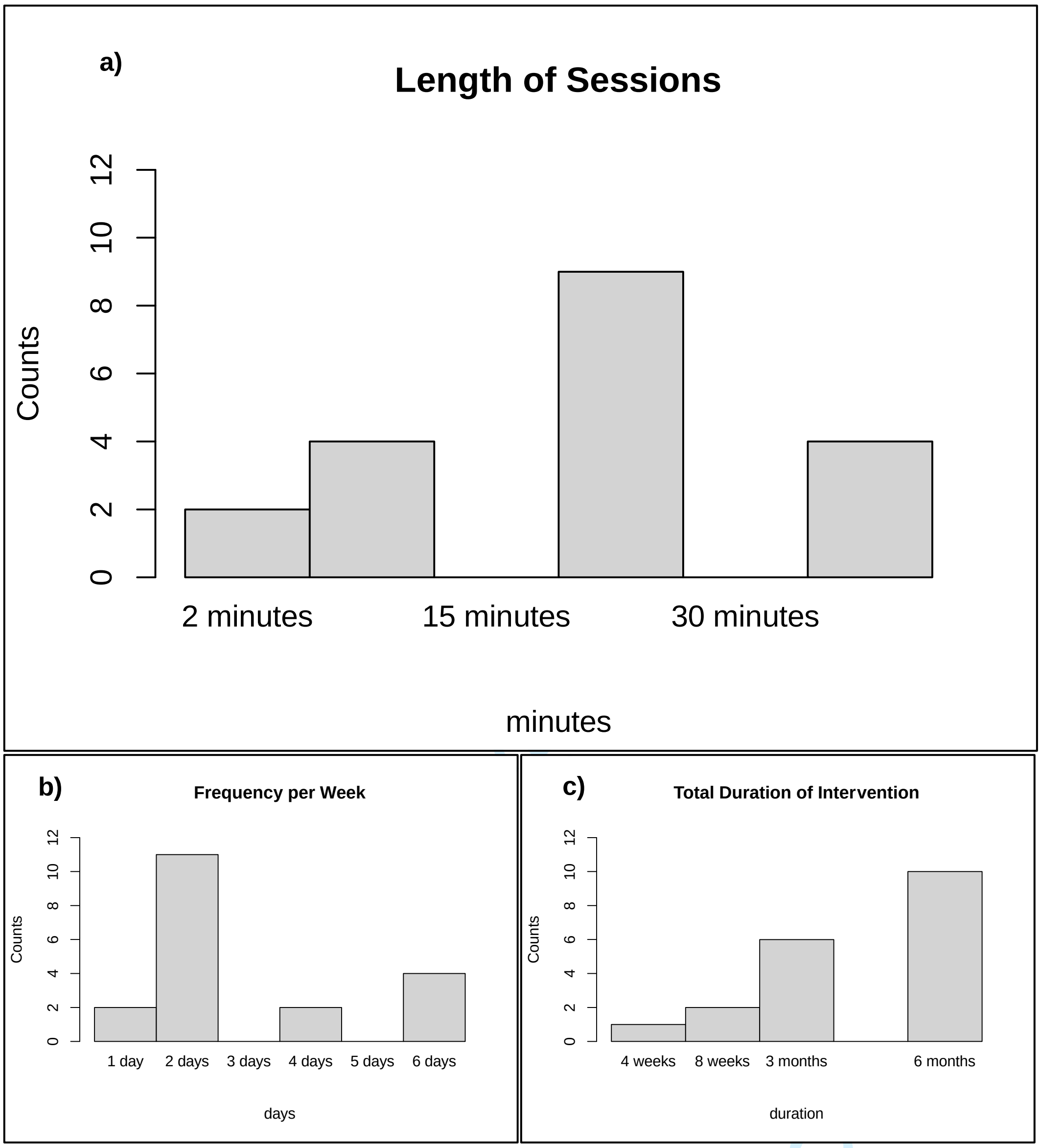

Figure S2. Counts of votes for a) length of sessions, b) frequency per week, and c) duration of the intervention.

For peer review only - http://bmjopen.bmj.com/site/about/guidelines.xhtml

SPIRIT

STANDARD PROTOCOL ITEMS: RECOMMENDATIONS FOR INTERVENTIONAL TRIALS

SPIRIT 2013 Checklist: Recommended items to address in a clinical trial protocol and related documents*

Section/item	Item No	Description	Page No
**Administrative information**			
Title	1	Descriptive title identifying the study design, population, interventions, and, if applicable, trial acronym	1
Trial registration	2a	Trial identifier and registry name. If not yet registered, name of intended registry	2
	2b	All items from the World Health Organization Trial Registration Data Set	See https://register.clinicaltrials.gov/teenheart
Protocol version	3	Date and version identifier	1
Funding	4	Sources and types of financial, material, and other support	19
Roles and responsibilities	5a	Names, affiliations, and roles of protocol contributors	19, 6_ Appendix p.2
	5b	Name and contact information for the trial sponsor	1
	5c	Role of study sponsor and funders, if any, in study design; collection, management, analysis, and interpretation of data; writing of the report; and the decision to submit the report for publication, including whether they will have ultimate authority over any of these activities	16
	5d	Composition, roles, and responsibilities of the coordinating centre, steering committee, endpoint adjudication committee, data management team, and other individuals or groups overseeing the trial, if applicable (see Item 21a for data monitoring committee)	2_Appendix p.2
**Introduction**			
Background and rationale	6a	Description of research question and justification for undertaking the trial, including summary of relevant studies (published and unpublished) examining benefits and harms for each intervention	p. 4-5
	6b	Explanation for choice of comparators	n.a.
Objectives	7	Specific objectives or hypotheses	p. 6

For peer review only - http://bmjopen.bmj.com/site/about/guidelines.xhtml

Trial design	8	Description of trial design including type of trial (eg, parallel group, crossover, factorial, single group), allocation ratio, and framework (eg, superiority, equivalence, noninferiority, exploratory)	p. 9
**Methods: Participants, interventions, and outcomes**			
Study setting	9	Description of study settings (eg, community clinic, academic hospital) and list of countries where data will be collected. Reference to where list of study sites can be obtained	p. 9
Eligibility criteria	10	Inclusion and exclusion criteria for participants. If applicable, eligibility criteria for study centres and individuals who will perform the interventions (eg, surgeons, psychotherapists)	p. 9
Interventions	11a	Interventions for each group with sufficient detail to allow replication, including how and when they will be administered	p.10-12 4_Appendix
	11b	Criteria for discontinuing or modifying allocated interventions for a given trial participant (eg, drug dose change in response to harms, participant request, or improving/worsening disease)	n.a.
	11c	Strategies to improve adherence to intervention protocols, and any procedures for monitoring adherence (eg, drug tablet return, laboratory tests)	4_Appendix
	11d	Relevant concomitant care and interventions that are permitted or prohibited during the trial	n.a.
Outcomes	12	Primary, secondary, and other outcomes, including the specific measurement variable (eg, systolic blood pressure), analysis metric (eg, change from baseline, final value, time to event), method of aggregation (eg, median, proportion), and time point for each outcome. Explanation of the clinical relevance of chosen efficacy and harm outcomes is strongly recommended	p.12-13
Participant timeline	13	Time schedule of enrolment, interventions (including any run-ins and washouts), assessments, and visits for participants. A schematic diagram is highly recommended (see Figure)	p.19
Sample size	14	Estimated number of participants needed to achieve study objectives and how it was determined, including clinical and statistical assumptions supporting any sample size calculations	p.13-14

For peer review only - http://bmjopen.bmj.com/site/about/guidelines.xhtml

Recruitment	15	Strategies for achieving adequate participant enrolment to reach target sample size	p.9
**Methods: Assignment of interventions (for controlled trials)**			
Allocation:			
Sequence generation	16a	Method of generating the allocation sequence (eg, computer-generated random numbers), and list of any factors for stratification. To reduce predictability of a random sequence, details of any planned restriction (eg, blocking) should be provided in a separate document that is unavailable to those who enrol participants or assign interventions	p.10
Allocation concealment mechanism	16b	Mechanism of implementing the allocation sequence (eg, central telephone; sequentially numbered, opaque, sealed envelopes), describing any steps to conceal the sequence until interventions are assigned	p.10
Implementatio n	16c	Who will generate the allocation sequence, who will enrol participants, and who will assign participants to interventions	p.10
Blinding (masking)	17a	Who will be blinded after assignment to interventions (eg, trial participants, care providers, outcome assessors, data analysts), and how	p.10
	17b	If blinded, circumstances under which unblinding is permissible, and procedure for revealing a participant's allocated intervention during the trial	n.a.
**Methods: Data collection, management, and analysis**			
Data collection methods	18a	Plans for assessment and collection of outcome, baseline, and other trial data, including any related processes to promote data quality (eg, duplicate measurements, training of assessors) and a description of study instruments (eg, questionnaires, laboratory tests) along with their reliability and validity, if known. Reference to where data collection forms can be found, if not in the protocol	6_Appendix p.2
	18b	Plans to promote participant retention and complete follow-up, including list of any outcome data to be collected for participants who discontinue or deviate from intervention protocols	6_Appendix p.2
Data management	19	Plans for data entry, coding, security, and storage, including any related processes to promote data quality (eg, double data entry; range checks for data values). Reference to	6_Appendix p.2

For peer review only - http://bmjopen.bmj.com/site/about/guidelines.xhtml

		where details of data management procedures can be found, if not in the protocol	
Statistical methods	20a	Statistical methods for analysing primary and secondary outcomes. Reference to where other details of the statistical analysis plan can be found, if not in the protocol	p.14
	20b	Methods for any additional analyses (eg, subgroup and adjusted analyses)	n.a.
	20c	Definition of analysis population relating to protocol non-adherence (eg, as randomised analysis), and any statistical methods to handle missing data (eg, multiple imputation)	p.14
**Methods: Monitoring**			
Data monitoring	21a	Composition of data monitoring committee (DMC); summary of its role and reporting structure; statement of whether it is independent from the sponsor and competing interests; and reference to where further details about its charter can be found, if not in the protocol. Alternatively, an explanation of why a DMC is not needed	p. 19, 6_Appendix p.2,
	21b	Description of any interim analyses and stopping guidelines, including who will have access to these interim results and make the final decision to terminate the trial	n.a.
Harms	22	Plans for collecting, assessing, reporting, and managing solicited and spontaneously reported adverse events and other unintended effects of trial interventions or trial conduct	6_Appendix p.2
Auditing	23	Frequency and procedures for auditing trial conduct, if any, and whether the process will be independent from investigators and the sponsor	n.a.
**Ethics and dissemination**			
Research ethics approval	24	Plans for seeking research ethics committee/institutional review board (REC/IRB) approval	p.15
Protocol amendments	25	Plans for communicating important protocol modifications (eg, changes to eligibility criteria, outcomes, analyses) to relevant parties (eg, investigators, REC/IRBs, trial participants, trial registries, journals, regulators)	6_Appendix p.3
Consent or assent	26a	Who will obtain informed consent or assent from potential trial participants or authorised surrogates, and how (see Item 32)	p.9
	26b	Additional consent provisions for collection and use of participant data and biological specimens in ancillary studies, if applicable	n.a.

For peer review only

Confidentiality	27	How personal information about potential and enrolled participants will be collected, shared, and maintained in order to protect confidentiality before, during, and after the trial	6_Appendix p.2
Declaration of interests	28	Financial and other competing interests for principal investigators for the overall trial and each study site	p. 1
Access to data	29	Statement of who will have access to the final trial dataset, and disclosure of contractual agreements that limit such access for investigators	6_Appendix p.2
Ancillary and post-trial care	30	Provisions, if any, for ancillary and post-trial care, and for compensation to those who suffer harm from trial participation	n.a.
Dissemination policy	31a	Plans for investigators and sponsor to communicate trial results to participants, healthcare professionals, the public, and other relevant groups (eg, via publication, reporting in results databases, or other data sharing arrangements), including any publication restrictions	p.15
	31b	Authorship eligibility guidelines and any intended use of professional writers	6_Appendix p.2
	31c	Plans, if any, for granting public access to the full protocol, participant-level dataset, and statistical code	n.a.
**Appendices**			
Informed consent materials	32	Model consent form and other related documentation given to participants and authorised surrogates	3_Appendix
Biological specimens	33	Plans for collection, laboratory evaluation, and storage of biological specimens for genetic or molecular analysis in the current trial and for future use in ancillary studies, if applicable	n.a.

*It is strongly recommended that this checklist be read in conjunction with the SPIRIT 2013 Explanation & Elaboration for important clarification on the items. Amendments to the protocol should be tracked and dated. The SPIRIT checklist is copyrighted by the SPIRIT Group under the Creative Commons "Attribution-NonCommercial-NoDerivs 3.0 Unported" license.

For peer review only - http://bmjopen.bmj.com/site/about/guidelines.xhtml

BMJ Open

Multimodal personalized executive function intervention (E-Fit) for school-age children with complex congenital heart disease: protocol for a randomized controlled feasibility study

Journal:	*BMJ Open*
Manuscript ID	bmjopen-2023-073345.R2
Article Type:	Protocol
Date Submitted by the Author:	09-Sep-2023
Complete List of Authors:	Schmid, Alenka; University Children's Hospital Zurich, Child Development Center<br>Ehrler, Melanie; University Children Hospital Zurich, Child Development Center; University Children's Hospital Zurich, Children's Research Center<br>Wehrle, Flavia; University Children’s Hospital Zurich, Child Development Center; University Children’s Hospital Zurich, Children’s Research Center<br>Tuura, Ruth; University Children's Hospital of Zurich, Child Development Center; University Children's Hospital Zurich, Children's Research Center<br>Kretschmar, Oliver; Universitäts-Kinderspital Zürich, Department of Pediatric Cardiology; University of Zurich Faculty of Medicine,<br>Landolt, Markus; University Children's Hospital Zurich, Department of Psychosomatics and Psychiatry; University of Zurich, Division of Child and Adolescent Health Psychology<br>Latal, Beatrice; University Children’s Hospital Zurich, Child Development Center; University of Zurich
**Primary Subject Heading**:	Paediatrics
Secondary Subject Heading:	Patient-centred medicine, Qualitative research
Keywords:	Developmental neurology & neurodisability < PAEDIATRICS, Congenital heart disease < CARDIOLOGY, Adolescent, Feasibility Studies

SCHOLARONE™ Manuscripts

Multimodal personalized executive function intervention (E-Fit) for school-age children with complex congenital heart disease: protocol for a randomized controlled feasibility study

Alenka Schmid[1], Melanie Ehrler[1,2,3], Flavia M. Wehrle[1,2,5], Ruth O'Gorman Tuura[1,2,4,6], Oliver Kretschmar[2,4,7], Markus A. Landolt[2,8,9], Beatrice Latal[1,2,3,4]

[1]Child Development Center, University Children's Hospital Zurich, Zurich, Switzerland

[2]Children's Research Center, University Children's Hospital Zurich, Zurich, Switzerland

[3]URPP Adaptive Brain Circuits in Development and Learning, University of Zurich, Zurich, Switzerland

[4]University of Zurich, Zurich, Switzerland

[5]Department of Neonatology and Intensive Care, University Children's Hospital Zurich, Zurich, Switzerland

[6]MR Research Centre, University Children's Hospital Zurich, Zurich, Switzerland

[7]Department of Cardiology, University Children's Hospital Zurich, Zurich, Switzerland

[8]Department of Psychosomatics and Psychiatry, University Children's Hospital Zurich, Zurich, Switzerland

[9]Division of Child and Adolescent Health Psychology, Department of Psychology, University of Zurich, Zurich, Switzerland

ORCID iDs

Alenka Schmid https://orcid.org/0000-0001-5013-0530

Melanie Ehrler https://orcid.org/0000-0003-4253-2016

Flavia M. Wehrle https://orcid.org/0000-0001-5992-0424

Markus A. Landolt https://orcid.org/0000-0003-0760-5558

Correspondence to

Prof. Dr. Beatrice Latal; bea.latal@kispi.uzh.ch

Child Development Center

University Children's Hospital Zurich

Steinwiesstrasse 75, 8032 Zurich, Switzerland

Protocol version

17.08.23, Version 2.0

Keywords: Feasibility Studies; Developmental neurology & neurodisability; Congenital heart disease; Adolescent

For peer review only

Abstract

Introduction

Children with congenital heart disease (CHD) are at risk for executive functions (EF) impairments. To date, interventions have limited effects on EF in children and adolescents with complex CHD. Therefore, we developed a new multimodal and personalized EF intervention. This study aims to test the feasibility of this intervention called “E-Fit” for children with complex CHD and EF impairments.

Methods and analysis

This is a single-center, single-blinded, randomized controlled feasibility study exploring the E-Fit intervention. We aim to enroll 40 children with CHD aged 10 to 12 years who underwent infant cardiopulmonary bypass surgery and show clinically relevant EF impairments (T-score ≥ 60 on any Behavior Rating Inventory for Executive Function questionnaire summary scale). The multimodal intervention was developed with focus groups and the Delphi method involving children and adolescents with CHD, their parents and teachers, and health professionals. The intervention is composed of three elements: computer-based EF training using CogniFit Inc © 2022, performed three times a week at home; weekly EF remote strategy coaching; and analog games. The content of the computer and strategy training is personalized to the child’s EF difficulties. The control group follows their daily routines as before and completes a diary about their everyday activities four times a week. Participants will be randomized in a 1:1 ratio. Feasibility is measured by the participants’ and providers’ ratings of the participants’ adherence and exposure to the intervention, recruitment rates, and the evaluation of the intended effects of the program.

Ethics and dissemination

Local ethics committee approval was obtained for the study (BASEC-Nr: 2021-02413). Parents provide written informed consent. Key outputs from the trial will be disseminated through presentations at conferences, peer-reviewed publications, and directly to participating families. Furthermore, these results will inform the decision whether to proceed to a randomized controlled trial to investigate effectiveness.

Trial registration

ClinicalTrials.gov, NCT05198583.

For peer review only

Article Summary

Strengths and limitations of this study

- The intervention was designed based on extensive input from patients with CHD, families, teachers, and health professionals provided through focus group interviews and the Delphi method.
- Using a multiple-domain approach, the intervention will be personalized toward individual patients' and families' needs.
- Assessors are blinded to the group allocation of participants.
- Families of the control group may be aware of the content of the intervention and engage in its activities, which might reduce the effect sizes of the intervention.
- Considering the limited sample size of this feasibility study, the effectiveness of the training will only be estimated exploratorily.

For peer review only

Introduction

Eight out of 1000 children are born with congenital heart disease (CHD; [1]); of these, one third require open heart surgery during infancy [2]. In these children, neurodevelopmental problems are known to be a main comorbidity [3]. Studies have shown that the mean IQ of children with CHD is within the normal range but lower than that of their healthy peers [4]. Despite this normal intellectual functioning, children with complex CHD, particularly, are at risk for higher-order cognitive deficits: executive function (EF) impairments [4,5]. The main EF are inhibition; cognitive flexibility, including fluency; planning; and working memory [4–10]. Children with CHD show mild to moderate impairments in all these EF domains [4,5] without a clear predominance of any specific EF domain. In typically developing children, EF are more closely related to school readiness than IQ, and EF predict academic achievement [11]. Accordingly, EF difficulties in childhood have been related to higher unemployment rates in adulthood [12]. Further, EF difficulties in adulthood can increase the risk of addictive behavior and psychiatric diseases such as anxiety and depression [13,14].

Many sequelae of EF difficulties have also been demonstrated in children with CHD. They face lower school achievement and lower psychosocial quality of life (QoL) during school age [15,16]. Further, lower EF have been associated with a higher rate of internalizing symptoms in adolescents and adults with CHD [17]. Given the lifelong academic, psychological, and social burden associated with EF difficulties, evidence-based interventions improving EF functions are urgently needed, particularly for children and adolescents with CHD [18,19].

EF intervention

To date, only two studies have investigated the efficacy of EF interventions in children with CHD. Both studies included school-aged children and adolescents with CHD and tested Cogmed Working Memory Training (Cogmed; [20,21]). Cogmed is a computerized program designed to improve attention and working memory, a core dimension of EF [22]. Participants completed regular Cogmed sessions at home for at least five weeks [21,23]. These studies demonstrated inconsistent findings: Calderon et al. found no improvement of working memory either at postintervention or at 3-month follow-up, but participants who did the Cogmed sessions showed significant improvements in inhibitory control and self-regulation at the 3-month follow-up. Parents reported improved cognitive regulatory skills at post-intervention and 3-month follow-up [20].

In contrast, Jordan et al. found improved working memory postintervention in participants who were assigned to Cogmed compared to the control group. However, this improvement did not persist longer than 6-months follow up [21]. These findings are in line with the inconsistent results of a recent meta-analysis of EF

For peer review only

interventions in other children at risk for EF impairments [24]. This meta-analysis reported only small to moderate effects of computerized interventions of single functions and no evidence for long-term effects. These inconsistencies between EF interventions have led to investigations about factors impacting the effectiveness of an EF intervention [25]. One of those factors is motivation, which influences the effort a participant is willing to make, which further impacts the intervention's success [26]. Game elements are able to increase motivation, and therefore, utilizing computerized interventions with game elements is a favorable option [27]. Also, analog games, such as board and card games, have been tested as EF interventions and have shown effectiveness in enhancing the functions trained [28,29]. Moreover, by requiring children to play with others, such games may enhance self-monitoring [28]. Studies combining computer and analog games demonstrated improvements in trained reasoning and speed tasks in healthy children with low socioeconomic status aged 7 to 10 years [30]. However, despite the enjoyment factor, a major challenge in interventional studies is the limited transfer of training effects to other cognitive abilities [31–34]. Importantly, a meta-analysis revealed that programs emphasizing teaching social-emotional strategies and self-regulation had the most significant positive impact on EF, particularly for children with neurodevelopmental disorders or behavioral problems. These effects appeared to be stronger than those observed in trainings primarily focusing on tasks specifically training EF and may address the transfer of training to general cognitive abilities [18,24,31]. Therefore, exploring intervention approaches beyond computerized and tasks-specific training is crucial [20]. Mindfulness education has improved EF behavior, optimism, and classroom social competence behaviors in healthy children and adolescents [35,36]. Similarly, problem-solving solutions to EF-related challenges in everyday life improved parent- and self-rated global EF behaviors in children with epilepsy [37]. Hence, mindfulness interventions and strategy coaching may address the transfer of training to everyday life by targeting sensory awareness and attention regulation, thus promoting emotional and social development [18,31].

Combining established and engaging computerized and analog training that improves individual EF, alongside strategy coaching aiming at integrating these benefits into daily life, presents a promising approach for addressing the aforementioned limitations of current interventions.

Because children with CHD experience difficulties in various EF domains with significant academic, psychological, and social consequences, an intervention integrating several of these approaches may be particularly suited to improving these children's EF and, eventually, their long-term development and social integration.

For peer review only

Aims and hypotheses

This study aims to test the feasibility of a multimodal personalized EF intervention called E-Fit for children with complex CHD and EF impairments. To evaluate E-Fit's feasibility, the following aims and hypotheses will be explored:

Aim 1: Assess whether children with CHD and EF impairments can adhere to E-Fit.

Hypothesis 1: Study participants will complete at least 80% of the strategy coaching and computerized training sessions of E-Fit.

Aim 2: Assess the acceptability of E-Fit for children with CHD and EF impairments and their parents.

Hypothesis 2: Study participants and their parents will indicate high acceptability of E-Fit as measured by average ratings of 3 or higher on a 4-point rating scale assessing the acceptability of each program component.

Aim 3: Assess the feasibility of E-Fit for children with CHD and EF impairments and their parents.

Hypothesis 3: Study participants and their parents will indicate high feasibility of E-Fit as measured by average ratings of 3 or higher on a 4-point rating scale assessing the user-friendliness and ease of integrating each program component into their daily lives and environments.

Aim 4 (exploratory): Measure the impact of E-Fit on improving EF skills in children with CHD and EF impairments.

Hypothesis 4: We anticipate a moderate effect in the direction of improved EF from pre- to post-intervention in study participants.

The rationale for projection numbers provided in the hypotheses is as follows:

Adherence of 80% is based on previous intervention studies [23].

The decision to use rating scale ratings of 3 or higher is founded on the 4-point rating scale (1 = do not agree at all - 4 fully agree), Therefore, 3 and above are considered affirmative.

Effect sizes are based on the results of a meta-analysis. Specifically, a value of .38 pertains to explicit EF practice (including computerized and analog training across all populations), while .46 corresponds to strategy coaching across all populations [24].

Methods and analysis

In this section, we will first describe the development of the intervention, which involved a needs assessment preceding the present investigation. Subsequently, we will outline the methods utilized in the present feasibility study. For an overview, see figure 1.

For peer review only

Needs assessment

Before developing E-Fit and the present study, we conducted a needs assessment. This assessment aimed to specifically identify the unique EF intervention needs of children with CHD by means of focus group interviews and the Delphi method. Below, you will find comprehensive details about the focus group interviews and the Delphi method, including the outcomes that led to the final intervention.

Focus group interviews

We conducted two separate online focus group interviews: one with five adolescents with CHD aged 14 to 16 years and another with seven of their parents (n_{female}= 5). Participants were recruited from the Teen Heart Study, which investigated EF in children and adolescents with CHD at the University Children's Hospital Zurich. The Teen Heart Study included children and adolescents with CHD without genetic or dysmorphic syndromes who were born between 2004 and 2012 and underwent their first cardiopulmonary bypass (CPB) surgery before the age of 6 years [25]. Participants were asked what they consider the ideal content and scope of an intervention supporting EF in children with CHD (e.g., 1. how much time can or should your child invest per a) week, b) per day, c) how long in total? 2. Which difficulties does your child have in everyday life?; see Appendix 1). These interviews were recorded with the consent of the participants and then transcribed and analyzed with structured qualitative content analysis [26]. This analysis produced nine categories, and all participants' suggestions and statements were assigned to one of these categories (supplemental material, table S1).

Delphi method

The results from the focus group interviews informed the initiation of the Delphi method, aiming to achieve consensus on the intervention's content through three sequential questionnaires [27,28]. During this procedure, the items of a survey are rated and evaluated multiple times, each time anonymously. The participants subsequently receive the next survey, along with feedback on the overall answers [29].

The surveys were completed online by children with CHD (*n* = 7) aged 12 to 13 years who had not participated in the preceding focus group interviews, their teachers (*n* = 5), parents (*n* = 7), and health professionals (n = 5). The health professionals were experts in the field from international hospitals and research institutions who held a comprehensive understanding of the difficulties faced by children with CHD. They had previously engaged with the research group and were contacted by email. The families were again recruited from the Teen Heart Study, but following focus group interview suggestions, only participants between 10 and 13 years old were asked to participate. The families provided the email addresses of the teachers to be contacted by the study team. Hence, the surveys were completed by four stakeholder groups: children with CHD, parents of children with CHD, teachers of children with CHD, and health professionals.

For peer review only

For survey one, respondents received a link by email and were invited to rate each item on a Likert scale from 1 for “fully disagree” to 9 for “fully agree”. They were encouraged to make further suggestions yet to be represented in the survey. Survey two was sent to all respondents of survey one. All items from survey one were included, and additional items suggested by participants were added following revision and categorization. The results of survey one were included in survey two: A table displayed the median and interquartile range (IQR) per stakeholder group for each item, and each participant was reminded of the scores they had given in survey one. Participants were asked to consider the input of all stakeholder groups in survey one and then review their answers. Items reaching consensus (IQR <2) were dropped from the subsequent round. Survey three was sent to all respondents of survey two. Items for which no consensus was reached in survey two were included. No more items were added. Again, the results of survey two were included in survey three. Participants again reviewed their answers. At the end of survey three, every participant was asked about their interest in participating in the final consensus meeting. Throughout the process, all stakeholder groups responded to the same items.

Overall, participants completed three rounds with the goal of consensus-seeking across stakeholder groups, defined as IQR<2 for each item [30]. Items for which no consensus was reached after three rounds were discussed in a final consensus meeting with three parents, two health professionals, and the study team. In the consensus meeting, it became clear that some aspects of the intervention are child-specific and cannot be answered conclusively. To families, it was particularly important to have a diverse intervention that was not too school-like. The researchers commented on the intervention in terms of methodological aspects, which were taken into account in the study design (e.g. additional questionnaires or incentives to be added).

Findings from needs assessment

The results of the Delphi method, including the consensus meeting, showed that the wishes most strongly expressed by parents were that the intervention should include strategies supporting homework and everyday life activities and that the children should learn to develop their own strategies. Families participating in the Delphi method wanted activities that did not resemble schoolwork, improved reasoning skills, and addressed academic future planning (supplemental material, figure S1). Parents emphasized that the ideal age at which an EF intervention should start depends on the individual child, ranging from 4 to 18. Literature shows that EF development progresses considerably during childhood and adolescence [18]. This opens a window of opportunity for EF training during these periods [31,32]. Also, the families’ perceived need for therapy is crucial for adherence [33]. During school age and adolescence, families become more aware of the sequelae of CHD and thus become more willing to participate in an intervention program. Participants also wanted minimal

For peer review only - http://bmjopen.bmj.com/site/about/guidelines.xhtml

parental participation in the intervention, and the focus group interviews indicated that participants became aware of EF problems shortly before high school when remedial action might still be effective.

Consequently, we decided on an age range of 10 to 12 years instead of younger or older. No consensus was reached on whether the intervention should include personal meetings with the study team, whether the intervention should occur in a nearby community center, or on the design of the games (supplemental material, figure S1). The consensus meeting indicated that responses on these topics differed between children and families, and general agreement was difficult to reach. Hence, literature and practice in previous programs were used to decide on these elements [18,20,21,33–39]. For details, see Appendix 2 and supplemental material (figure S2).

Study design to test the feasibility of the intervention

This is a single-center, single-blinded, randomized controlled feasibility study to test the feasibility of the E-Fit intervention.

All participants undergo a baseline neurodevelopmental assessment (table 1) and are randomly then assigned to either the E-Fit intervention or to the control group. All participants undergo a 2-month postintervention and a 6-month follow-up assessment. Investigators administering neuropsychological tests are blinded to the participant's allocation. The families and the psychologist who conducts the intervention are aware of the allocation (supplemental material, table S2).

Table 1. Executive function domains

EF domain	Assessment test
Inhibition	Color Word Interference Task (D-KEFS [40])<br>Go/No-Go (TAP [41])
Cognitive Flexibility	Trail-making Test (D-KEFS [40])<br>Color Word Interference Task (D-KEFS [40])
Planning	Tower Test (D-KEFS [40])
Working Memory	Digit Span (WISC-V [42])<br>Picture Span (WISC-V [42])<br>Number Letter Sequencing (WISC-V [42])
Risk-taking	Balloon Analog Risk Task [43]

Abbreviations: D-KEFS, Delis-Kaplan Executive Function System; EF, executive function; WISC -V, Wechsler Intelligence Scale for Children fifth edition

For peer review only

Participants and recruitment

This study includes children with CHD who underwent CPB in Switzerland before one year of age. Diagnoses may include transposition of the great arteries, hypoplastic left heart syndrome, and other complex CHDs requiring CPB. At recruitment, children are 10-12 years old. Children diagnosed with a genetic or dysmorphic syndrome such as trisomy-21 or Ellis-van Creveld syndrome or with known large cerebral lesions or injuries, including stroke and severe hypoxic-ischemic encephalopathy with significant neurological sequelae preventing them from performing the assessments are excluded.

Recruitment for the study commenced in April 2022 and is planned to continue until fall 2023. Children from the Department of Cardiology outpatient clinic at the University Children's Hospital Zurich are being contacted consecutively. Follow-up assessments are scheduled to be completed by the beginning of 2024. Participants are identified through the clinical database comprising all treated children with CHD. All eligible children with CHD and their parents receive a letter with the study information and are asked to contact the study team if they are interested in participating. The study team provides further information about the study and answers questions on the phone. The contact information of the children's teachers is forwarded by the parents, and after agreeing to participate and giving written informed consent (Appendix 3), parents and the child's principal teacher fill in the Behavior Rating Inventory of Executive Functions (BRIEF [44]) screening questionnaire. The BRIEF assesses EF behaviors in children and adolescents on three summary scales: a Behavior Regulation Index, a Metacognition Index, and a Global Executive Composite. Participants are included in the study if a T-score ≥ 60 on any of the three summary scales is reported by either parents or teachers.

Randomization and stratification

After passing the screening questionnaire, participants will be randomly assigned to either the intervention or control group in a 1:1 design by block randomization (block size = 4, stratification variable: maternal education level on a 6-point Likert scale). This list of random numbers will be generated prior to the start of the study in the statistical software R [45] and implemented in the randomization module on the research electronic database Redcap® [46], which allows allocation to be concealed until the participant is enrolled. At the end of the first assessment, after all tests and questionnaires are completed, the child will open an envelope prepared by a study member otherwise not involved in the study. The note in the envelope will reveal the allocation status to the family and baseline assessor.

For peer review only

Intervention group: E-Fit

The executive function intervention (E-Fit) targets multiple EF domains, focusing on four that have been identified as being impaired by two meta-analyses on EF in children with CHD: inhibition, planning, flexibility, and working memory [4,5]. See Appendix 4 for details. These are addressed in a multimodal approach consisting of three personalized and adaptive elements: 1) computerized training played alone, 2) remote 1:1 strategy coaching with the child and a psychologist, and 3) analog board and card games fostering EF abilities that can be played alone or with friends and family (supplemental material, table S3). The intervention lasts for eight weeks.

Computerized training

Participants use the platform CogniFit Inc © 2022 (CogniFit) for the computerized training. CogniFit was selected based on literature [47,48] and its successful use in other similarly aged populations [49] and because it targets several EF domains. This is in contrast to Cogmed, which only targets working memory. From the CogniFit platform, eleven games targeting the aforementioned EF domains were selected, considering three games per function (supplemental material, table S4). These games involve tasks that require information retention and manipulation, strategy development and task switching [34]. These games are adaptive to the child's level of performance. Participants play the assigned games at home on a computer, smartphone, or tablet. A device is provided if needed. The child is asked to play three times a week for 20 min over the course of the eight-week intervention period.

Strategy coaching

The strategy coaching is conducted remotely. A psychologist is assigned to each family as a coach to monitor and support them throughout the intervention. The child meets with the coach once a week for 60 minutes for a total of eight sessions.

The first session discusses baseline assessment results and the child's strengths and weaknesses [50]. Further, the first module of CogniFit is performed to make sure the child understands how to use CogniFit. In this session, a parent participates with the child.

For the six following sessions, parents fill in a short questionnaire [35] to identify EF domains in which their child faces difficulties. Of eleven EF domains rated, the six indicating the most difficulties are selected for the coaching sessions. Each of these six sessions has standardized guidelines and starts with a story about an EF and an explanation of how this function engages our brain [36–39]. Afterwards, the child completes a task teaching a strategy for the EF in question [51] (for more details, see Appendix 4). During the coaching sessions, solutions to difficulties with adhering to E-Fit are suggested.

For peer review only

The eighth and final coaching session consists of the wrap-up and outlook, where a parent again participates with the child [52].

Additionally, the coach calls the parents in the first and fourth week to discuss the upcoming sessions that will be conducted with the child only. Parents can suggest specific situations to be addressed in the subsequent strategy coaching. In response, the coach provides tips on how parents can support their child with the respective EF [51]. For an overview of the strategy coaching procedure, see table 2.

Following the manual, the strategy coaching teaches strategies to promote EF in everyday life [51]. A clinical neuropsychologist reviewed the strategy coaching, and the manual is available in German language.

Analog training

Thirteen board and card games are provided to the families (supplemental material, table S5). They are suited for this age group and target EF abilities. A special needs and trained game educator helped select the games targeting the above-mentioned EF domains for children between 10 and 12 years of age. Like the computerized training, the analog training also demands a range of diverse functions, including rule retention, flexible adaptation to other players, impulse control, and strategic thinking. Often, a single game may require multiple functions simultaneously. Families are free to play as much as they want but are asked to report the date, duration, and name of the analog games played.

Table 2. Strategy coaching procedure

	Week 1	***Week 2***	***Week 3***	***Week 4***	***Week 5***	***Week 6***	***Week 7***	***Week 8***
Child		60min individual topic	60min individual topic	60min individual topic	60min individual topic	60min individual topic	60min individual topic	
Parent	30min Call			30min Call				
Parent and Child	60min initialization session							45min Wrap up session

Control group

The control group receives standard of care, including cardiac surveillance, neurodevelopmental counseling, and therapies such as speech and physiotherapy. Because the control group families know the scope of the tested intervention, this may lead to contamination: during the trial, parents of control children may engage their children in activities used in the intervention fostering EF functions. To detect this and where occurring, to measure the time spent on it, parents and children complete an electronic diary four times a week for eight

weeks to monitor the child's everyday activities (Appendix 5 and Supplemental material [table S3]). After the final visit at the end of study participation, the control group families receive the list of EF-enhancing analog games used, EF fact sheets, and a 2-month CogniFit login.

Primary outcome measures

The feasibility of the E-Fit intervention will be measured in six areas, as described by Bowen et al. [53]. Except for Practicality and Limited efficacy, primary outcome measures will be collected only in the intervention group throughout the intervention period. For a summary of measures and assessment timepoints, see table 3. The following areas will be assessed.

Acceptability

Acceptability examines suitability, satisfaction, and attractiveness to program deliverers and recipients. It is measured by questions about satisfaction, intent to continue use, and perceived appropriateness throughout the intervention period.

Demand

Demand is assessed by the extent to which the intervention is likely to be used or how much demand exists. This includes measures for the fit within the family, perceived positive or negative effects on the family, actual use, expressed interest or intention to use, and perceived demand throughout the intervention period.

Implementation

To ensure the successful delivery of the intervention to participants, implementation is a critical aspect. The E-Fit Fidelity Measurement System was newly developed following existing guidelines [54]. The purpose of this system is to measure the degree of fidelity with which the newly developed E-Fit was implemented. Essential components, including specific questions to be asked and required session completion, were organized, phrased and complemented by response choices (yes and no). During the study, the Fidelity Measurement System will be piloted and evaluated.

Practicality

Practicality explores how an intervention can be delivered to intended participants with existing means, resources, and circumstances. It includes factors affecting implementation ease or difficulty, efficiency, speed and quality of implementation, positive or negative effects on target participants, and the participant's ability to complete the intervention activities throughout the intervention period.

Integration

Integration assesses the extent to which the intervention can be integrated within the existing system. This addresses the perceived fit with infrastructure and perceived sustainability during the last coaching session.

For peer review only

Limited efficacy

Limited efficacy explores whether the new intervention shows promise of succeeding with the intended population. This is measured by evaluating the program's intended effects or process on key intermediate variables, effect-size estimation, and maintenance of changes from initial change (See table 1 for an overview of EF measures).

For peer review only

Table 3. Feasibility focus areas and assessment tools

Area of focus	Assessment tool	Group	Timepoint	Completed by
Acceptability	Acceptance and Feasibility scale [55]	E-Fit	Final coaching	Parents and children
	Children's Feeling scale [55]	E-Fit	Intervention period, before and after each online training	Children
	Observed engagement [56]	E-Fit	Each coaching session	Coach
Demand	Acceptance and Feasibility scale	E-Fit	Final coaching	Parents and children
	Fun and Demand [57]	E-Fit	Intervention period, before and after each online training	Children
	Session data: duration, number of completed sessions, and for the computerized training, the coaching, and the card games	E-Fit	Intervention period	
	Recruitment rate	E-Fit	End of study	
Implementation	E-Fit Fidelity Measurement System tool, developed based on Feely et. al [54]	E-Fit	End of study	Two separate raters
Practicality	Acceptance and Feasibility scale [55]	E-Fit	Final coaching	Parents and children
	Feedback in coaching sessions	E-Fit	Each coaching session	Parents and children
	Observed involvement [56]	E-Fit	End of study	
	Session rating scale [58]	E-Fit	Each coaching session	Children
	Observed engagement [56]	E-Fit	Each coaching session	Coaches
	Recruitment rates	Both	End of study	
	Retention rates	Both	End of study	
	Completion of weekly sessions	E-Fit	End of study	
	Number of adverse events (AEs)	Both	Continuously	
Integration	Acceptance and Feasibility scale	E-Fit	Final coaching	Parents and children
Limited efficacy	Delis-Kaplan Executive Function System [40]	Both	BL, T_1, T_2	
	Wechsler Intelligence Scale for Children- 5th edition [42]	Both	BL, T_1, T_2	
	Test of Attentional Performance [41]	Both	BL, T_1, T_2	
	Balloon Analog Risk Task [43]	Both	BL, T_1, T_2	
	Behavior Rating Inventory for Executive Function [44]	Both	BL, T_1, T_2	Parents* and teachers

*Note: *if possible, completed by both parents separately*

For peer review only

Secondary outcome measures

In addition to primary outcome measures, we will examine other variables assessing the success of the intervention. The following secondary outcome measures are to be collected from both groups at baseline, 2-month, and 6-month follow-up: The Family Relationship Index is a self-report measure used to evaluate subjective quality of family environment for parents [59]. The Kidscreen-10 assesses the child's subjective health and well-being, and the Kidscreen-27 assesses the child's health and well-being from a parent's perspective; these are combined to assess the child's QoL [60]. The parents will complete the short form of the Conners-3 and the Social Responsiveness Scale [61,62] to screen for behavioral problems. The Resilience Scale (RS-13) is a self-reported questionnaire assessing resilience by acceptance of self-, life- and personal competencies. This questionnaire will be completed by the parents and the child [63].

Covariate measures

Medical, sociodemographic, and patient-specific factors and IQ will be measured in both groups at baseline and be treated as covariates for the effectiveness of the intervention and to describe the population who participated in the intervention.

Socioeconomic status (SES) will be estimated on a six-point scale for both paternal and maternal education, leading to the lowest possible SES sum score of 2 and the highest of 12 [64]. Cardiac and neonatal data will be collected from medical records. A short form of the Wechsler Intelligence Scale for Children, fifth edition, will be used to assess total IQ once at the baseline assessment [42].

Sample size determination

This is a proof-of-concept study to test the feasibility of a new intervention. Accordingly, the primary outcome is feasibility; thus no minimum sample size has been calculated [65,66]. A retrospective power analysis will be conducted. Instead, the sample size was defined from previous literature [66,67]. Using a pilot study with a sample size <30 may lead to the potential to underrecruit for the main study [66]. To provide a high level of confidence, a total sample size between 35 and 55 is suggested to account also for small effect sizes. As the total population to recruit from for the present study is relatively small, we decided on a sample size of 40 (E-Fit intervention group n = 20, control group n = 20).

Approximately 210 children are eligible for screening and are to be contacted. In a study by Spillmann et al. [15] using a cut-off of $\geq$ 65 in the BRIEF, around 20% of children with complex CHD had EF difficulties at 10 years. Using data from children and adolescents between 10 and 15 years and a cut-off of $\geq$ 60, we estimate around 30% of eligible children to demonstrate EF difficulties, leading to a potential sample size of 60 children.

For peer review only

We assume a response rate of around 75% from those families with children with EF problems, leading to a study sample of approximately 45 participants. A drop-out rate of approximately 10% allows the planned sample of 40 participants to be achieved.

Data analysis plan

The feasibility of the intervention will be delineated from the descriptive statistics.

Descriptive statistics will be calculated, including means, SD, medians, and IQRs for continuous variables and frequency counts and percentages for categorical variables. The newly developed E-Fit Fidelity Measurement System will be evaluated. Two raters will complete it individually, and inter-rater reliability will be reported. To report limited efficacy, within-subject differences between EF performances from baseline to post-treatment and 6-month follow-up assessments are to be investigated across treatment groups with an intention-to-treat analysis. Linear regression controlling for covariates, involving differences in means and 95% CI, will be used to assess differences between groups and time points for continuous outcomes. Proportions and logistic regressions for binary outcomes will be used to examine group effects (e.g., comparison of clinically relevant vs. not clinically relevant scores in the BRIEF, $\chi 2$ tests, ORs, and 95% CI). Given that missing values are missing at random, they will be imputed using multiple imputations.

The data will be visually inspected to identify discordant and influential observations. All analyses will be conducted with an alpha level of 0.05. The statistical software R will be used for all analyses [68]. The final project report will describe and justify any deviations from the original statistical plan.

Patient and public involvement

As described above, children and adolescents with CHD, their parents, and their teachers were involved in the intervention development and design. Study results will be disseminated to participants and their families through a newsletter and a public informative meeting.

Ethics and dissemination

The protocol was approved by the ethical committee of the Canton of Zurich in Switzerland (BASEC-Nr: 2021-02413) and was registered on ClinicalTrials.gov (NCT05198583). Written informed consent will be obtained from the parents. Data handling, record keeping, and archiving will be performed according to the guidelines given by the ethical committee's guidelines (for details, see Appendix 6). The results of this study will be disseminated through presentations at national and international conferences, publications in peer-reviewed journals, and directly to families who participated in the study.

For peer review only

Key outputs from the trial will contribute to our dissemination and impact agenda: Pilot data will inform the decision whether to proceed to a definitive randomized controlled trial, and if we do, we will have evidence to support funding applications and a finalized intervention delivery manual to enable multicenter replication of E-Fit.

Discussion

This article outlines the development and design of a randomized controlled feasibility study investigating an 8-week EF intervention called "E-Fit" for children with CHD. Given the limited effects of previous EF interventions, E-Fit was newly developed based on comprehensive input from patients, families, teachers, and health professionals. It includes strategy coaching and both computerized and analog game-based training and is personalized to the individual participant.

This study evaluates feasibility through the areas of focus acceptability, demand, implementation, practicality, integration and limited efficacy. The results of this study will lay the foundation for a main randomized controlled trial to investigate effectiveness in a larger sample. While E-Fit was initially developed to address EF deficits in children with CHD, successful feasibility validation could also broaden the applicability of E-Fit to other pediatric populations facing EF difficulties.

Limitations of the study include that families of the control group may be aware of the content of the intervention and engage in its activities, which might reduce the effect sizes of the intervention. Additionally, considering the limited sample size of this feasibility study, the effectiveness of the training will only be estimated exploratorily.

For peer review only

Contributors

BL, ME, FW, RO'GT, OK, ML, and AS conceived and designed the study. *BL* is the grant holder. *AS* wrote and revised the manuscript. All authors critically revised the manuscript for important intellectual content.

Funding

This work was supported by the Swiss National Science Foundation (SNF) grant number 32003B_172914. This funding source had no role in the design of this study and will not have any role during its execution, analyses, interpretation of data, or decision to submit results. CogniFit Inc © 2022 had no role in the design of this study and will not have any role during its execution, analyses, interpretation of data, or decision to submit results.

Competing interests

The authors have no financial or other competing interests for the overall study.

Acknowledgements

The authors thank Nina Zeltner, who supported the qualitative analysis, and Thomas Pletschko, who reviewed the structure and content of the strategy coaching.

For peer review only

References

1 Bernier P-L, Stefanescu A, Samoukovic G, *et al.* The Challenge of Congenital Heart Disease Worldwide: Epidemiologic and Demographic Facts. *Semin Thorac Cardiovasc Surg Pediatr Card Surg Annu* 2010;**13**:26–34. doi:10.1053/j.pcsu.2010.02.005
2 Wernovsky G, Licht DJ. Neurodevelopmental Outcomes in Children with Congenital Heart Disease – What can we impact? *Pediatr Crit Care Med J Soc Crit Care Med World Fed Pediatr Intensive Crit Care Soc* 2016;**17**:S232–42. doi:10.1097/PCC.0000000000000800
3 Marino BS, Lipkin PH, Newburger JW, *et al.* Neurodevelopmental Outcomes in Children With Congenital Heart Disease: Evaluation and Management. *Circulation* 2012;**126**:1143–72. doi:10.1161/CIR.0b013e318265ee8a
4 Feldmann M, Bataillard C, Ehrler M, *et al.* Cognitive and Executive Function in Congenital Heart Disease: A Meta-analysis. *Pediatrics* 2021;**148**:e2021050875. doi:10.1542/peds.2021-050875
5 Jackson WM, Davis N, Calderon J, *et al.* Executive functions in children with heart disease: a systematic review and meta-analysis. *Cardiol Young* 2021;**31**:1914–22. doi:10.1017/S1047951121001074
6 Diamond A. Executive Functions. *Annu Rev Psychol* 2013;**64**:135–68. doi:10.1146/annurev-psych-113011-143750
7 Lehto JE, Juujärvi P, Kooistra L, *et al.* Dimensions of executive functioning: Evidence from children. *Br J Dev Psychol* 2003;**21**:59–80. doi:10.1348/026151003321164627
8 Miyake A, Friedman NP, Emerson MJ, *et al.* The Unity and Diversity of Executive Functions and Their Contributions to Complex "Frontal Lobe" Tasks: A Latent Variable Analysis. *Cognit Psychol* 2000;**41**:49–100. doi:10.1006/cogp.1999.0734
9 Miyake A, Friedman NP. The Nature and Organization of Individual Differences in Executive Functions: Four General Conclusions. *Curr Dir Psychol Sci* 2012;**21**:8–14. doi:10.1177/0963721411429458
10 Baggetta P, Alexander PA. Conceptualization and Operationalization of Executive Function. *Mind Brain Educ* 2016;**10**:10–33. doi:10.1111/mbe.12100
11 Blair C, Razza RP. Relating Effortful Control, Executive Function, and False Belief Understanding to Emerging Math and Literacy Ability in Kindergarten. *Child Dev* 2007;**78**:647–63. doi:10.1111/j.1467-8624.2007.01019.x
12 Miller M, Nevado-Montenegro AJ, Hinshaw SP. Childhood Executive Function Continues to Predict Outcomes in Young Adult Females with and Without Childhood-Diagnosed ADHD. *J Abnorm Child Psychol* 2012;**40**:657–68. doi:10.1007/s10802-011-9599-y
13 Taylor Tavares JV, Clark L, Cannon DM, *et al.* Distinct Profiles of Neurocognitive Function in Unmedicated Unipolar Depression and Bipolar II Depression. *Biol Psychiatry* 2007;**62**:917–24. doi:10.1016/j.biopsych.2007.05.034
14 Baler RD, Volkow ND. Drug addiction: the neurobiology of disrupted self-control. *Trends Mol Med* 2006;**12**:559–66. doi:10.1016/j.molmed.2006.10.005
15 Spillmann R, Polentarutti S, Ehrler M, *et al.* Congenital heart disease in school-aged children: Cognition, education, and participation in leisure activities. *Pediatr Res* 2021;:1–7. doi:10.1038/s41390-021-01853-4
16 Sanz JH, Wang J, Berl MM, *et al.* Executive Function and Psychosocial Quality of Life in School Age Children with Congenital Heart Disease. *J Pediatr* 2018;**202**:63–9. doi:10.1016/j.jpeds.2018.07.018
17 Jackson JL, Gerardo GM, Monti JD, *et al.* Executive Function and Internalizing Symptoms in Adolescents and Young Adults With Congenital Heart Disease: The Role of Coping. *J Pediatr Psychol* 2018;**43**:906–15. doi:10.1093/jpepsy/jsx154
18 Calderon J, Bellinger DC. Executive function deficits in congenital heart disease: why is intervention important? *Cardiol Young* 2015;**25**:1238–46. doi:10.1017/S1047951115001134
19 Diamond A. Why improving and assessing executive functions early in life is critical. In: *Executive function in preschool-age children: Integrating measurement, neurodevelopment, and translational research*. Washington, DC, US: : American Psychological Association 2016. 11–43. doi:10.1037/14797-002
20 Calderon J, Wypij D, Rofeberg V, *et al.* Randomized Controlled Trial of Working Memory Intervention in Congenital Heart Disease. *J Pediatr* 2020;**227**:191-198.e3. doi:10.1016/j.jpeds.2020.08.038
21 Jordan LC, Siciliano RE, Cole DA, *et al.* Cognitive training in children with hypoplastic left heart syndrome: A pilot randomized trial. *Prog Pediatr Cardiol* 2020;**57**:101185. doi:10.1016/j.ppedcard.2019.101185
22 Cogmed. https://www.cogmed.com/training (accessed 19 Jan 2023).
23 Calderon J, Wypij D, Rofeberg V, *et al.* Randomized Controlled Trial of Working Memory Intervention in Congenital Heart Disease. *J Pediatr* 2020;**227**:191-198.e3. doi:10.1016/j.jpeds.2020.08.038
24 Takacs ZK, Kassai R. The efficacy of different interventions to foster children's executive function skills: A series of meta-analyses. *Psychol Bull* 20190429;**145**:653. doi:10.1037/bul0000195
25 Ehrler M, Naef N, Tuura RO, *et al.* Executive function and brain development in adolescents with severe congenital heart disease (Teen Heart Study): protocol of a prospective cohort study. *BMJ Open* 2019;**9**:e032363. doi:10.1136/bmjopen-2019-032363
26 Kukartz U. *Qualitative Inhaltsanalyse. Methoden, Praxis, Computerunterstützung*. 4. überarbeitete Auflage. 2018.
27 Gordon T, Helmer O. REPORT ON A LONG-RANGE FORECASTING STUDY. 1964. https://apps.dtic.mil/sti/citations/AD0607777 (accessed 23 Jan 2023).

For peer review only - http://bmjopen.bmj.com/site/about/guidelines.xhtml

28 Hasson F, Keeney S, McKenna H. Research guidelines for the Delphi survey technique. 2000;**32**:1008–15.

29 Twiss BC. The Delphi method—Techniques & applications: edited by H. A. Linstone and M. Turoff, Addison-Wesley, Reading, Mass. (1975), 620 pp. $29.50 (hardback) and $16.50 (softback), also by West End House, 11 Hill's Place, London. *Long Range Plann* 1976;**9**:94–5. doi:10.1016/0024-6301(76)90044-3

30 Scheibe M, Skutsch M, Schofer J. *The Delphi Method: Techniques and Applications*. London Reading, Mass.: Addison-Wesley 1975.

31 Drzewiecki CM, Juraska JM. The structural reorganization of the prefrontal cortex during adolescence as a framework for vulnerability to the environment. *Pharmacol Biochem Behav* 2020;**199**:173044. doi:10.1016/j.pbb.2020.173044

32 Teffer K, Semendeferi K. Chapter 9 - Human prefrontal cortex: Evolution, development, and pathology. In: Hofman MA, Falk D, eds. *Progress in Brain Research*. Elsevier 2012. 191–218. doi:10.1016/B978-0-444-53860-4.00009-X

33 Long SH, Eldridge BJ, Harris SR, *et al.* Challenges in Trying to Implement an Early Intervention Program for Infants with Congenital Heart Disease. *Pediatr Phys Ther* 2015;**27**:38–43. doi:10.1097/PEP.0000000000000101

34 Cognifit. Cognifit Inc © 2022. 2022.

35 Dawson P, Guare R. *Schlau aber...* 1st ed. Bern: : Verlag Hans Huber, Hogrefe AG 2012.

36 Liebers A, Kubesch S. *Ein Ritter in der Klasse*. 1st ed. Verlag Bildung Plus 2013.

37 Liebers A, Kubesch S. *Mathetest und Drachenhörnchen*. 1st ed. Verlag Bildung Plus 2013.

38 Liebers A, Kubesch S. *Ob Gespenter Fußball spielen?* 1st ed. Verlag Bildung Plus 2014.

39 Liebers A, Kubesch S. *Stopp oder es kracht!* Verlag Bildung Plus 2014.

40 Delis DC, Kaplan E, Kramer JH. Delis-Kaplan Executive Function System. APA PsycNet. 2001.https://psycnet.apa.org/doiLanding?doi=10.1037%2Ft15082-000 (accessed 19 Jan 2022).

41 Zimmermann P, Fimm B. *Applied Neuropsychology of Attention: Theory, Diagnosis and Rehabilitation*. Psychology Press 2004.

42 Wechsler D. Study Citation Wechsler, D. (2014). WISC-V: Technical and Interpretive Manual. Bloomington. 2014.

43 Lejuez CW, Aklin W, Daughters S, *et al.* Reliability and Validity of the Youth Version of the Balloon Analogue Risk Task (BART–Y) in the Assessment of Risk-Taking Behavior Among Inner-City Adolescents. *J Clin Child Adolesc Psychol* 2007;**36**:106–11. doi:10.1080/15374410709336573

44 Gioia GA, Isquith PK, Guy SC, *et al.* Behavior Rating Inventory of Executive Function®, Second Edition (BRIEF®2). 2000;:2.

45 R Core Team. R: A language and environment for statistical computing. Vienna, Austria: 2020. https://www.R-project.org/

46 Wright A. REDCap: A Tool for the Electronic Capture of Research Data. *J Electron Resour Med Libr* 2016;**13**:197–201. doi:10.1080/15424065.2016.1259026

47 Bustamante JC, Navarro J-J. Technological Tools for the Intervention and Computerized Dynamic Assessment of Executive Functions. Handb. Res. Neurocognitive Dev. Exec. Funct. Implic. Interv. 2022;:310–39. doi:10.4018/978-1-7998-9075-1.ch014

48 Rachanioti E, Bratitsis T, Alevriadou A. Cognitive games for children's Executive Functions Training with or without learning difficulties: an Overview. In: *Proceedings of the 8th International Conference on Software Development and Technologies for Enhancing Accessibility and Fighting Info-exclusion*. Thessaloniki Greece: : ACM 2018. 165–71. doi:10.1145/3218585.3218665

49 Horowitz-Kraus T. Differential Effect of Cognitive Training on Executive Functions and Reading Abilities in Children With ADHD and in Children With ADHD Comorbid With Reading Difficulties. *J Atten Disord* 2015;**19**:515–26. doi:10.1177/1087054713502079

50 Leiss U. Die neuropsychologische Ergebnisbesprechung als Intervention – Eine Brücke zwischen Befund und Alltag. In: Pletschko T, Leiss U, Pal-Handl K, *et al.*, eds. *Neuropsychologische Therapie mit Kindern und Jugendlichen: Praktische Behandlungskonzepte bei neurokognitiven Funktionsstörungen*. Berlin, Heidelberg: : Springer 2020. 35–43. doi:10.1007/978-3-662-59288-5_4

51 Dawson P, Guare R. *Smart But Scattered: The Revolutionary "executive Skills" Approach to Helping Kids Reach Their Potential*. Guilford Press 2009.

52 Pletschko T, Leiss U, Pal-Handl K, *et al. Neuropsychologische Therapie mit Kindern und Jugendlichen: Praktische Behandlungskonzepte bei neurokognitiven Funktionsstörungen*. Springer-Verlag 2020.

53 Bowen DJ, Kreuter M, Spring B, *et al.* How We Design Feasibility Studies. *Am J Prev Med* 2009;**36**:452–7. doi:10.1016/j.amepre.2009.02.002

54 Feely M, Seay KD, Lanier P, *et al.* Measuring Fidelity in Research Studies: A Field Guide to Developing a Comprehensive Fidelity Measurement System. *Child Adolesc Soc Work J* 2018;**35**:139–52. doi:10.1007/s10560-017-0512-6

55 Modi AC, Gutierrez-Colina AM, Wagner JL, *et al.* Executive functioning phenotypes in youth with epilepsy. *Epilepsy Behav* 2019;**90**:112–8. doi:10.1016/j.yebeh.2018.11.026

56 Reeve J, Jang H, Carrell D, *et al.* Enhancing Students' Engagement by Increasing Teachers' Autonomy Support. *Motiv Emot* 2004;**28**:147–69. doi:10.1023/B:MOEM.0000032312.95499.6f

57 Vazou S, Klesel B, Lakes KD, *et al.* Rhythmic Physical Activity Intervention: Exploring Feasibility and

For peer review only

For peer review only - http://bmjopen.bmj.com/site/about/guidelines.xhtml

Effectiveness in Improving Motor and Executive Function Skills in Children. *Front Psychol* 2020;**11**.https://www.frontiersin.org/article/10.3389/fpsyg.2020.556249 (accessed 19 Jan 2022).
58 Duncan B, Miller S, Sparks J, *et al.* The Session Rating Scale: Preliminary Psychometric Properties of a “Working” Alliance Measure. *J Brief Ther* 2003;**3**:3–12.
59 Hoge RD, Andrews DA, Faulkner P, *et al.* The family relationship index: Validity data. *J Clin Psychol* 1989;**45**:897–903. doi:10.1002/1097-4679(198911)45:6<897::AID-JCLP2270450611>3.0.CO;2-T
60 Ravens-Sieberer U. The KIDSCREEN questionnaires. Quality of life quiestionners for children and adolescents - handbook. 2006.
61 Lidzba K, Chrstiansen H, Drechsler R. Conners Skalen zu Aufmerksamkeit und Verhalten - 3. Deutschsprachige Adaptation der Conners 3rd Edition® (Conners 3®) von C. Keith Conners. 2013.
62 Bölte S, Poustka F. Skala zur Erfassung sozialer Reaktivität - Dimensionale Autismus-Diagnostik. Deutsche Fassung der Scoial Responsiveness Scale (SRS) von John N. Constantino und Christian P. Gruber. 2007.
63 Leppert K, Koch B, Brähler E, *et al.* Resilience scale - evaluation of a long (RS-25) and a short version (RS-13). *Klin Diagn Eval* 2008.
64 Largo RH, Pfister D, Molinari L, *et al.* Significance of prenatal, perinatal and postnatal factors in the development of AGA preterm infants at five to seven years. *Dev Med Child Neurol* 1989;**31**:440–56.
65 Eldridge SM, Lancaster GA, Campbell MJ, *et al.* Defining Feasibility and Pilot Studies in Preparation for Randomised Controlled Trials: Development of a Conceptual Framework. *PLOS ONE* 2016;**11**:e0150205. doi:10.1371/journal.pone.0150205
66 Sim J, Lewis M. The size of a pilot study for a clinical trial should be calculated in relation to considerations of precision and efficiency. *J Clin Epidemiol* 2012;**65**:301–8. doi:10.1016/j.jclinepi.2011.07.011
67 Billingham SA, Whitehead AL, Julious SA. An audit of sample sizes for pilot and feasibility trials being undertaken in the United Kingdom registered in the United Kingdom Clinical Research Network database. *BMC Med Res Methodol* 2013;**13**:104. doi:10.1186/1471-2288-13-104
68 RStudio Team. RStudio: Integrated Development Environment for R. 2021.http://www.rstudio.com/

Figure title

Figure 1. Flowchart of intervention development and trial design

For peer review only

For peer review only - http://bmjopen.bmj.com/site/about/guidelines.xhtml

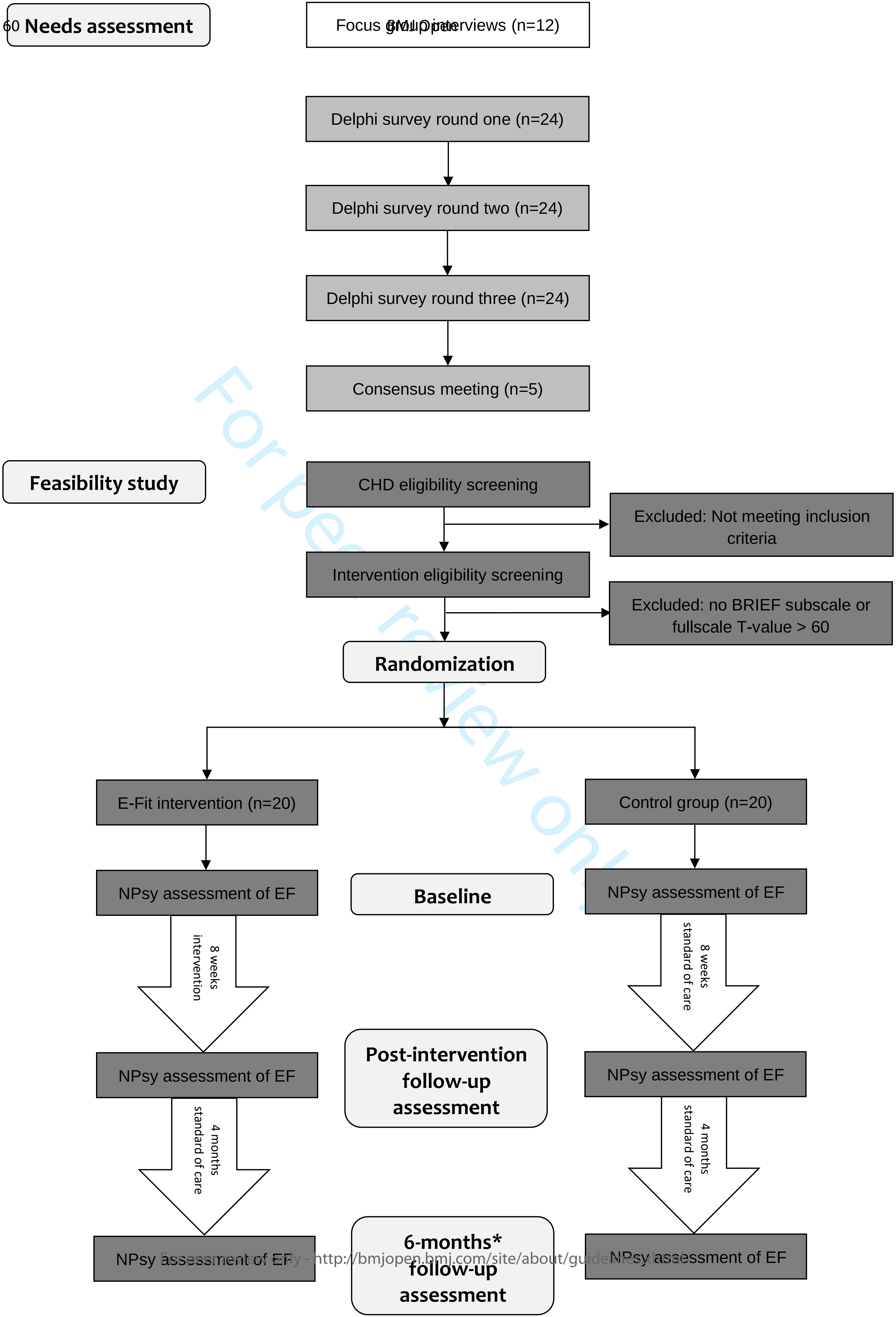
Needs assessment
Focus group interviews (n=12)
Delphi survey round one (n=24)
Delphi survey round two (n=24)
Delphi survey round three (n=24)
Consensus meeting (n=5)
Feasibility study
CHD eligibility screening
Excluded: Not meeting inclusion criteria
Intervention eligibility screening
Excluded: no BRIEF subscale or fullscale T-value > 60
Randomization
E-Fit intervention (n=20)
Control group (n=20)
NPsy assessment of EF
Baseline
NPsy assessment of EF
8 weeks intervention
8 weeks standard of care
NPsy assessment of EF
Post-intervention follow-up assessment
NPsy assessment of EF
4 months standard of care
4 months standard of care
NPsy assessment of EF
6-months* follow-up assessment
NPsy assessment of EF

UNIVERSITY CHILDREN'S HOSPITAL ZURICH

The hospital of the Eleonore Foundation

Focus group November

Discussion			
**Target group**	☐ Parents ☒ Children		
**Date / time / place**	23.11.2020	18.00-19.00	Zoom
**Lead**	Alenka Schmid, Interviewer 2, Interviewer 3		
**Topic**	☒ Intervention		
**Materials / Tools**	• Dodelido (Spiel)<br>• Matches	•	•
**Preparations**	Before:<br>• PowerPoint slides<br>• Create evaluation on Google Docs<br>• Create Collaboard Account (Interviewers: https://web.collaboard.app/projects)<br>• Ensure good lighting	During:<br>• Create Breakoutrooms	

Time	Description	Moderator
18.00-18.10	**Introduction**<br><br>Short greeting, introduction (first names), arriving and rename in Zoom<br>Explain why they are here today<br>Gallery view, mute when not speaking<br>Raise hand<br>Introductions (which animal would I like to be?)	Alenka
18.10-18.20<br><br>18:20-18:30<br><br>18:30-18:40	**Main part**<br><br>Introduction and info about brain training for EF and discussion framework via PowerPoint:<br><br>Different scenarios are presented (shopping and then forgetting, etc.). Children can stretch out if this has happened to them before.<br><br>In a second step, they are asked to stretch out where they feel it happens to them more often than others.<br><br>Possible scenarios<br>- Anger/emotion control<br>- Planning/remembering (https://www.youtube.com/watch?v=o4zbeBcIAtU)<br>- Distractedness (https://www.youtube.com/watch?v=xhHkk1q04GA)<br>- Forgetfulness (https://www.youtube.com/watch?v=9TfJYgnXSus)<br><br>Presenting different possibilities<br>There is something different in each break-out room. Afterwards, the participants are mixed together so that someone from each group joins for the Disney method.<br>These are the following items:<br><br>1. Rubiks Cage (watch video, game with matches)	Alenka<br><br>Alenka<br><br>Interviewer 2

For peer review only - http://bmjopen.bmj.com/site/about/guidelines.xhtml

UNIVERSITY
CHILDREN'S HOSPITAL
ZURICH

The hospital of the Eleonore Foundation

	2. Cogmed (https://www.pearsonclinical.co.uk/Education/BestsellingInterventions/Cogmed/CogmedWorkingMemoryTraining.aspx; jeder für sich)<br>3. Dodelido<br><br>Disney Method (Breakout Rooms)	Alenka<br><br>Interviewer 3<br><br>Interviewer 3, Interviewer 2
18:40-19:00		

Dreamer	Participants are allowed to share and discuss their craziest ideas	5min
Critic	The participants reflect on what is feasible from their crazy ideas.	5min
Realist	The participants try to adapt their ideas so that they still have something to do with their dreams, but are more feasible from their point of view. Here they are also welcome to bring in their experiences from the previous breakout rooms.	10min

19:00	Return from Breakout rooms	
19:00-19:05	**Closing**<br><br>Feedback (fill in online) and acknowledgement<br><br>Questions?	Alenka

Goal of discussion?

	• Needs assessment
**Safety considerations**	• The event will be conducted online via Zoom

Details:

https://www.youtube.com/watch?v=xlqUjpPxcTk
Keep matches ready
Google form for evaluation:

- *In what time frame should the training take place (2 weeks/3 months)?*
- *How much time can the child (and parents) invest each day?*
- *Where should the training take place (at home, at school, at KiSpi, alone, in a group, with parents)?*
- *By what media (paper tasks, computer games, board games)?*
- *How much can or do you want parents to be involved in the training?*

For peer review only

For peer review only - http://bmjopen.bmj.com/site/about/guidelines.xhtml

UNIVERSITY CHILDREN'S HOSPITAL ZURICH

The hospital of the Eleonore Foundation

Focus group November

Discussion			
**Target group**	☒ Parents ☐ Children		
**Date / time / place**	23.11.2020	20.00-21.00	Zoom
**Lead**	Bea Latal, Alenka Schmid		
**Topic**	☒ Intervention		
**Materials / Tools**	• Dodelido<br>• Matches	•	•
**Preparations**	Before:<br>• PowerPoint slides	During:	

Time	Description	Moderator
	**Introduction**	
20.00-20.05	Short greeting, arriving (and rename on Zoom).	Bea
	Introductions (name and name of child, favorite vacation spot), write name and corona front to back.	Alenka
20:05-20:15	Participants are given the link to a Google Survey (or Wordcloud) via Zoom Chat. In it, they can openly write down their thoughts on various topics.<br>Topics:<br>Expectations for tonight<br>Particularly positive characteristics of your child?<br>Everyday problems of the child and with the child?<br>Wishes for further development of the child?	Bea
	**Main part**	
20.15-20.25	Introduction and information about EF at CHD and discussing framework via PowerPoint.	Alenka
20:25-20:35	Introductory question:<br>- Do you feel like a training of the executive functions could help your child?	
	Afterwards, the discussion is let run its course and moderated, if necessary, further questions are introduced.<br>Possible questions:<br><br>- What problems does your child have in everyday life?<br>- What do you expect from the training?<br>- What do you expect from the people who offer and conduct training?<br>- In what time frame should a training take place. How much time can the child and parents invest?<br>- At what age should it start?<br>- Where should the training take place, by what media? Paper tasks, computer games, alone, in group, at home, at the children's hospital?<br>- How much can or do you as parents want to participate in the training?<br>- Do you think your child would be motivated to do such a training?<br>- Are there other things for which training/intervention would be useful? (E.g., stress management, social graces, understanding the illness, exercise and physical activity?)	Bea

For peer review only - http://bmjopen.bmj.com/site/about/guidelines.xhtml

UNIVERSITY
CHILDREN'S HOSPITAL
ZURICH

The hospital of the Eleonore Foundation

20:35-20:55	Presentation of the team's ideas or direct demonstration (of the same things as to the children) and discussion of them	Alenka
20:55-21:00	**Closing**<br>Google Doc for evaluation<br>Acknowledgement and closing	 Alenka<br>Bea

Goal of discussion?

	• Needs assessment
**Safety considerations**	• The event will be conducted online via Zoom

For peer review only

For peer review only - http://bmjopen.bmj.com/site/about/guidelines.xhtml

A) Development of the Delphi survey

Sentences derived by qualitative content analysis from the focus groups were sorted into nine categories. Then, the statements were summarized and finally used to define the items in the Delphi survey. An example of statements and the consequent items is given in table S6. The items are deliberately positively formulated. Some items were highly specific to children with CHD. Hence, the items for the “identity” and “dealing with the disease” categories were dropped for the Delphi survey. The final set of items included in the Delphi survey is provided in figure S1.

B) Rationale behind the final intervention design

B.1) Virtual and analog games

Another element key to the success of any intervention is motivation [1]. Johann and Karbach compared six computer-based cognitive training programs [2]. They found evidence that playful interventions promote motivation and adherence in typically developing elementary school students. This reinforced the effects achieved by the EF training [2]. In addition, they emphasize the difference between computer-based training with added game elements and regular video games [3]: Videogames such as Super Mario and Fruit Ninja have no effect on EFs. In contrast, computer-based training with added game elements, such as in Lumosity® or CogniFit Inc © 2022 [4,5] shows clear benefits for EF improvement. In computer-based approaches, the training effect is maintained, and motivation is increased [3]. Because an intervention should be enjoyable to keep participants motivated, analog games such as board and card games have also been tested as cognitive training [6,7]. One obstacle associated with interventions using analog games is the mandatory involvement of other family or study members, which might overstrain time resources. However, participants of both the focus group and the Delphi survey indicated that it would be important to include analog games in the intervention. Hence, different analog games were added to the intervention, including games that can be played alone, and this part was decided to be open in terms of frequency and time spent on it. Moreover, whether the intervention should be online or analog was discussed without reaching a consensus by the Delphi survey participants. A systematic review and meta-analysis by Cao et al. found slight-to-moderate evidence for the effectiveness of computer-based interventions in promoting EF [3]. Computer-based interventions have been used in the population of CHD patients previously, and in the Delphi survey, only 4 of 12 participants disagreed with the online format. Hence, we decided that the intervention should include computer-based games. In addition, participating children could also use analog games. We decided to use existing computer-based and analog games instead of creating new games.

The intervention also includes analog and computerized games which, in addition to EF, promote eye-hand coordination, thus meeting a suggestion that motor games be included.

For peer review only

B.2) Strategy Coaching

The most highly rated items were related to learning strategies addressing everyday lives and homework. This is in line with the literature: another substantial shortcoming of many interventional studies is the lack of transfer [8–11]. Transfer refers to the degree of improvement in specific tasks requiring the same cognitive functions, termed near transfer, and in general cognitive abilities, termed far transfer [12]. A way to overcome a lack of transfer is by providing personal coaching sessions that support EF strategies in daily life and take into account children's emotional and social development [8]. Hence, we decided on adding strategy coaching. Career planning was a topic discussed in the Delphi survey. It was decided not to include this topic, as this is not appropriate for an age group of 10–12-year-old children.

B.3) Length, frequency per week and overall duration of the intervention

The most difficult aspect of the development of the intervention related to the minutes per day, frequency per week, and overall duration of the sessions. A wide range of possibilities was suggested (see figure S2). These could not all be met. The length of the computerized training sessions was defined as 20 minutes, which was in agreement with the participants' suggestions. Other studies using session lengths of 20 minutes trained three to four times a week [13,14], which was a higher frequency than suggested by the families. Consequently, we decided on a frequency of three times a week and an overall duration of 8 weeks instead of the 6 months suggested (figure S2).

For peer review only

1 Mohammed S, Flores L, Deveau J, *et al.* The Benefits and Challenges of Implementing Motivational Features to Boost Cognitive Training Outcome. *J Cogn Enhanc* 2017;**1**:491–507. doi:10.1007/s41465-017-0047-y
2 Johann VE, Karbach J. Effects of game-based and standard executive control training on cognitive and academic abilities in elementary school children. *Developmental Science* 2020;**23**:e12866. doi:10.1111/desc.12866
3 Cao Y, Huang T, Huang J, *et al.* Effects and Moderators of Computer-Based Training on Children's Executive Functions: A Systematic Review and Meta-Analysis. *Front Psychol* 2020;**11**:580329. doi:10.3389/fpsyg.2020.580329
4 Lumos Labs, Inc. Lumosity ®. 2022.
5 Cognifit. Cognifit Inc © 2022. 2022.
6 Estrada-Plana V, Esquerda M, Mangues R, *et al.* A Pilot Study of the Efficacy of a Cognitive Training Based on Board Games in Children with Attention-Deficit/Hyperactivity Disorder: A Randomized Controlled Trial. *Games for Health Journal* 2019;**8**:265–74. doi:10.1089/g4h.2018.0051
7 Gonçalves PD, Ometto M, Sendoya G, *et al.* Neuropsychological Rehabilitation of Executive Functions: Challenges and Perspectives. *Journal of Behavioral and Brain Science* 2014;**2014**. doi:10.4236/jbbs.2014.41004
8 Diamond A, Lee K. Interventions Shown to Aid Executive Function Development in Children 4 to 12 Years Old. *Science* Published Online First: 19 August 2011. doi:10.1126/science.1204529
9 Thorell LB, Lindqvist S, Bergman Nutley S, *et al.* Training and transfer effects of executive functions in preschool children. *Developmental Science* 2009;**12**:106–13. doi:10.1111/j.1467-7687.2008.00745.x
10 Diamond A, Ling DS. Conclusions about interventions, programs, and approaches for improving executive functions that appear justified and those that, despite much hype, do not. *Developmental Cognitive Neuroscience* 2016;**18**:34–48. doi:10.1016/j.dcn.2015.11.005
11 Gunzenhauser C, Nückles M. Training Executive Functions to Improve Academic Achievement: Tackling Avenues to Far Transfer. *Frontiers in Psychology* 2021;**12**.https://www.frontiersin.org/article/10.3389/fpsyg.2021.624008 (accessed 11 Mar 2022).
12 Barnett SM, Ceci SJ. When and where do we apply what we learn?: A taxonomy for far transfer. *Psychological Bulletin* 2002;**128**:612–37. doi:10.1037/0033-2909.128.4.612
13 Greco G. MULTILATERAL TRAINING USING PHYSICAL ACTIVITY AND SOCIAL GAMES IMPROVES MOTOR SKILLS AND EXECUTIVE FUNCTION IN CHILDREN WITH AUTISM SPECTRUM DISORDER. *European Journal of Special Education Research* Published Online First: 16 March 2020. doi:10.46827/ejse.v0i0.2928
14 Ramezan Saatchi L, Khodabakhsh Pirkalani R, Moradisabzevar M, *et al.* The effect of computerized Cognitive Training package on executive functions of people with major depressive disorder. *Iranian Journal of Psychiatric Nursing* 2022;**10**:39–52. doi:10.22034/IJPN.10.3.39

For peer review only

UNIVERSITÄTS-KINDERSPITAL ZÜRICH
Forschungszentrum für das Kind FZK

Einwilligungserklärung

Schriftliche Einwilligungserklärung zur Teilnahme an einer klinischen Studie
Bitte lesen Sie dieses Formular sorgfältig durch. Bitte fragen Sie, wenn Sie etwas nicht verstehen oder wissen möchten. Für die Teilnahme ist Ihre schriftliche Einwilligung notwendig.

**BASEC-Nummer (nach Einreichung):**	
**Titel der Studie (wissenschaftlich und Laiensprache)**:	Executive functions intervention in children and adolescents with severe congenital heart disease: a feasibility study (E-Fit)<br><br>Intervention bei Kindern und Jugendlichen mit schweren angeborenen Herzfehlern: eine Machbarkeitsstudie (E-Fit)
**Verantwortliche Institution (Sponsor mit Adresse)**:	Prof. Dr. med. Bea Latal<br>Abteilung für Entwicklungspädiatrie<br>Universitäts-Kinderspital Zürich<br>Steinwiesstrasse 75, 8032 Zürich
**Ort der Durchführung**:	Universitäts-Kinderspital Zürich
**Prüfärztin/Prüfarzt am Studienort:**<br>Name und Vorname in Druckbuchstaben:	Prof. Dr. med. Bea Latal
**Teilnehmerin/Teilnehmer:**<br>Name und Vorname in Druckbuchstaben:<br>Geburtsdatum:	____________________<br>____________________<br>☐ weiblich ☐ männlich

- Ich und mein Kind wurden von der unterzeichnenden Studienkoordination mündlich und schriftlich über den Zweck, den Ablauf der Studie mit E-Fit über mögliche Vor- und Nachteile sowie über eventuelle Risiken informiert.
- Ich und mein Kind nehmen an dieser Studie freiwillig teil und akzeptiere den Inhalt der mir ausgehändigten schriftlichen Information. Ich und mein Kind hatten genügend Zeit, unsere Entscheidung zu treffen.
- Meine Fragen und die meines Kindes im Zusammenhang mit der Teilnahme an dieser Studie sind uns beantwortet worden. Ich und mein Kind behalten die schriftliche Information und erhalten eine Kopie unserer schriftlichen Einwilligungserklärung.
- Ich und mein Kind sind einverstanden, dass die Kinderärztin/der Kinderarzt meines Kindes über die Teilnahme an der Studie informiert wird.
- Ich und mein Kind sind einverstanden, dass die zuständigen Fachleute des Sponsors, der zuständigen Ethikkommission zu Prüf- und Kontrollzwecken in unsere unverschlüsselten Daten Einsicht nehmen dürfen, jedoch unter strikter Einhaltung der Vertraulichkeit.
- Bei Ergebnissen (und/oder Zufallsbefunden), die direkt die Gesundheit meines Kindes betreffen, werden ich und mein Kind informiert. Wenn ich und mein Kind das nicht wünschen, informieren wir die Studienkoordination.
- Ich und mein Kind wissen, dass die gesundheitsbezogenen und persönlichen Daten meines Kindes nur in verschlüsselter Form zu Forschungszwecken für diese Studie (auch im Ausland)

For peer review only - http://bmjopen.bmj.com/site/about/guidelines.xhtml
For peer review only

TEEN HEART STUDY E-FIT

UNIVERSITÄTS-KINDERSPITAL ZÜRICH
Forschungszentrum für das Kind FZK

weitergegeben werden können. Der Sponsor gewährleistet, dass der Datenschutz nach Schweizer Standard eingehalten wird.

- Ich und mein Kind können jederzeit und ohne Angabe von Gründen von der Studienteilnahme zurücktreten. Die weitere medizinische Behandlung meines Kindes ist unabhängig von der Studienteilnahme gewährleistet. Die bis zum Rücktritt erhobenen Daten werden noch im Rahmen der Studie ausgewertet.
- Die Haftpflichtversicherung Universitäts-Kinderspital Zürich kommt für allfällige Schäden auf.
- Bei Zufallsbefunden möchten wir

  a) ☐ in jedem Fall informiert werden
  b) ☐ nicht informiert werden
  c) ☐ die Entscheidung folgender Person überlassen: ....

- Ich und mein Kind sind damit einverstanden, dass die Studienleitenden den aktuellsten kardiologischen Befund beim/bei der zuständigen Kardiolog:in einholen, um den Gesundheitszustand meines Kindes einzuschätzen.

  Name Kardiologe:___________________________

  Name Klinik: _______________________________

- Ich bin damit einverstanden, dass persönliche Daten von mir im Rahmen dieser Studie ausgewertet und verschlüsselt and Drittpersonen innerhalb des Universitäts-Kinderspitals Zürich weitergegeben werden können.
  ☐ Ja ☐ Nein

- Ich und mein Kind sind damit einverstanden, dass persönliche Daten verschlüsselt an Drittpersonen innerhalb des Universitäts-Kinderspitals Zürich weitergegeben werden können.
  ☐ Ja ☐ Nein

- Ich und mein Kind sind damit einverstanden, dass die Daten aus der neuropsychologischen Abklärung verschlüsselt zu Forschungszwecken an CogniFit© weitergegeben werden können.
  ☐ Ja ☐ Nein

- Ich und mein Kind sind damit einverstanden, dass während der neuropsychologischen Abklärung und der Intervention Videoaufnahmen gemacht und verschlüsselt abgespeichert werden.
  ☐ Ja ☐ Nein

- Ich und mein Kind sind damit einverstanden, dass die Hauptlehrperson über die Studienteilnahme informiert und miteinbezogen wird.
  ☐ Ja ☐ Nein

For peer review only - http://bmjopen.bmj.com/site/about/guidelines.xhtml

Die unterzeichnenden Personen bezeugen, dass das Aufklärungsgespräch stattgefunden hat und dass das urteilsfähige Kind in die Studienteilnahme eingewilligt hat und/oder sich keine Anzeichen von Widerstand gegen die Teilnahme erkennen lassen.

Ort, Datum	Unterschrift gesetzliche Vertretung des Kindes

Bestätigung der Studienkoordination: Hiermit bestätige ich, dass ich dieser Teilnehmerin/diesem Teilnehmer Wesen, Bedeutung und Tragweite der Studie erläutert habe. Ich versichere, alle im Zusammenhang mit dieser Studie stehenden Verpflichtungen gemäss in der Schweiz geltenden Rechts zu erfüllen. Sollte ich im Verlauf der Studie von Aspekten erfahren, welche die Bereitschaft der Teilnehmerin/des Teilnehmers zur Studienteilnahme beeinflussen könnten, werde ich sie/ihn umgehend darüber informieren.

Ort, Datum	Name und Vorname der Studienkoordination in Druckbuchstaben<br><br>Unterschrift der Studienkoordination

For peer review only - http://bmjopen.bmj.com/site/about/guidelines.xhtml

A) Computerized training (CogniFit Inc © 2022)

It is important to note that a contract was concluded with CogniFit Inc © 2022, because they provide discounts on their services in exchange for the pseudonymized baseline assessment data. Families had the choice whether to provide their data or not.

B) Strategy coaching

At the beginning of the intervention, parents complete a short questionnaire [1], assessing the behavior of their child in everyday life. The questionnaire was designed for parents to identify EF domains in which their children face difficulties. Lower scores indicate greater difficulties. Out of eleven EF domains rated, the six lowest scored are selected for the coaching sessions. Possible topics are working memory, attention control, emotion regulation, flexibility, initiating action, metacognition, organization, planning, response inhibition, time management and goal-directed persistence. In the first coaching session, the child's performance in the EF baseline assessment are discussed, and strengths and difficulties are identified [2]. Each of the next six sessions focuses on one of the six EF domains identified as most affected [1]. At the beginning of each session, the previous week is reviewed, focusing on the EF intervention and coaching instructions given at the previous session. Also, difficulties in regard to adherence are discussed and solutions suggested by the coach. In the second part of the session, a short story is read, featuring protagonists dealing with similar challenge as is the topic of that coaching session [3–6]. In the third part of the session, the focus is transferred to the child's situation by asking about how they could handle similar situations. Further, the child completes a task for the coaching session. Templates for these are available in Dawson et al.'s book *Smart but scattered* [1] and include checklist, plans, and tables. At the end of the session, the child is encouraged to use these strategies the following week, and the parents are informed by email about these strategies after the session. The parents receive fact sheets regarding the discussed EF domain of that particular session with tips for everyday life and a short summary on what was done in the session. All parental tips are also taken from the German version of *Smart but scattered* [1].

For peer review only - http://bmjopen.bmj.com/site/about/guidelines.xhtml

1 Dawson P, Guare R. *Schlau aber...* 1st ed. Bern: : Verlag Hans Huber, Hogrefe AG 2012.

2 Leiss U. Die neuropsychologische Ergebnisbesprechung als Intervention – Eine Brücke zwischen Befund und Alltag. In: Pletschko T, Leiss U, Pal-Handl K, *et al.*, eds. *Neuropsychologische Therapie mit Kindern und Jugendlichen: Praktische Behandlungskonzepte bei neurokognitiven Funktionsstörungen*. Berlin, Heidelberg: : Springer 2020. 35–43. doi:10.1007/978-3-662-59288-5_4

3 Liebers A, Kubesch S. *Ob Gespenter Fußball spielen?* 1st ed. Verlag Bildung Plus 2014.

4 Liebers A, Kubesch S. *Mathetest und Drachenhörnchen*. 1st ed. Verlag Bildung Plus 2013.

5 Liebers A, Kubesch S. *Ein Ritter in der Klasse*. 1st ed. Verlag Bildung Plus 2013.

6 Liebers A, Kubesch S. *Stopp oder es kracht!* Verlag Bildung Plus 2014.

For peer review only

TEEN HEART STUDY E-FIT

Acceptance and Feasibility Scale Parents

	Fully disagree			Fully agree
The program was useful for my child.	☐	☐	☐	☐
My child would do the program again.	☐	☐	☐	☐
I would recommend this program to other children.	☐	☐	☐	☐
My child has applied the skills learned.	☐	☐	☐	☐
My child has a plan for dealing with future problems.	☐	☐	☐	☐
My child has made positive changes.	☐	☐	☐	☐
My child has used the skills to become more organized.	☐	☐	☐	☐
My child has used reminder strategies to remember things more often.	☐	☐	☐	☐
My child uses techniques to plan.	☐	☐	☐	☐
My child has met his or her program goals.	☐	☐	☐	☐
My child has a more positive attitude than before.	☐	☐	☐	☐
My child is better at solving problems.	☐	☐	☐	☐
My child remembers things better.	☐	☐	☐	☐
My child is more organized.	☐	☐	☐	☐
My child is better at paying attention and remembering things.	☐	☐	☐	☐
My child is better at getting things done, such as homework or chores.	☐	☐	☐	☐
My child is better at controlling his or her emotions.	☐	☐	☐	☐
My child is better at controlling his or her actions.	☐	☐	☐	☐
My child is better at stopping and thinking before acting.	☐	☐	☐	☐
My child is better able to handle unforeseen changes in plans.	☐	☐	☐	☐
My child feels less stressed.	☐	☐	☐	☐
My child does better in school.	☐	☐	☐	☐

For peer review only - http://bmjopen.bmj.com/site/about/guidelines.xhtml
adapted after Modi et al., 2019

TEEN HEART STUDY
E-FIT

Acceptance and Feasibility Scale Children

	Fully disagree			Fully agree
Everything worked as it should.	☐	☐	☐	☐
It was easy to use the online website for the games.	☐	☐	☐	☐
I liked the online games.	☐	☐	☐	☐
It was easy to get in touch with the coach through Microsoft Teams/Zoom.	☐	☐	☐	☐
Meeting with a coach in person would be better.	☐	☐	☐	☐
I felt connected to the coach.	☐	☐	☐	☐
I felt that the coach contributed to my learning beyond the sessions.	☐	☐	☐	☐
The information from the coaching sessions was helpful to me.	☐	☐	☐	☐
I enjoyed the coaching sessions.	☐	☐	☐	☐
I enjoyed the analogue games.	☐	☐	☐	☐
It was easy to understand the game instructions (videos and or brain cards).	☐	☐	☐	☐
The program (as a whole) was too long.	☐	☐	☐	☐
The program (as a whole) was too short.	☐	☐	☐	☐
The program (as a whole) was as I expected it to be.	☐	☐	☐	☐
I liked the program (as a whole).	☐	☐	☐	☐
The content was not relevant to me.	☐	☐	☐	☐
The program (as a whole) was useful to me.	☐	☐	☐	☐
I would do the program (as a whole) again.	☐	☐	☐	☐
I would recommend the program (as a whole) to other children.	☐	☐	☐	☐
What part of the program did you enjoy the most?	☐	☐	☐	☐
What part of the program helped you the most?	☐	☐	☐	☐

	no	yes		
Could any part be dropped?	☐	☐	If yes which one?	

TEEN HEART STUDY
E-FIT

	Fully disagree			Fully agree
I applied the skills I learned during the program.	☐	☐	☐	☐
I have a plan for dealing with future problems.	☐	☐	☐	☐
I made positive changes.	☐	☐	☐	☐
I have used skills to get more organized.	☐	☐	☐	☐
I have used remembering strategies to remember things better.	☐	☐	☐	☐
I have used techniques to plan.	☐	☐	☐	☐
I have achieved the goals I had at the beginning of the program.	☐	☐	☐	☐
I have a more positive attitude than before.	☐	☐	☐	☐
I am better at solving problems.	☐	☐	☐	☐
I am better at remembering things.	☐	☐	☐	☐
I am more organized.	☐	☐	☐	☐
I know strategies that help me pay attention and remember.	☐	☐	☐	☐
I start things, like homework or chores, better than I used to.	☐	☐	☐	☐
I can control my emotions better than I used to.	☐	☐	☐	☐
I can control my actions better than I used to.	☐	☐	☐	☐
I am better able to stop and think before I act.	☐	☐	☐	☐
I can better cope with unforeseen changes in plans.	☐	☐	☐	☐
I feel less stressed.	☐	☐	☐	☐
I do better in school.	☐	☐	☐	☐

For peer review only - http://bmjopen.bmj.com/site/about/guidelines.xhtml

Everyday Activities Diary

List of activities: School *1 box = 1 quarter of an hour*
What did your child do today?
Please indicate your child's activities today (to the nearest quarter of an hour)

		06:00	07:00	08:00	09:00	10:00	11:00	12:00
**General**								
Sleeping								
Eating								
**School**								
Lessons								
Physical education								
Recess								
**Leisure time**								
Homework								
Reading or browsing through books on his/her own								
Playing a musical instrument or singing								
Watching TV, videos or DVDs								
Computer or video games, Playstation, Nintendo or the Internet								
Other quite activities or games (listening to music or stories, drawing, painting, handiwork or board games)								
Relatively vigorous games with friends or siblings (playground, role-playing, hide-and-seek, etc.)	Indoor							
	Outdoor							
Vigorous games, running around or ball games (not in a sports club)	Indoor							
	Outdoor							
Training, riding or ballet (in a club)	Indoor							
	Outdoor							
**Travel**								
By foot								
By bicycle, scooter, inline skates or skateboard								
By train, tram or bus								
By car								
**Special**								
Activity difficult to classify								

		12:00	13:00	14:00	15:00	16:00	17:00	18:00	19:00	20:00	21:00	22:00	23:00	24:00	01:00	02:00	03:00	04:00	05:00	06:00
**General**																				
Sleeping																				
Eating																				
**School**																				
Lessons																				
Physical education																				
Recess																				
**Leisure time**																				
Homework																				
Reading or browsing through books on his/her own																				
Playing a musical instrument or singing																				
Watching TV, videos or DVDs																				
Computer or video games, Playstation, Nintendo or the Internet																				
Other quite activities or games (listening to music or stories, drawing, painting, handiwork or board games)																				
Relatively vigorous games with friends or siblings (playground, role-playing, hide-and-seek, etc.)	Indoor																			
	Outdoor																			
Vigorous games, running around or ball games (not in a sports club)	Indoor																			
	Outdoor																			
Training, riding or ballet (in a club)	Indoor																			
	Outdoor																			
**Travel**																				
By foot																				
By bicycle, scooter, inline skates or skateboard																				
By train, tram or bus																				
By car																				
**Special**																				
Activity difficult to classify																				

UNIVERSITÄTS-
KINDERSPITAL
ZÜRICH
Forschungszentrum
für das Kind FZK

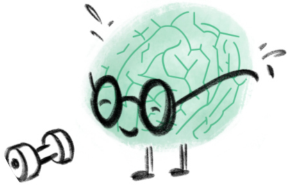

Coaching Content Preparatory Session

Timepoint	Content		Item number
	Explained why we did the assessment	☐ yes ☐ no	1
	Neuroinfo for Kids (NIK) page 3 read aloud	☐ yes ☐ no	2
	Strengths and weaknesses sheet filled in	☐ yes ☐ no	3
	NIK page 5 read aloud	☐ yes ☐ no	4
	NIK page 6 read aloud	☐ yes ☐ no	5
	Results discussed on normal distribution	☐ yes ☐ no	6
	NIK results (2) page read aloud	☐ yes ☐ no	7
	NIK page 10 read aloud	☐ yes ☐ no	8
	Letter written	☐ yes ☐ no	9
	Duration of session 60min	☐ yes ☐ no<br>If no, duration in min: ____________	10

For peer review only

For peer review only - http://bmjopen.bmj.com/site/about/guidelines.xhtml

Coaching Content EF Sessions 1-6

**Timepoint**	**Content**		Item number
	Question asked:<br>Were you able to try out what we talked about last week?	☐ yes ☐ no	10
	Question asked:<br>Did you play the card games? (Which ones?)	☐ yes ☐ no	11
	Question asked:<br>Did everything work out with the online games? I saw you played 3x last week and always started 2x, great! (if necessary point out if something was forgotten)	☐ yes ☐ no	12
	Question asked:<br>Did you add the stickers to your poster?	☐ yes ☐ no	13
	Session topic explained	☐ yes ☐ no	14
	Session goal explained	☐ yes ☐ no	15
	All listed pages of the story read aloud/viewed	☐ yes ☐ no	16
	Assignment completed as described in the session guideline	☐ yes ☐ no	17
	Homework assigned	☐ yes ☐ no	18
	Session duration 60min	☐ yes ☐ no<br>If no, duration in min:<br>__________	19

For peer review only - http://bmjopen.bmj.com/site/about/guidelines.xhtml

Coaching Content Last Session

Timepoint	Content		Item number
	Question asked:<br>Were you able to try out what we talked about last week?	☐ yes ☐ no	10.7
	Question asked:<br>Did you play the card games? (Which ones?)	☐ yes ☐ no	11.7
	Question asked:<br>Did everything work out with the online games? I saw you played 3x last week and always started 2x, great! (if necessary point out if something was forgotten)	☐ yes ☐ no	12.7
	Question asked:<br>Did you add the stickers to your poster?	☐ yes ☐ no	13.7
	Overview of this session provided	☐ yes ☐ no	20
	Summary of the last 8 weeks	☐ yes ☐ no	21
	Evaluation questions asked according to guideline	☐ yes ☐ no	22
	How to continue explained according to guideline	☐ yes ☐ no	23
	Questions and comments of the family clarified	☐ yes ☐ no	24
	Session duration 60min	☐ yes ☐ no<br>If no, duration in min:<br>__________	19.7

For peer review only - http://bmjopen.bmj.com/site/about/guidelines.xhtml

For peer review only

Computerized Games

Date	Content		Item number
	Sessions 1&2 completed	☐ yes ☐ no	25
	Sessions 3&4 completed	☐ yes ☐ no	26
	Sessions 5&6 completed	☐ yes ☐ no	27
	Sessions 7&8 completed	☐ yes ☐ no	28
	Sessions 9&10 completed	☐ yes ☐ no	29
	Sessions 11&12 completed	☐ yes ☐ no	30
	Sessions 13&14 completed	☐ yes ☐ no	31
	Sessions 15&16 completed	☐ yes ☐ no	32
	Sessions 17&18 completed	☐ yes ☐ no	33
	Sessions 19&20 completed	☐ yes ☐ no	34
	Sessions 21&22 completed	☐ yes ☐ no	35
	Sessions 23&24 completed	☐ yes ☐ no	36
	Sessions 25&26 completed	☐ yes ☐ no	37

For peer review only

For peer review only - http://bmjopen.bmj.com/site/about/guidelines.xhtml

Analogue Games

**Duration**	**Content**		Item number
	Gold	☐ yes ☐ no	38
	Dodelido	☐ yes ☐ no	39
	Särge schubsen	☐ yes ☐ no	40
	Geistesblitz 2.0	☐ yes ☐ no	41
	Beaver Gang	☐ yes ☐ no	42
	Beaver Clan	☐ yes ☐ no	43
	Memo Dice	☐ yes ☐ no	44
	5-er finden	☐ yes ☐ no	45
	The nasty 7	☐ yes ☐ no	46
	Yokai	☐ yes ☐ no	47
	Okiya	☐ yes ☐ no	48
	Perplexus Crazy run 2 PE	☐ yes ☐ no	49
	Perplexus Crazy run 5 FT 208	☐ yes ☐ no	50
	Perplexus Crazy run 6 PE Epic	☐ yes ☐ no	51
	Thunder Pops	☐ yes ☐ no	52

For peer review only

For peer review only - http://bmjopen.bmj.com/site/about/guidelines.xhtml

Supplementary information for the SPIRIT Guidelines

A) Table S3 Dataset information

Data category	Information
Primary registry and trial identifying number	ClinicalTrials.gov<br>NCT05198583
Date of registration in primary registry	19 January, 2022
Source(s) of monetary or material support	Swiss National Science Foundation (SNF 32003B_172914)
Contact for public & scientific queries	Prof. Dr. Beatrice Latal; bea.latal@kispi.uzh.ch
Scientific title	Multimodal personalized executive function intervention (E-Fit) for school-age children with complex congenital heart disease: protocol for a randomized controlled feasibility study
Country of recruitment	Switzerland
Health condition(s) or problem(s) studied	Executive dysfunction, severe congenital heart disease
Intervention(s)	Intervention group: executive function intervention (E-Fit)<br>Control group: electronic diary
Key inclusion and exclusion criteria	Ages eligible for study: 10-12 years<br>Sexes eligible for study: both<br>Accepts healthy volunteers: no<br>Inclusion criteria: child born with congenital heart disease (10-12 years), cardiopulmonary bypass surgery prior to one year of age, executive function (EF) deficits<br>Exclusion criteria: heart surgery not performed in Switzerland, known genetic or dysmorphic syndrome
Study type	Interventional allocation: randomized<br>Intervention model: parallel assignment<br>Masking: single blind, outcome assessors<br>Primary purpose: feasibility<br>Phase I
Date of first enrolment	April 2022
Target sample size	40
Recruitment status	Recruiting
Primary Outcome(s)	Feasibility
Key secondary outcomes	Executive function performance

For peer review only

B) Organizational structure and responsibilities

Principal Investigator: Beatrice Latal, Child Development Center, University Children's Hospital Zurich Switzerland

Preparation of protocol and revisions

Publication of study reports

Lead Investigator: Alenka Schmid, Child Development Center, University Children's Hospital Zurich Switzerland

Responsible for identification, recruitment, data collection, and completion of case report forms (CRFs), along with patients adherence to study protocol. Responsible for maintenance of trial IT system and data entry, data verification, and randomization

Responsible for trial master file

Recruitment of patients and liaising with principal investigator

Preparation of CRFs

Design and conduct of E-Fit Feasibility Study

Authorship eligibility guideline

Authors on this paper made a significant contribution to the conception or design of the project or the acquisition, analysis, or interpretation of data for the work, and/or drafted the work or reviewed and revised it critically for important intellectual content.

C) Data management and safety monitoring

Study and questionnaire data are collected by physicians, psychologists, or psychologists in training and recorded in Redcap® [51]. Data from routine visits are derived by a physician from the electronic medical charts of the hospital's data management system, Phoenix, with access limited to the staff of the Children`s Hospital and with strict regulation of data protection. Any prints are locked in a drawer, with access only by the investigator.

Members of the Children's Research Centre of the University Children's Hospital Zurich fulfil the monitoring duties. This includes double checking the data, prestudy monitoring, after enrolment of the 2nd patient, after the 20th patient and a close-out visit. Monitoring covers all study documents, source data, and the trial master file. No interim analyses are planned. This trial is considered as a nonsignificant risk device study and was reviewed accordingly by the Cantonal Ethics Committee of Zurich.

Parents and participants may withdraw from participation at any time. Families are contacted by phone to ascertain the reason for discontinuation as this is important information for the primary outcome of the present study, feasibility. All data collected until drop out will be used for analysis. Serious events and serious adverse events are reported in accordance with the guidelines of the Zurich Cantonal

Ethics Committee, based on the Swiss Federal Act on Research Involving Human Beings. Protocol modifications and amendments will be submitted to the ethical committees for approval. Amendments to the study protocol will be added to publications reporting the study outcomes.

1 Wright A. REDCap: A Tool for the Electronic Capture of Research Data. *J Electron Resour Med Libr* 2016;**13**:197–201. doi:10.1080/15424065.2016.1259026

For peer review only

Table S1 Examples of item construction

Categories	Examples	Summary	Item
1 Identity/EF	*"But what I would certainly like for [my son] is … that he is trained a bit. … What should [he] do first, or how can [he] plan the day."*	Daily planning as an everyday problem.	Children with congenital heart defects structure their everyday life
2 Identity/Relation to peers	*"he needs more time and he's a bit slower and then he sees [his] siblings [and] that they're always faster, and then he always makes himself small and that's a shame."*	Is slower than Peers which affects him negatively.	Children with congenital heart defects feel equal compared to their siblings/peers
3 Scope of intervention/game suggestions	*"analog games would also be OK, [I]can imagine that."*	Analog games.	Dice games should be part of such an intervention.
4 Scope of intervention/structure	*"we would like it if it were possible online, because travelling … is always- … a bit far, yes. Especially during school time"*	Online is better for families who live further away from the hospital.	The intervention should be conducted online
5 Scope of intervention/possible distracting factors	*"they just have … a lot of homework …, then he plays the cello, then he goes to the youth club, everything interests him."*	Other hobbies also need a lot of time and should still have room.	Hobbies could get in the way of the intervention
6 Scope of intervention/possible supporting factors	*"That it [should not be like] 'oh that in addition,' but 'great, … that would be great right now and is manageable in terms of time'."*	Time-manageable and fun.	This is the maximum amount of time per day the participants should work on the intervention (in free time):
7 Dealing with the disease/challenges	*"[that] is … something that … she finds quite difficult and where she probably swallows a lot."*	She finds it difficult to talk about her illness and swallows a lot.	Children with congenital heart defects talk about their heart defect
8 Dealing with the disease/supporting factors	*"contact with other heart children, is certainly motivating."*	Contact with other heart children can be motivating.	During the intervention, the children and adolescents should have contact with other children and adolescents
9 Further ideas	*"I think [contact with other children] will motivate them more than if they do their exercise in their corner [or] maybe talk to someone from your team from time to time"*	Contact with other children with CHD or with the study team	In such an intervention, there should be face-to-face meetings with the study team

For peer review only - http://bmjopen.bmj.com/site/about/guidelines.xhtml

Table S2 Timeline of the study protocol

	Study timeline				
	**Enrolment**	**Allocation**	**Post-Allocation**		
**TIMEPOINT**			***Baseline***	***+ 2 months***	***+ 6 months***
**ENROLMENT:**					
**Informed consent**	x				
**Eligibility screen**	x				
**Allocation**		x			
**INTERVENTIONS:**					
E-Fit Intervention			↔	↔	
Daily Diary			↔	↔	
**ASSESSMENTS:**					
IQ			x		
EF assessment			x	x	x
Questionnaires			x	x	x
Feasibility	x	x	↔	↔	
SES			x		
Medical information			x		

Note : EF = Executive function; SES = socioeconomic status

For peer review only

Table S3 Example week for a participating family

	Exemplar week					
	MO	***TUE***	***WED***	***THU***	***FRI***	***WEEKEND***
**E-FIT INTERVENTION**						
Parents	30min Call					
Child	60min Coaching Session[a]	20min CogniFit[b]		20min CogniFit[b]	20min CogniFit[b]	Card games
**CONTROL GROUP**						
Parents	10min Diary		10min Diary		10min Diary	10min Diary
Child	10min Diary		10min Diary		10min Diary	10min Diary

Note: a = Filling in Session Rating Scale; b = Filling in Children's Feeling scale and Fun and Demand

For peer review only

Table S4. Computerized training (CogniFit Inc © 2022)

Game	Trained skills			
	flexibility	working memory	planning	inhibition
Traffic Manager			x	
Water Lilies		x	x	
Reaction Field	x			x
Happy Hopper				x
Neuron Madness				x
Minus Malus	x	x		
Tennis Bowling	x			
Candy Line Up		x	x	
Bee Balloon	x			
Slice and Drop			x	
Cube Foundry			x	

Note: x = *primarily trained functions,* x = *additionally trained functions.*

For peer review only

Table S5. Analog training

Game	Trained skills				Players
	flexibility	working memory	planning	inhibition	
Gold (Game Factory ®)	x	x	x		2-5
Dodelido (“Drei Magier ®”)	x	x		x	2-6
Särge schubsen (“Drei Magier ®”)	x	x	x	x	2-6
Geistesblitz 2.0 (“Zoch”)	x	x		x	2-8
Beaver Gang (“Amigo Spiele”)	x	x	x		2-6
Beaver Clan (“Amigo Spiele”)	x	x	x		2-6
Memo Dice (“Amigo Spiele”)	x	x	x		2-4
5er finden (HABA ®)	x		x		1-4
The nasty 7 (“Drei Hasen in der Abendsonne”)	x	x		x	2-6
Yokai (Game Factory ®)	x	x	x		2-4
Okiya (“Pegasus Spiele”)	x		x		2
Perplexus Crazy run (2 PE Original, 5 FT 208, 6 PE Epic)				x	1
Thunder Pops				x	1

Games selected based on https://www.spielendfoerdern.ch/_files/ugd/8fa6b3_f84a167cfa3c405f8d3244a83242e7ac.pdf

For peer review only

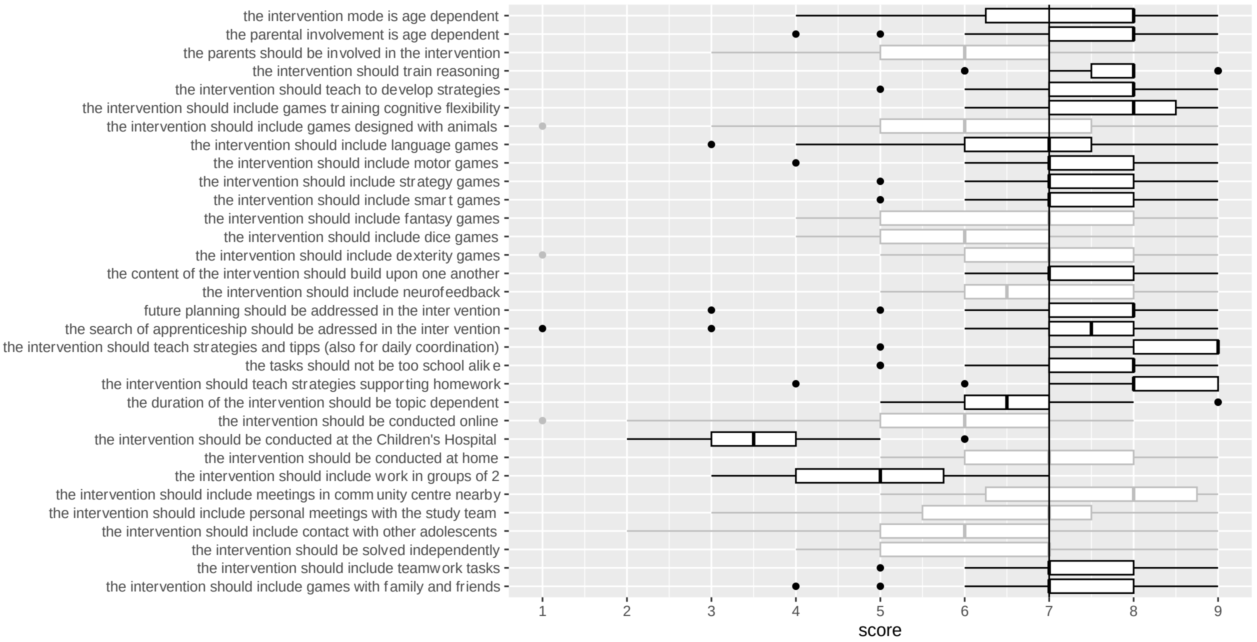

Figure S1. Ratings on Delphi items. 1 = fully disagree, 9 = fully agree. With a median of 7 and an IQR < 2 the item was considered accepted by the participants. Items in gray are the items for which no consensus was found.

For peer review only - http://bmjopen.bmj.com/site/about/guidelines.xhtml

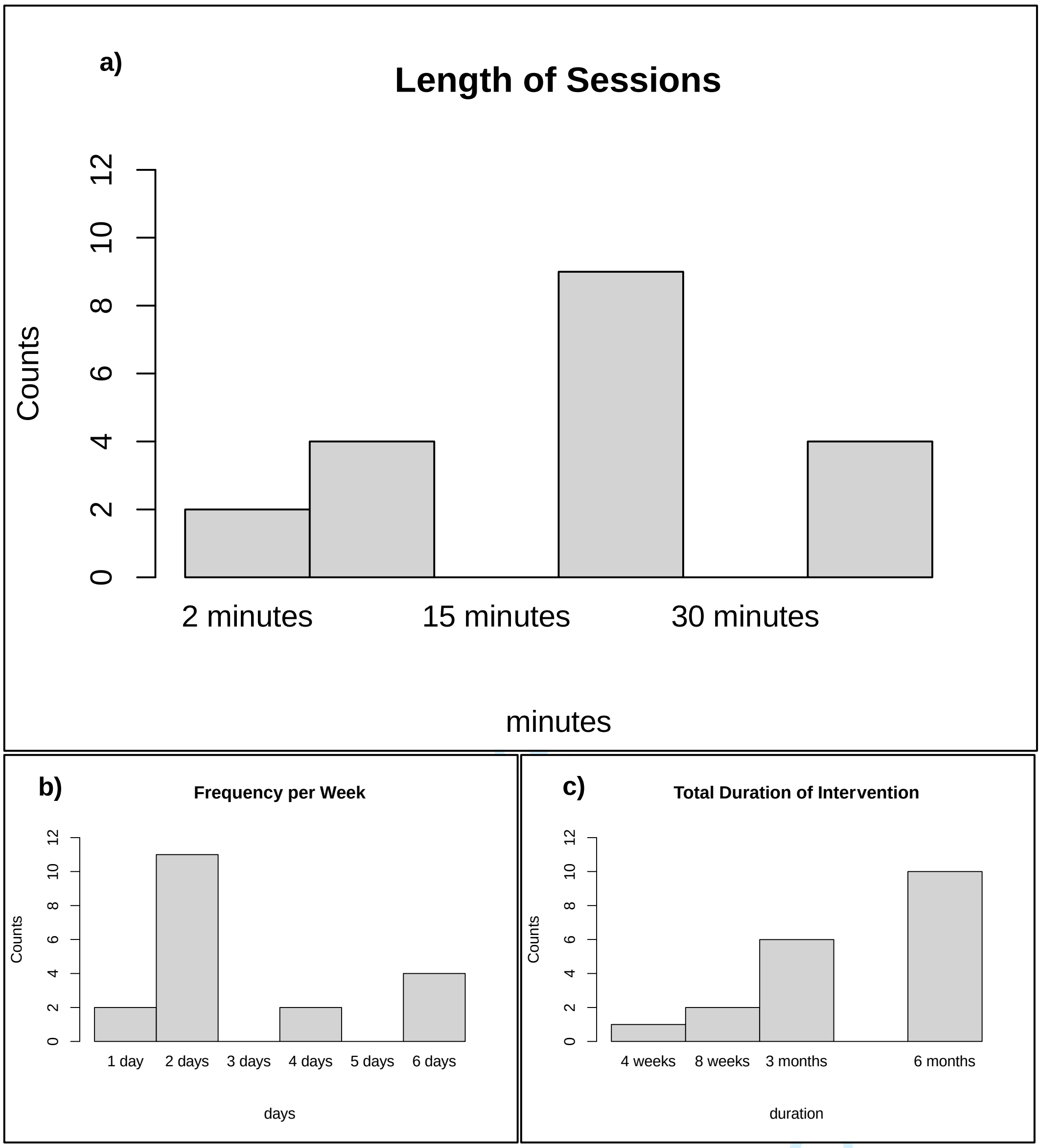

Figure S2. Counts of votes for a) length of sessions, b) frequency per week, and c) duration of the intervention.

For peer review only - http://bmjopen.bmj.com/site/about/guidelines.xhtml

STANDARD PROTOCOL ITEMS: RECOMMENDATIONS FOR INTERVENTIONAL TRIALS

SPIRIT 2013 Checklist: Recommended items to address in a clinical trial protocol and related documents*

Section/item	Item No	Description	Page No
**Administrative information**			
Title	1	Descriptive title identifying the study design, population, interventions, and, if applicable, trial acronym	1
Trial registration	2a	Trial identifier and registry name. If not yet registered, name of intended registry	2
	2b	All items from the World Health Organization Trial Registration Data Set	See https://register.clinicaltrials.gov/teenheart
Protocol version	3	Date and version identifier	1
Funding	4	Sources and types of financial, material, and other support	19
Roles and responsibilities	5a	Names, affiliations, and roles of protocol contributors	19, 6_ Appendix p.2
	5b	Name and contact information for the trial sponsor	1
	5c	Role of study sponsor and funders, if any, in study design; collection, management, analysis, and interpretation of data; writing of the report; and the decision to submit the report for publication, including whether they will have ultimate authority over any of these activities	16
	5d	Composition, roles, and responsibilities of the coordinating centre, steering committee, endpoint adjudication committee, data management team, and other individuals or groups overseeing the trial, if applicable (see Item 21a for data monitoring committee)	2_Appendix p.2
**Introduction**			
Background and rationale	6a	Description of research question and justification for undertaking the trial, including summary of relevant studies (published and unpublished) examining benefits and harms for each intervention	p. 4-5
	6b	Explanation for choice of comparators	n.a.
Objectives	7	Specific objectives or hypotheses	p. 6

For peer review only - http://bmjopen.bmj.com/site/about/guidelines.xhtml

Trial design	8	Description of trial design including type of trial (eg, parallel group, crossover, factorial, single group), allocation ratio, and framework (eg, superiority, equivalence, noninferiority, exploratory)	p. 9
**Methods: Participants, interventions, and outcomes**			
Study setting	9	Description of study settings (eg, community clinic, academic hospital) and list of countries where data will be collected. Reference to where list of study sites can be obtained	p. 9
Eligibility criteria	10	Inclusion and exclusion criteria for participants. If applicable, eligibility criteria for study centres and individuals who will perform the interventions (eg, surgeons, psychotherapists)	p. 9
Interventions	11a	Interventions for each group with sufficient detail to allow replication, including how and when they will be administered	p.10-12 4_Appendix
	11b	Criteria for discontinuing or modifying allocated interventions for a given trial participant (eg, drug dose change in response to harms, participant request, or improving/worsening disease)	n.a.
	11c	Strategies to improve adherence to intervention protocols, and any procedures for monitoring adherence (eg, drug tablet return, laboratory tests)	4_Appendix
	11d	Relevant concomitant care and interventions that are permitted or prohibited during the trial	n.a.
Outcomes	12	Primary, secondary, and other outcomes, including the specific measurement variable (eg, systolic blood pressure), analysis metric (eg, change from baseline, final value, time to event), method of aggregation (eg, median, proportion), and time point for each outcome. Explanation of the clinical relevance of chosen efficacy and harm outcomes is strongly recommended	p.12-13
Participant timeline	13	Time schedule of enrolment, interventions (including any run-ins and washouts), assessments, and visits for participants. A schematic diagram is highly recommended (see Figure)	p.19
Sample size	14	Estimated number of participants needed to achieve study objectives and how it was determined, including clinical and statistical assumptions supporting any sample size calculations	p.13-14

For peer review only

Recruitment	15	Strategies for achieving adequate participant enrolment to reach target sample size	p.9
**Methods: Assignment of interventions (for controlled trials)**			
Allocation:			
Sequence generation	16a	Method of generating the allocation sequence (eg, computer-generated random numbers), and list of any factors for stratification. To reduce predictability of a random sequence, details of any planned restriction (eg, blocking) should be provided in a separate document that is unavailable to those who enrol participants or assign interventions	p.10
Allocation concealment mechanism	16b	Mechanism of implementing the allocation sequence (eg, central telephone; sequentially numbered, opaque, sealed envelopes), describing any steps to conceal the sequence until interventions are assigned	p.10
Implementatio n	16c	Who will generate the allocation sequence, who will enrol participants, and who will assign participants to interventions	p.10
Blinding (masking)	17a	Who will be blinded after assignment to interventions (eg, trial participants, care providers, outcome assessors, data analysts), and how	p.10
	17b	If blinded, circumstances under which unblinding is permissible, and procedure for revealing a participant's allocated intervention during the trial	n.a.
**Methods: Data collection, management, and analysis**			
Data collection methods	18a	Plans for assessment and collection of outcome, baseline, and other trial data, including any related processes to promote data quality (eg, duplicate measurements, training of assessors) and a description of study instruments (eg, questionnaires, laboratory tests) along with their reliability and validity, if known. Reference to where data collection forms can be found, if not in the protocol	6_Appendix p.2
	18b	Plans to promote participant retention and complete follow-up, including list of any outcome data to be collected for participants who discontinue or deviate from intervention protocols	6_Appendix p.2
Data management	19	Plans for data entry, coding, security, and storage, including any related processes to promote data quality (eg, double data entry; range checks for data values). Reference to	6_Appendix p.2

For peer review only - http://bmjopen.bmj.com/site/about/guidelines.xhtml

		where details of data management procedures can be found, if not in the protocol	
Statistical methods	20a	Statistical methods for analysing primary and secondary outcomes. Reference to where other details of the statistical analysis plan can be found, if not in the protocol	p.14
	20b	Methods for any additional analyses (eg, subgroup and adjusted analyses)	n.a.
	20c	Definition of analysis population relating to protocol non-adherence (eg, as randomised analysis), and any statistical methods to handle missing data (eg, multiple imputation)	p.14
**Methods: Monitoring**			
Data monitoring	21a	Composition of data monitoring committee (DMC); summary of its role and reporting structure; statement of whether it is independent from the sponsor and competing interests; and reference to where further details about its charter can be found, if not in the protocol. Alternatively, an explanation of why a DMC is not needed	p. 19, 6_Appendix p.2,
	21b	Description of any interim analyses and stopping guidelines, including who will have access to these interim results and make the final decision to terminate the trial	n.a.
Harms	22	Plans for collecting, assessing, reporting, and managing solicited and spontaneously reported adverse events and other unintended effects of trial interventions or trial conduct	6_Appendix p.2
Auditing	23	Frequency and procedures for auditing trial conduct, if any, and whether the process will be independent from investigators and the sponsor	n.a.
**Ethics and dissemination**			
Research ethics approval	24	Plans for seeking research ethics committee/institutional review board (REC/IRB) approval	p.15
Protocol amendments	25	Plans for communicating important protocol modifications (eg, changes to eligibility criteria, outcomes, analyses) to relevant parties (eg, investigators, REC/IRBs, trial participants, trial registries, journals, regulators)	6_Appendix p.3
Consent or assent	26a	Who will obtain informed consent or assent from potential trial participants or authorised surrogates, and how (see Item 32)	p.9
	26b	Additional consent provisions for collection and use of participant data and biological specimens in ancillary studies, if applicable	n.a.

For peer review only

Confidentiality	27	How personal information about potential and enrolled participants will be collected, shared, and maintained in order to protect confidentiality before, during, and after the trial	6_Appendix p.2
Declaration of interests	28	Financial and other competing interests for principal investigators for the overall trial and each study site	p. 1
Access to data	29	Statement of who will have access to the final trial dataset, and disclosure of contractual agreements that limit such access for investigators	6_Appendix p.2
Ancillary and post-trial care	30	Provisions, if any, for ancillary and post-trial care, and for compensation to those who suffer harm from trial participation	n.a.
Dissemination policy	31a	Plans for investigators and sponsor to communicate trial results to participants, healthcare professionals, the public, and other relevant groups (eg, via publication, reporting in results databases, or other data sharing arrangements), including any publication restrictions	p.15
	31b	Authorship eligibility guidelines and any intended use of professional writers	6_Appendix p.2
	31c	Plans, if any, for granting public access to the full protocol, participant-level dataset, and statistical code	n.a.
**Appendices**			
Informed consent materials	32	Model consent form and other related documentation given to participants and authorised surrogates	3_Appendix
Biological specimens	33	Plans for collection, laboratory evaluation, and storage of biological specimens for genetic or molecular analysis in the current trial and for future use in ancillary studies, if applicable	n.a.

*It is strongly recommended that this checklist be read in conjunction with the SPIRIT 2013 Explanation & Elaboration for important clarification on the items. Amendments to the protocol should be tracked and dated. The SPIRIT checklist is copyrighted by the SPIRIT Group under the Creative Commons "Attribution-NonCommercial-NoDerivs 3.0 Unported" license.

For peer review only - http://bmjopen.bmj.com/site/about/guidelines.xhtml
